# Phytochemical profile and anti-inflammatory activity of the hull of γ-irradiated wheat mutant lines (*Triticum aestivum* L.)

**DOI:** 10.3389/fnut.2023.1334344

**Published:** 2023-12-22

**Authors:** Jisu Park, Yun-Seo Kil, Ga-Hee Ryoo, Chang Hyun Jin, Min Jeong Hong, Jin-Baek Kim, Chan-Hun Jung, Joo-Won Nam, Ah-Reum Han

**Affiliations:** ^1^Advanced Radiation Technology Institute, Korea Atomic Energy Research Institute, Jeongeup-si, Jeollabuk-do, Republic of Korea; ^2^College of Pharmacy, Yeungnam University, Gyeongsan-si, Gyeongsangbuk-do, Republic of Korea; ^3^Jeonju AgroBio-Materials Institute, Jeonju-si, Jeollabuk-do, Republic of Korea

**Keywords:** wheat hull, *Triticum aestivum*, UPLC-ESI-QTOF-MS, metabolomics, flavonolignan, anti-inflammatory

## Abstract

Wheat (*Triticum aestivum* Linn.; Poaceae) is the second most cultivated food crop among all global cereal crop production. The high carbohydrate content of its grains provides energy, multiple nutrients, and dietary fiber. After threshing, a substantial amount of wheat hull is produced, which serves as the non-food component of wheat. For the valorization of these by-products as a new resource from which functional components can be extracted, the hull from the seeds of cultivated wheat mutant lines bred after γ-irradiation were collected. Untargeted metabolite analysis of the hull of the original cultivar (a crossbreeding cultivar., Woori-mil × D-7) and its 983 mutant lines were conducted using ultra-performance liquid chromatography–electrospray ionization quadrupole time-of-flight mass spectrometry technique. A total of 55 molecules were tentatively identified, including 21 compounds found in the *Triticum* species for the first time and 13 compounds not previously described. Among them, seven flavonolignans with a diastereomeric structure, isolated as a single compound from the hull of *T. aestivum* in our previous study, were used as the standards in the metabolite analysis. The differences in their collision cross-section values were shown to contribute to the clear distinction between tricine-lignan stereoisomers. To select functionally active agents with anti-inflammatory activity among the identified compounds, the wheat hull samples were evaluated for their inhibitory effect on nitric oxide production in lipopolysaccharide-stimulated RAW 264.7 cells. As a result of multivariate analysis based on the results of chemical and biological profiles of the wheat hull samples, 10 metabolites were identified as key markers, contributing to the distinction between active and inactive mutant lines. Considering that one of the four key markers attributed to anti-inflammatory activity has been identified to be a flavonolignan, the wheat hull could be a valuable source of diverse tricin-lignan type compounds and used as a natural health-promoting product in food supplements.

## 1 Introduction

Wheat (*Triticum aestivum* L.; Poaceae) is the second most common crop that is cultivated worldwide. It is an important cereal as it contributes approximately 20% of the nutrient calories and proteins consumed by the global population (1). Wheat is a staple grain that consists of the endosperm, which is mostly starch, and the bran, which contains fiber, minerals, and phytochemicals (2). Whole wheat-grain product (or wheat bran extract) is reported to possess health-promoting properties such as antioxidant, anticancer, anti-diabetic, and antihypertensive activities (3–5), correlating with diverse phytochemicals content, such as flavonoids, phenolic acids, lignans, sphingolipids, and phytosterols (3). As a by-product of the grain threshing process, a large amount of wheat hull (i.e., *ca.* 10 million tones in Europe) is produced and discarded once the grain is obtained (6). Wheat by-products, including hull (husk) and straw, have evolved industrially from feed and bedding materials for livestock to advanced raw materials for the production of fuel ethanol, geopolymers, and biodegradable film (7–10). Additionally, the wheat hull has been reported as a valuable source rich in tricin (11), a flavone with multiple biological functions such as anticancer, anti-inflammatory, anti-obesity, and anti-diabetic properties (11–16). In addition, flavonolignans consisting of tricin and a lignin moiety (phenylpropanoid) were first isolated from the hull of wheat in our previous study (17). Since flavonolignans with anti-inflammatory activity have been found in the hull extract of wheat, it further encourages a more detailed metabolite analysis using advanced analytical instruments on wheat hulls.

Plant metabolomics research has recently been done in the fields of phytochemistry, plant genomics, and crop breeding (18–23). Ultra-performance liquid chromatography–electrospray ionization quadrupole time-of-flight mass spectrometry (UPLC-ESI QTOF MS) is a versatile technique for the identification of metabolites by applying fragment patterns of TOF mass spectrometry (MS/MS) spectra in combination with the retention time or ultraviolet (UV) spectrum data (24, 25). In addition, UPLC-ESI QTOF MS-based metabolomics is useful for the rapid and highly sensitive detection of secondary metabolites from a variety of natural sources, including plants, marine organisms, and microorganisms (26–29). Interestingly, technology that uses high-resolution mass spectrometry (HRMS) has improved detection and quantification strategies for assessing structural information to reveal diverse or unknown metabolites. The data-independent acquisition mode (DIA, termed MS^E^ on SYNAPT XS Q-TOF, Waters Corporation, Milford, MA, United States) affiliated with UPLC-ESI QTOF MS^E^ further improves the accuracy in metabolite identification since it can simultaneously acquire low- and high-energy spectra of each detected feature in a single run to provide information on the precursor and fragment ions (30, 31). Furthermore, the Ion Mobility Separation (IMS) function in QTOF MS provides an additional dimension of ion separation through the drift time (dt), later converted into collision cross-section value (CCS, Å^2^), to the conventional three-dimensional data formed by retention time (t_R_), accurate mass (*m/z*), and intensity from HRMS. It was recently proposed as another method for investigating the isomeric properties of secondary metabolites derived from plant material. In fact, the CCS value is being added to the compound databases together with the spectral information, and various CCS prediction tools are being developed to assist the compound identification process (32). In this study, an untargeted metabolic approach combined with UPLC-IMS-HRMS was utilized to identify the CCS values of isolated diastereomers.

Mutation breeding by γ-irradiation has been widely used to improve crops, including improving agronomic characteristics and diverse phenotypes (33–36). In wheat, γ-irradiation induced changes at the morphological, physiological, and agronomic levels, such as late maturing mutations, tall mutations, glaucoma mutations associated with improved crop tolerance to drought and heat and yield, branched spike mutations associated with ensuring grain uniformity per spike and abundant grain formation, and spike hairs associated with survival under biotic or abiotic stress (33). A variety of wheat with salinity tolerance by application of γ-rays has also been developed, and this mutant line has better osmotic pressure control, leaf relative water content, potassium ion concentration, soluble sugar content, and lower proline and glycine betaine content than the parent under salinity conditions (34). Furthermore, γ-irradiated mutagenesis has been used to generate genetic variants for breeding studies. In developed wheat mutant cultivar with higher kernel weight, a quantitative trait locus for thousand kernel weight, QTkw.cau-5D, which could be a target for genetic improvement of wheat grain weight, was identified (35). Our research group has developed wheat mutant lines generated by γ-irradiated mutagenesis, which exhibited diverse phenotypes with different plant heights and colors (36). Although the metabolite composition of several parts of wheat, including grain (or wholegrain), bran, and sprout, has been extensively reported (2, 3, 37–39), the chemical profile of the hull part is still not fully characterized. Therefore, the hulls of γ-irradiated wheat mutant lines were collected for untargeted metabolite analysis using the LC–MS/MS method. In addition, the untargeted metabolite profile of 985 wheat hull extracts and their nitric oxide (NO) production inhibitory activities against lipopolysaccharide (LPS)-activated RAW 264.7 cells were compared to select improved wheat mutant lines, and multivariate statistical analyses on these results were performed to identify marker compounds with anti-inflammatory activity.

## 2 Materials and methods

### 2.1 Chemicals

Dulbecco’s modified Eagle’s medium (DMEM), fetal bovine serum (FBS), and penicillin–streptomycin were purchased from Hyclone (Logan, UT, United States). LPS, dimethyl sulfoxide (DMSO), and Griess reagent were purchased from Sigma-Aldrich (St. Louis, MO, United States). All chemicals and solvents used in the extraction of the wheat hull samples were purchased from J. T. Baker (Phillipsburg, NJ, United States). Acetonitrile, methanol, water, and formic acid were of LC–MS grade (Thermo Fisher Scientific, Inc., Rockford, IL, United States).

### 2.2 Plant materials

Seeds of *T. aestivum* were sown and cultivated in constant soil conditions of the experimental field situated at 35.5699° N latitude by 126.9722° E longitude at the Advanced Radiation Technology Institute, Korea Atomic Energy Research Institute (Jeongeup-si, Jeollabuk-do 56,212, Korea). Spikes were labeled at flowering time, and the crop was harvested 50 days after flowering (July 2020). This plant was bred and identified by Min Jeong Hong and Jin-Baek Kim, co-authors of this study. The remaining hull, after threshing, was collected, dried at room temperature, and ground for further investigation. The voucher specimens were deposited at the Advanced Radiation Technology Institute, Korea Atomic Energy Research Institute.

### 2.3 Extraction and sample preparation

A total of 985 dried hull samples were soaked in methanol at a ratio of 200 mL methanol per 10 g dry weight, extracted by ultra-sonication (300 μm) for 60 min at 40°C, filtered using a filter paper, and concentrated to dryness using a rotary evaporator (40°C, 80 rpm). All samples were dissolved in an LC–MS grade methanol (10 mg/mL) and filtered using a 0.20-μm polyvinylidene fluoride filter before being subjected to UPLC-QTOF MS analysis. For the evaluation of bioactivity, each dried extract was initially dissolved in dimethyl sulfoxide (DMSO) at a concentration of 50 mg/mL.

### 2.4 UPLC-QTOF MS analysis

The extracts were analyzed on a BEH C18 chromatography column (100 mm × 2.1 mm i.d., 1.7 μm particle size, Waters Corporation, Milford, MA, United States) using a Waters ACQUITY UPLC system (Waters Corporation, Milford, MA, United States) equipped with a binary solvent delivery system, an autosampler, and a UV detector. UV–vis absorption spectra were recorded online from 200 to 500 nm during UPLC analysis. Aliquots (1 μL) of each sample were injected at a flow rate of 0.4 mL/min. The column temperature was kept constant at 40°C, and the autosampler temperature was 15°C. Gradient elution was carried out with water (A)/acetonitrile (B) containing 0.1% formic acid. The linear gradient elution program was as follows: 0–20.0 min, 17–65% B; 20.0–21.0 min, 65–100% B; 21.0–26.0 min, 100% B; 26.0–26.1 min, 100–17% B; 26.1–30.0 min, 17% B. The total run time, including re-equilibration of the column to the initial conditions, was 30 min. Detection was performed using a SYNAPT XS Q-TOF (Waters Corporation, Milford, MA, United States) mass spectrometer system equipped with traveling wave ion mobility. Electrospray ionization was performed in negative ionization mode with the following parameters: source temperature, 120°C; desolvation temperature, 450°C; capillary voltage, 2.0 kV; cone voltage, 25 V; cone gas flow, 50 L/h; and flow rate of desolvation gas (N_2_), 800 L/h. Argon was used as the collision gas, and nitrogen was used as the nebulizer and desolvation gas. Data were acquired for a mass scan range of *m/z* 100–1,200 Da in resolution mode with a 0.5-s scan time.

Data were acquired in high definition MS^E^ mode (HDMS^E^), in which the instrument switches between two energy channels: at low collision energy with 6 eV and a high energy with ramp 20–40 eV for a mass range of *m/z* 100–1,200Da to obtain precursor and product ion information in separate spectra but within a single acquisition. Because the ion mobility cell is positioned before the collision chamber in the SYNAPT XS Q-TOF (Waters Corporation, Milford, MA, United States) mass spectrometer equipped with traveling wave ion mobility, the precursor ion and its corresponding fragments have the same arrival time. External reference, leucine–enkephalin, at a concentration of 200 pg./μL in acetonitrile:water (1:1; *v*/*v*) + 0.1% formic acid was infused at a rate of 10 μL/min through a LockSpray channel automatically through the reference lock mass sprayer ([M – H]^−^ ion, *m*/*z* 554.2615). IMS mode was performed in negative ionization mode with the following parameters: IMS wave velocity, start velocity 1,000 m/s and end velocity 300 m/s; IMS wave height, 40 V. Accurate mass and elemental composition were calculated using MassLynx software (Waters Corp.) incorporated in the instrument. MS/MS analysis was also performed under the same conditions used for metabolite scanning, and CCS values were carried out on UNIFI v.1.9 Scientific Information System (Waters, Manchester, United Kingdom). Polyalanine was used for the CCS calibration of SYNAPT XS instruments. For stability, it was ensured that the variation in the *m*/*z* and CCS measurements was less than 5 ppm and less than 2%, respectively. Data acquisition and processing were performed with the MassLynx 4.1 software.

### 2.5 Feature-based molecular networking analysis

The acquired UPLC-QTOF-MSE data-independent acquisition (DIA) was analyzed for feature-based molecular networking (FBMN) based on the online FBMN-Progenesis QI workflow (40). Raw LC–MS/MS data was processed in the Progenesis QI software (version 3.0, Nonlinear Dynamics, Newcastle upon Tyne, United Kingdom) for alignment, peak-picking, and deconvolution. Both the feature quantification table (CSV file) and MS/MS spectral fragment summary (MSP file) of the raw LC–MS/MS data were exported from the Progenesis QI and submitted to the Global Natural Product Social (GNPS) platform (41). The configurations of network parameters were as follows: Precursor ion mass tolerance and fragment ion mass tolerance, 0.50; minimum cosine score, 0.70; minimum matched fragment ions, 6; maximum number of neighbor nodes for one single node, 10; and maximum size of a spectral family, 100.

### 2.6 Chemometric data analysis

The raw data were imported to Progenesis QI for pretreating, including normalization, peak alignment, and peak picking. The parameters included a retention time range of 0–22.0 min and a mass range from 100 to 1,200 Da. The resultant datasets of samples, comprising the sample code, *m*/*z*, peak RT, and peak areas, were imported into the EZinfo software package (Version 14.1 Umetrics, Umea, Sweden) to conduct chemometrics, including principal component analysis (PCA) and orthogonal partial least squares-discriminant analysis (OPLS-DA). PCA, an unsupervised pattern recognition pattern, reduces the dimension of the data matrix and converts the original variables into a new independent variable called principal components (PCs). It reveals the interrelationships between different variables and interprets sample patterns, groupings, similarities, and differences. OPLS-DA uses the class membership to maximize the variation, introduce an orthogonal signal correction (OSC) filter to separately handle the systematic variation correlated to or uncorrelated to the Y variable, and therefore, have better discriminant ability for the samples with larger within-class divergence than PCA. The quality and reliability of these models are usually evaluated with R^2^ and Q^2^. Their values range from 0 to 1, where 1 indicates perfect fitness and predictivity of models. Variable importance for projection (VIP) values generated from the OPLS-DA model indicate the most important variables for classification.

### 2.7 Cell viability

Cell viability was performed using the EZ-Cytox cell viability assay kit (DAEIL Lab, Seoul, Korea). The cells were incubated at 37°C, 5% CO_2_ for 24 h after seeding into 96-well plates at a density of 2 × 10^5^ cells/mL. The cells were then mixed with methanol extracts of wheat hull (50 μg/mL) and incubated for an additional 24 h. Following the incubation, 10 μL EZ-Cytox solution from the cell viability assay kit was added to each well and incubated for another 4 h. The relative cell viability was determined by measuring the formazan production using a spectrophotometer (Bio-Rad, Hercules, CA, United States) at an absorbance of 480 nm with a reference wavelength of 650 nm.

### 2.8 Measurement of NO production

RAW264.7 macrophage cells were maintained in DMEM, supplemented with 10% fetal bovine serum, 100 U/mL penicillin, and 100 μg/mL streptomycin at 37°C in a 5% CO_2_ incubator. The cells were seeded into 96-well plates at a density of 2 × 10^5^ cells/mL, then incubated at 37°C for 24 h. The cells were pretreated for 2 h with methanol extracts of wheat hull (50 μg/mL), following which they were stimulated for 18 h with 1 μg/mL of lipopolysaccharide (LPS). The nitrite concentration in the media was measured using a Griess reagent (Sigma-Aldrich, ST. Louis, MO, United States). After incubation, 100 μL of each supernatant was collected and mixed with 100 μL of Griess reagent. The absorbance of each solution was measured at 540 nm using a spectrophotometer (Bio-Rad, Hercules). The nitrite concentration was determined by comparison with a sodium nitrite standard curve. The percentage inhibition was calculated by the Equation (1):


(1) $$\begin{array}{l} {}[1-(\mathrm{NO}\;\mathrm{level}\;\mathrm{of}\;\mathrm{test}\;\mathrm{samples}÷\mathrm{NO}\;\mathrm{level}\;\mathrm{of}\;\mathrm{vehicle}\\ -\mathrm{treated}\;\mathrm{control})]\times 100\; \end{array}$$

### 2.9 Statistical analyses

All experiments were replicated at least three times to obtain means and standard deviations. Statistical significance was determined with analysis of variance using the multiple comparisons method of one-way ANOVA with Tukey’s post-hoc test using Prism 5.0 (GraphPad Software, Inc., San Diego, CA, United States).

## 3 Results and discussion

### 3.1 Identification of metabolites in wheat hull samples using UPLC-QTOF MS

**Figure 1:**
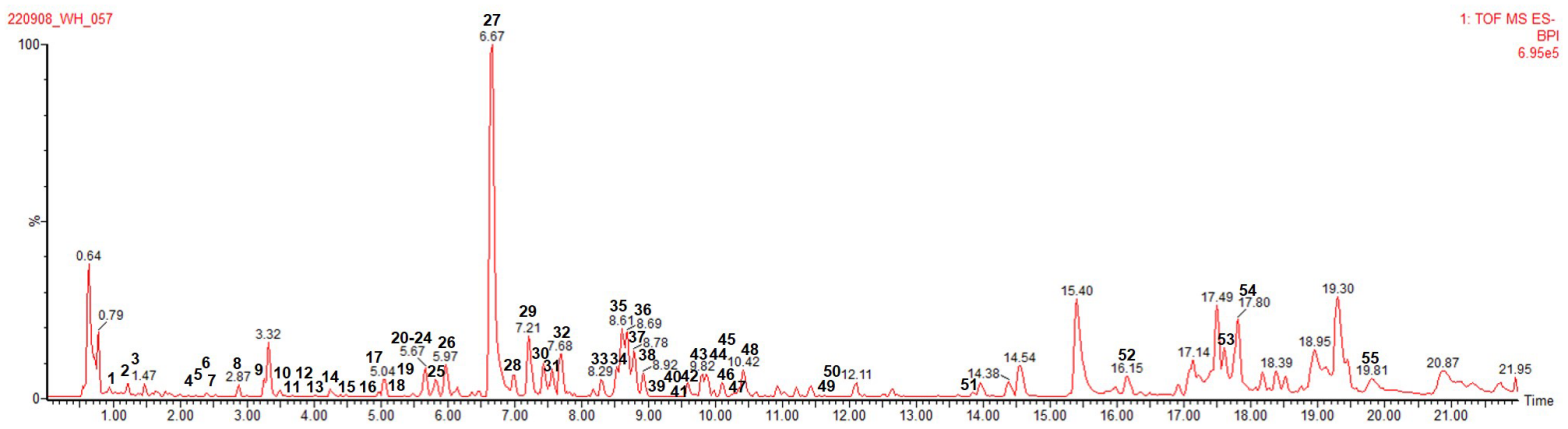
LC–MS base peak ion (BPI) chromatogram of the reprehensive hull extract of wheat mutant line (WH50) at negative ion mode (6 eV, ESI^–^). The selected chromatographic peaks are annotated with peak numbers referred to in Table 1.

In this study, metabolites in 985 wheat hull extracts, including two original cultivars (WH01 and WH02) and the mutant lines, were analyzed by UPLC-ESI QTOF MS in negative ion mode to obtain accurate *m*/*z* and MS^E^ information. In Figure 1, a base peak intensity (BPI) chromatogram of the hull extract of one wheat mutant line (WH50) is presented as a representative. A total of 61 peaks were shown in the BPI chromatogram, and the mass spectrum of each peak was interpreted by analyzing the retention times (t_R_), a major molecular ion, and fragmentation patterns (Supplementary Figures S1–S55). Table 1 lists the t_R_ values, calculated and observed deprotonated molecular ions *m*/*z* [M − H]^−^, calculated error ppm, proposed molecular formula, and most characteristic MS/MS fragment ions. Metabolite profiling was performed by constructing an *in-house* library with molecular structure files of several constituents already reported in wheat and other plants. Consequently, 61 metabolites were tentatively identified in the wheat hull, of which seven flavonolignans were identified by comparison with single compounds isolated in our previous study.

**Table 1:** Characterization and tentative identification of metabolites found in wheat hull extracts using UPLC-ESI QTOF MS in negative mode.

Peak No.	t_R_ (min)	Observed mass (*m*/*z*)	Calculated mass (*m*/*z*)	Error (ppm)	Molecular formula	Key MS^E^ fragment ions (*m*/*z*)	Identification^1^	Literature
1	0.99	579.1344	579.1350	−3.1	C_26_H_28_O_15_	579 (60), 489 (8), 459 (22), 399 (20)	Luteolin-6-*C*-arabinoside-8-*C*-glucoside (Isocarlinoside)	(39)
2	1.22	563.1399	563.1401	−0.4	C_26_H_28_O_14_	563 (100), 503 (8), 473 (12), 443 (10), 383 (28), 353 (30)	Apigenin-6-*C*-arabinoside-8-*C-*glucoside (Isochaftoside)	(39)
3	1.30	593.1498	593.1506	−1.4	C_27_H_30_O_15_	593 (100), 473 (20), 413 (28)	Apigenin-8-C-glucoside-2"-O-glucoside (Isovitexin-2"-*O*-glucoside)	(39)
4	2.13	461.1084	461.1084	0.0	C_22_H_22_O_11_	461 (48), 371 (28), 341 (100)	Chrysoeriol-8-C-glucoside^a^	(42)
5	2.23	653.1711	653.1718	−1.1	C_29_H_34_O_17_	653 (18), 491 (2), 329 (100), 314 (4)	Tricin-7-O-sophoroside (or Tricin-5-*O*-glucoside-7-*O*-glucoside)	(43, 44)
6	2.28	461.1085	461.1084	0.2	C_22_H_22_O_11_	461 (30), 341 (100)	Chrysoeriol-8-C-galactoside^a^	
7	2.43	491.1177	491.1190	−2.7	C_23_H_24_O_12_	491 (60), 329 (100), 314 (14)	Tricin-5-*O*-glucoside^b^	(39, 45)
8	2.87	637.1761	637.1769	−1.3	C_29_H_34_O_16_	637 (20), 329 (100), 314 (6)	Tricin 7-*O*-rutinoside	(46)
9	3.25	491.1184	491.1190	−1.2	C_23_H_24_O_12_	491 (100), 476 (30), 461 (18), 343 (4), 329 (12), 313 (20), 299 (10)	Tricin-7-*O*-glucoside^b^	(39, 45)
10	3.59	687.1909	687.1925	−2.3	C_33_H_36_O_16_	687 (58), 329 (44), 299 (100)	Tricin-4'-*O*-(*β*-guaiacylglyceryl) ether 5-*O*-glucoside	
11	3.68	491.1183	491.1190	−1.4	C_23_H_24_O_12_	491 (68), 343 (8), 329 (98)	Tricin-4'-*O*-glucoside^b^	
12	3.85	657.1804	657.1819	−2.3	C_32_H_34_O_15_	657 (2), 495 (52), 329 (100)	Tricin 4'-*O*-(*threo*-*β*-*p*-hydroxyphenylglyceryl) ether 7-*O*-glucopyranoside^c^	
13	4.03	687.1920	687.1925	−0.7	C_33_H_36_O_16_	687 (10), 525 (86), 329 (100) 314 (12)	Tricin-4'-*O*-(*threo*-*β*-guaiacylglyceryl) ether 7-*O*-glucoside^d^	(22, 47, 48)
14	4.23	657.1794	657.1819	−3.8	C_32_H_34_O_15_	657 (2), 495 (28), 329 (100), 314 (22)	Tricin 4'-*O*-(*erythro*-*β*-*p*-hydroxyphenylglyceryl) ether 7-*O*-glucopyranoside^c^	
15	4.45	687.1916	687.1925	−1.3	C_33_H_36_O_16_	687 (6), 525 (56), 329 (100), 314 (12)	Tricin 4'-*O*-(*erythro*-*β*-guaiacylglyceryl) ether 7-*O*-glucopyranoside^d^	(22, 47, 48)
16	4.80	657.1816	657.1819	−0.5	C_32_H_34_O_15_	657 (12), 495 (10), 329 (100), 314 (20)	Tricin 4'-*O*-(*threo*-*β*-*p*-hydroxyphenylglyceryl) ether 7"-*O*-glucopyranoside^e^	
17	4.82	687.1917	687.1925	−1.2	C_33_H_36_O_16_	687 (22), 525 (38), 329 (100), 314 (18)	Tricin 4'-*O*-(*threo*-*β*-guaiacylglyceryl) ether 7"-*O*-glucopyranoside^f^	(49)
18	5.15	657.1814	657.1819	−0.8	C_32_H_34_O_15_	657 (16), 495 (2), 329 (100), 314 (16)	Tricin 4'-*O*-(*erythro*-*β*-*p*-hydroxyphenylglyceryl) ether 7"-*O*-glucopyranoside^e^	
19	5.19	687.1932	687.1925	1.0	C_33_H_36_O_16_	687 (28), 525 (20), 329 (100), 314 (20)	Tricin 4'-*O*-(*erythro*-*β*-guaiacylglyceryl) ether 7"-*O*-glucopyranoside^f^	(49)
20	5.24	657.1816	657.1819	−0.5	C_32_H_34_O_15_	657 (8), 329 (100), 314 (14)	Tricin-4'-*O*-(*threo*/*erythro*-*β*-*p*-hydroxyphenylglyceryl) ether 9"-*O*-*β*-D-glucopyranoside^e^	
21	5.31	687.1917	687.1925	−1.2	C_33_H_36_O_16_	687 (12), 329 (100), 314 (12)	Tricin-4'-*O*-(*β*-guaiacylglyceryl) ether 9"-*O*-*β*-D-glucopyranoside	(22)
22	5.44	687.1929	687.1925	0.6	C_33_H_36_O_16_	687 (18), 477 (32), 329 (100), 314 (12)	Natasin A 4'-*O*-*β*-guaiacyl-(7"-O-methyl)-glyceryl ether	
23	5.48	477.1175	477.1186	−2.3	C_26_H_22_O_9_	477 (70), 431 (38), 416 (24), 329 (100), 314 (12)	Natasin A	(50)
24	5.59	657.1821	657.1819	0.3	C_32_H_34_O_15_	657 (1), 477 (16), 329 (100), 313 (12), 299 (8)	Natasin A 4'-*O*-[*β*-*p*-hydroxyphenyl-(7"-O-methyl)-glyceryl] ether	
25	5.79	687.1924	687.1925	−0.2	C_33_H_36_O_16_	687 (1), 329 (100)	Tricin-2′,3-dihydropropanol-2′-(4-hydroxy-3-methoxyphenyl) 4'-*O*-[*β*-guaiacyl-(7"-*O*-methyl)-glyceryl] ether	
26	5.81	507.1289	507.1291	−0.4	C_27_H_24_O_10_	507 (88), 492 (28), 477 (8), 461 (30), 446 (18), 329 (100)	5,6-Dihydro-3,8,10-trihydroxy-5-(4-hydroxy-3-methoxyphenyl)-6-hydroxymethyl-2,4-dimethoxy-7H-benzo[c]xanthen-7-one	(51)
27	6.67	329.0662	329.0661	0.3	C_17_H_14_O_7_	329 (98), 314 (70), 299 (100), 271 (66), 227 (30)	Tricin	(11, 22)
28	7.00	495.1294	495.1291	0.6	C_26_H_24_O_10_	495 (26), 329 (100), 314 (46), 299 (28), 271 (10)	Tricin 4'-*O*-(*threo*-*β*-*p*-hydroxyphenylglyceryl) ether^g^	(52)
29	7.22	525.1399	525.1397	0.4	C_27_H_26_O_11_	525 (60), 329 (100), 314 (48), 299 (22), 271 (4)	Tricin-4'-*O*-(*threo*-*β*-guaiacylglyceryl) ether^h^	(47, 53, 54)
30	7.36	475.1241	475.1240	0.2	C_23_H_24_O_11_	475 (1), 313 (100), 298 (12)	Luteolin 3′,4′-dimethylether-7-*O*-glucoside	(55)
31	7.43	495.1288	495.1291	−0.6	C_26_H_24_O_10_	495 (26), 329 (100), 314 (46), 299 (26), 271 (6)	Tricin 4'-*O*-(*erythro*-*β*-*p*-hydroxyphenylglyceryl) ether^g^	(52)
32	7.68	525.1396	525.1397	−0.2	C_27_H_26_O_11_	525 (40), 329 (100), 314 (48), 299 (22), 271 (4)	Tricin-4'-*O*-(*erythro*-*β*-guaiacylglyceryl) ether^h^	(47, 53, 54)
33	8.30	523.1237	523.1240	−0.6	C_27_H_24_O_11_	523 (18), 329 (100), 314 (38), 299 (16)	Tricin-4'-*O*-(*C*-veratroylglycol) ether	(56)
34	8.52	329.2325	329.2328	−0.9	C_18_H_34_O_5_	329 (100), 229 (40), 211 (68)	Pinellic acid (isomer 1)	(57)
35	8.61	329.2328	329.2328	0.0	C_18_H_34_O_5_	329 (100), 229 (38), 211 (48)	Pinellic acid (isomer 2)	(57)
36	8.69	329.2327	329.2328	−0.3	C_18_H_34_O_5_	329 (100), 229 (20), 211 (36)	Pinellic acid (isomer 3)	(57)
37	8.78	329.2330	329.2328	0.6	C_18_H_34_O_5_	329 (100), 229 (22), 211 (40)	Pinellic acid (isomer 4)	(57)
38	8.92	329.2327	329.2328	−0.3	C_18_H_34_O_5_	329 (100)	5,8,12-Trihydroxy-trans-9-octadecenoic acid	(58)
39	9.15	509.1452	509.1448	0.8	C_27_H_26_O_10_	509 (1), 329 (100), 314 (46), 299 (28)	Tricin 4'-*O*-[*threo*-*β*-*p*-hydroxyphenyl (7"-*O*-methyl)-glyceryl] ether	(50)
40	9.37	509.1447	509.1448	−0.2	C_27_H_26_O_10_	509 (1), 329 (100), 314 (30)	Tricin 4'-*O*-[*erythro*-*β*-*p*-hydroxyphenyl-(7"-*O*-methyl)-glyceryl] ether	(50)
41	9.48	539.1539	539.1553	−2.6	C_28_H_28_O_11_	539 (8), 329 (100), 314 (30), 313 (14)	Tricin 4'-*O*-[*threo*-*β*-guaiacyl-(7"-*O*-methyl)-glyceryl] ether	(59)
42	9.55	539.1548	539.1553	−0.9	C_28_H_28_O_11_	539 (2), 329 (100), 314 (40), 299 (16)	Tricin 4'-*O*-[erythro*-β*-guaiacyl-(7"-*O*-methyl)-glyceryl] ether	(59)
43	9.59	567.1494	567.1503	−1.6	C_29_H_28_O_12_	567 (30), 329 (100), 314 (40), 299 (16)	Tricin 4'-*O*-[*erythro*-*β*-guaiacyl-(9"-*O*-acetyl)-glyceryl] ether	(60)
44	9.82	567.1499	567.1503	−0.7	C_29_H_28_O_12_	567 (30), 329 (100), 314 (38), 299 (12)	Tricin 4'-*O*-[*threo*-*β*-guaiacyl-(9"-*O*-acetyl)-glyceryl] ether	(60)
45	9.87	329.2330	329.2328	0.6	C_18_H_34_O_5_	329 (100)	Trihydroxy octadecenoic acid (isomer 1)	(57)
46	10.10	329.2327	329.2328	−0.3	C_18_H_34_O_5_	329 (100)	Trihydroxy octadecenoic acid (isomer 2)	(57)
47	10.35	329.2323	329.2328	−1.5	C_18_H_34_O_5_	329 (100)	Trihydroxy octadecenoic acid (isomer 3)	(57)
48	10.48	329.2329	329.2328	0.3	C_18_H_34_O_5_	329 (100)	Trihydroxy octadecenoic acid (isomer 4)	(57)
49	11.76	581.1648	581.1659	−1.9	C_30_H_30_O_12_	581 (12), 329 (100), 314 (34), 299 (14)	Tricin 4'-*O*-[*threo*-*β*-guaiacyl-(7"-*O*-methyl-9"-*O*-acetyl)-glyceryl] ether	(60)
50	11.90	581.1652	581.1659	−1.2	C_30_H_30_O_12_	581 (18), 329 (100), 314 (36), 299 (8)	Tricin 4'-*O*-[*erythro*-*β*-guaiacyl-(7"-*O*-methyl-9"-*O*-acetyl)-glyceryl] ether	(60)
51	13.96	313.2376	313.2379	1.0	C_18_H_34_O_4_	313 (100), 295 (44), 183 (78)	12,13-Dihydroxy-9*Z*-octadecenoic acid	(57)
52	16.15	562.3135	562.3145	−1.8	C_26_H_48_O_7_NP	562 (2), 277 (100), 224 (10)	1-(9*Z*,12*Z*,15*Z*-Octadecadienoyl)-*sn*-glycero-3-phosphocholine	(57)
53	17.61	295.2276	295.2273	−1.0	C_18_H_32_O_3_	295 (100), 277 (42), 195 (32)	9*E*,11*E*-13-Hydroxyoctadecadienoic acid	
54	17.81	564.3305	564.3301	0.7	C_26_H_50_O_7_NP	564 (12), 279 (100), 224 (10)	1-(9*Z*,12*Z*-Octadecadienoyl)-*sn*-glycero-3-phosphocholine	(57)
55	19.81	566.3456	566.3458	−0.4	C_26_H_52_O_7_NP	566 (1), 281 (50)	1-(9*Z*-Octadecenoyl)-*sn*-glycero-3-phosphocholine	(57)

UPLC-ESI QTOF MS, ultra-performance liquid chromatography–electrospray ionization quadrupole time-of-flight mass spectrometry; t_R_, retention time; MS^E^, data independent acquisition mode on waters instruments. ^1^Tentatively identified molecules with the same superscript letters (a–h) have the same major molecular ion and fragment ions but are in different conformations; thus, their retention times are interchangeable.

### 3.2 Flavonoid *C*-glycosides and flavonoid *O*-glycosides

Flavonoids commonly accumulate in plants as glycosylated derivatives, conjugated through *O*- or *C*-glycosidic bonds. Typically, flavonoids accumulate as *O*-glycosylated derivatives in most plants, while flavone-*C*-glycosides are predominantly synthesized by cereal crops. *O*-glycosides have been reported mainly as 3- and 7-*O*-glycosides; however, *C*-glycosides have been found as mostly 6- and 8-*C*-glycosides. The flavonoid *C*-glycosides have sugar substituents directly linked to the aglycone by C–C bonds. *O*-glycosyl-*C*-glycosyl flavones and *C*-glycosyl flavones *O*-glycosylated on the sugar moiety of the *C*-glycosylation are also found (61). The MS^2^ fragmentation of mono-*C*-hexosyl flavones showed characteristic ions with loss of 90 Da [M–H–90]^−^ and 120 Da [M–H–120]^−^, which were identical with molecular ions for aglycone +71 Da and aglycone +41 Da, respectively. A relatively high abundance of the ion [M − H − 90]^−^ was observed in the 6-*C*-hexosyl flavones, while a relatively high intensity of the ion [M − H − 120]^−^ in the 8-*C*-hexosyl flavones was noted. In addition, a loss of 18 Da [M–H–H_2_O]^−^ was usually observed in 6-*C*-hexosyl flavones and rarely detected in 8-*C*-hexosyl flavones (62, 63). The MS^2^ fragmentation of di-*C*-glycosyl flavones typically presented the ions for aglycone +113 Da and aglycone +83 Da. 6,8-di-*C*-glycosyl flavones with pentose and hexose exhibited [M − H − 60]^−^ (weak), [M − H − 90]^−^, and [M − H − 120]^−^ in their MS^2^ fragmentation; however, the fragment ion [M − H − 60]^−^ was not observed in 6,8-di-*C*-hexosyl flavones, indicating that the fragment ion [M − H − 60]^−^ originates from pentose. In addition, the 6-*C*-pentosyl-8-*C*-hexosyl flavones showed a relatively higher abundance of the ion [M − H − 90]^−^ than [M − H − 120]^−^, while the relatively high intensity of the ion [M − H − 120]^−^ was observed in 6-*C*-hexosyl-8-*C*-pentosyl flavones (64). *O*-glycosyl-*C*-glycosyl flavones exhibited the fragment ions of [M − H − 146]^−^ for pentoses, [M − H − 164]^−^ for rhamnoses, and [M − H − 162]^−^ for hexoses, along with the fragment pattern of *C*-glycosyl flavone. In contrast, *C*-glycosyl flavones *O*-glycosylated on the sugar moiety of the *C*-glycosylation site showed the fragment ions of [M − H − 150]^−^ for pentoses, [M − H − 164]^−^ for rhamnoses, and [M − H − 180]^−^ for hexoses. This *O*-glycosylation was mainly positioned at C-2″, followed by C-6″, and rarely at other positions (61).

The MS analysis of peak 1 (t_R_ = 0.99 min, mass error − 3.1 ppm) exhibited the major molecular ion at *m*/*z* 579.1332 [M − H]^−^, with the fragment ions in the MS/MS spectrum being observed at *m*/*z* 489.1016 [M − H − 90]^−^, 459.0920 [M − H − 120]^−^, and 399.0713 [aglycone+113]^−^, indicating di-*C*-glycosyl luteolin. The relatively higher intensity of the ions [M − H − 90]^−^ than the ion [M − H − 120]^−^ suggested the 6-*C*-pentosyl-8-*C*-hexosyl substitution. Based on these observations and comparison of its MS data with reported pieces of literature (39, 65), it was tentatively assigned as luteolin-6-*C*-arabinoside-8-*C*-glucoside (isocarlinoside), which has been found in wheat seedlings (*T. aestivum*) (39).

Peak 2 (t_R_ = 1.22 min, mass error − 0.4 ppm) was detected with a deprotonated molecular ion at *m*/*z* 563.1399. The fragmentation pattern of the MS/MS spectrum exhibited five main fragment ions at *m*/*z* 503.1187 [M − H − 60]^−^, 473.1077 [M − H − 90]^−^, 443.0977 [M − H − 120]^−^, 383.0760 [aglycone+113]^−^, and 353.0656 [aglycone+83]^−^, indicating di-*C*-glycosyl apigenin. The relative high intensity of the ions [M − H − 90]^−^ suggested 6-*C*-pentosyl-8-*C*-hexosyl substitution. Therefore, a comparison of its MS data with pieces of literature (39) confirmed the tentative identification of this peak as apigenin-6-*C*-arabinoside-8-*C*-glucoside (isochaftoside), which has been found in wheat seedlings (*T. aestivum*) (39).

The MS spectrum of peak 3 (t_R_ = 1.30 min, mass error − 1.4 ppm) presented major molecular ions at *m*/*z* 593.1498 [M − H]^−^, with the fragment ions in the MS/MS spectrum observed at *m*/*z* 473.1081 [M − H − 120]^−^, 413.0868 [M − H − 180]^−^, 383.0770 [aglycone+113]^−^, and 353.0670 [aglycone+83]^−^, respectively, suggesting *C*-glucosyl apigenin which is *O*-glucosylated on the *C*-glucoside. The relatively high intensity of the ion [M − H − 120]^−^ and the absence of the ion [M − H − 18]^−^ provided evidence that *C*-glucoside was positioned at C-8. This *O*-glycosylation was mainly positioned at C-2″. Based on these observations and comparison of its MS data with pieces of literature (66), peak 3 was tentatively assigned as apigenin-8-*C*-glucoside-2"-*O*-glucoside (isovitexin-2"-*O*-glucoside), which has been found in wheat seedlings (*T. aestivum*) (39).

Peak 4 (t_R_ = 2.13 min, mass error 0 ppm) and peak 6 (t_R_ = 2.28 min, mass error 0.2 ppm) possessed a major molecular ion [M − H]^−^ at *m*/*z* 461.1084 and two fragment ions at *m*/*z* 371.0750 [M − H-90]^−^ for chrysoeriol +71 Da and 341.0660 [M − H-120]^−^ for chrysoeriol +41 Da. The relatively high intensity of the ion [M − H − 120]^−^ and the absence of the ion [M–H–18]^−^ suggested the 8-*C*-hexosyl chrysoeriol. Thus, peaks 4 and 6 were tentatively identified as chrysoeriol-8-*C*-hexoside isomers by comparison of its MS data with the literature (42). Chrysoeriol-8-*C*-glucoside (scoparin) (peak 4) has been reported in rice (*Oryza sativa*) (42), however, not in wheat (*Triticum* species), whereas chrysoeriol-8-*C*-galactoside (peak 6) has not been described previously.

The MS analysis of peak 5 (t_R_ = 2.23 min, mass error − 1.1 ppm) exhibited the molecular ion at *m*/*z* 653.1711 [M − H]^−^ and fragment ions at *m/z* 491.1695 [M–H–162]^−^ and 329.0662 [M–H–324]^−^, respectively, by the loss of two glucose molecules. Thus, peak 5 was tentatively assigned as tricin-7-*O*-glucoside-2"-*O*-glucoside (tricin-7-*O*-sophoroside) or tricin-5-*O*-glucoside-7-*O*-glucoside. Tricin-7-*O*-glucoside-2"-*O*-glucoside (tricin-7-*O*-sophoroside) and tricin-5-*O*-glucoside-7-*O*-glucoside have been reported in alfalfa (*Medicago* species) (43) and teinturier grape (*Vitis vinifera*) (44), respectively, but not in wheat (*Triticum* species).

The product ion mass spectrum of peak 8 (t_R_ = 2.87 min, mass error − 1.3 ppm) exhibited a deprotonated ion at *m*/*z* 637.1761 [M − H]^−^, and the MS/MS profile was observed with a fragment ion at *m*/*z* 329.0658 [M − H − 308]^−^, suggesting the loss of a rutinoside. Further comparison of its MS data with literature (64) confirmed the tentative identification of this peak as tricin 7-*O*-rutinoside, which has been found in wheat aerial parts (*T. aestivum*) (46).

Peaks 7, 9, and 11 were detected with the same deprotonated molecular ion, but their fragmentation was different. The MS spectra of peak 7 (t_R_ = 2.43 min, mass error − 2.7 ppm) obtained in the negative ion mode revealed a major molecular ion at *m*/*z* 491.1177 [M − H]^−^ and a high intensity of fragment ion at *m*/*z* 329.0651 [M–H–162]^−^ by the loss of a glucose molecule. Peak 9 (t_R_ = 3.25 min, mass error − 1.2 ppm) showed a major molecular ion at *m*/*z* 491.1184 [M − H]^−^. The fragment ions produced at *m/z* 476.0953 [M–H–15] by the loss of the methyl group and 461.0722 [M − H–30]^−^ by the loss of the methoxy group were observed. Other fragment ions at *m*/*z* 329.0648 [M − H–162]^−^ were characterized as the losses of a glucoside. Peak 11 (t_R_ = 3.68 min, mass error − 1.4 ppm) showed a major molecular ion at *m*/*z* 491.1188 [M − H]^−^ and a high intensity of fragment ions at 329.0652 [M–H–162]^−^ by the loss of a glucose molecule and 313.0698 [M–H–162–18]^−^ by the loss of the hydroxyl group. Although the fragment ions of the three peaks were shown differently, in conclusion, the three peaks were identified as tricin-*O*-glucoside isomers in which glucosides were positioned at 5-, 7-, and 4′-hydroxy groups of tricin, respectively. Therefore, peaks 7, 9, and 11 were tentatively identified as tricin-5-*O*-glucoside, tricin-7-*O*-glucoside, and tricin-4'-*O*-glucoside, respectively, and these are interchangeable. Tricin-7-*O*-glucoside and tricin-5-*O*-glucoside have been reported in wheat seedlings (*T. aestivum*) (39, 45), whereas tricin-4'-*O*-glucoside has not been described previously.

Peak 30 (t_R_ = 7.36 min, mass error 0.2 ppm) exhibited a deprotonated ion at *m*/*z* 475.1241 [M − H]^−^, and the MS/MS profile was observed with a high intensity of fragment ion at *m*/*z* 313.0708 [M − H − 162]^−^ for tetrahydroxyflavone-dimethyl ether moiety without a hexose. Further comparison of its MS data with the literature (55) confirmed the tentative identification of this peak as luteolin 3′,4′-dimethylether-7-*O*-glucoside. This compound has been found earlier in *Stachys aegyptiaca* (55) but was first reported in the Poaceae, including the genus *Triticum*.

### 3.3 Flavonolignans (tricin-lignan derivatives)

Flavonolignans are composed of tricin, a flavone and guaiacyl or *p*-coumaryl moiety, and lignan (phenylpropanoid) and have been reported in several plants such as rice plants (*O. sativa* and *Zizania latifolia*), oat plants (*Avena sativa*), and rattan palm (*Calamus quiquesetinervius*), among others (67); however, the discovery of tricin-lignan type compounds in *Triticum* species was first reported in our previous study (17). Compounds that have been isolated from the wheat hull in our previous study were used as the standards in this metabolite analysis: tricin (peak 27) and seven flavonolignans: tricin 4'-*O*-[*threo*-*β*-*p*-hydroxyphenyl-(7"-*O*-methyl)-glyceryl] ether (peak 39), tricin 4'-*O*-[*erythro*-*β*-*p*-hydroxyphenyl-(7"-*O*-methyl)-glyceryl] ether (peak 40), tricin 4'-*O*-[*threo*-*β*-guaiacyl-(7"-*O*-methyl)-glyceryl] ether (peak 41), tricin 4'-*O*-[*erythro*-*β*-guaiacyl-(7"-*O*-methyl)-glyceryl] ether (peak 42), tricin 4'-*O*-[*threo*-*β*-guaiacyl-(9"-*O*-acetyl)-glyceryl] ether (peak 43), tricin 4'-*O*-[*threo*-*β*-guaiacyl-(7"-*O*-methyl-9"-*O*-acetyl)-glyceryl] ether (peak 49), and tricin 4'-*O*-[*erythro*-*β*-guaiacyl-(7"-*O*-methyl-9”"-*O*-acetyl)-glyceryl] ether (peak 50). In addition, the ion mobility separations (IMSs)-MS system was used for achieving intentional stereochemistry identification of these flavonolignan isomers. IMS provides CCS values by separating ionized molecules according to drift time, as well as high definition MS^E^ (HDMS^E^) as additional separation capabilities. Different CCS values and HDMS^E^ according to different stereochemistry of tricin-lignan isomers are presented in this study for the first time (Table 2; Supplementary Figures S56–S66).

**Table 2:** Collision cross-section (CCS) values of seven flavonolignan standards were measured using an ion mobility-MS system.

Peak No.	Observed t_R_ (min)	Molecular formula	Neutral mass (Da)	Observed *m/z* [M–H]^−^	Observed CCS (Å^2^)	Compound name
39	9.14	C_27_H_26_O_10_	510.153	509.1520	229.75	Tricin 4'-*O*-[*threo*-*β*-*p*-hydroxyphenyl-(7"-*O*-methyl)-glyceryl] ether
40	9.36	C_27_H_26_O_10_	510.153	509.1518	219.70	Tricin 4'-*O*-[*erythro*-*β*-*p*-hydroxyphenyl-(7"-*O*-methyl)-glyceryl] ether
41	9.47	C_28_H_28_O_11_	540.163	539.1641	237.84	Tricin 4'-*O*-[*threo*-*β*-guaiacyl-(7"-*O*-methyl)-glyceryl] ether
42	9.55	C_28_H_28_O_11_	540.163	539.1633	228.18	Tricin 4'-*O*-[*erythro*-*β*-guaiacyl-(7"-*O*-methyl)-glyceryl] ether
44	9.79	C_29_H_28_O_12_	568.158	567.1584	234.58	Tricin 4'-*O*-[*threo*-*β*-guaiacyl-(9"-*O*-acetyl)-glyceryl] ether
49	11.70	C_30_H_30_O_12_	582.174	581.1702	245.81	Tricin 4'-*O*-[*threo*-*β*-guaiacyl-(7"-*O*-methyl-9"-*O*-acetyl)-glyceryl] ether
50	11.86	C_30_H_30_O_12_	582.174	581.1736	239.87	Tricin 4'-*O*-[*erythro*-*β*-guaiacyl-(7"-*O*-methyl-9"-*O*-acetyl)-glyceryl] ether

CCS, collision cross-section value; t_R_, retention time.

Peak 27 (t_R_ = 6.67 min, mass error 0.3 ppm) showed a major [M − H]^−^ ions at *m*/*z* 329.0662 and fragment ions at *m*/*z* 314.0424 [M − H − CH_3_]^−^, 299.0198 [M − H − OCH_3_]^−^, 271.0243 [M − H − 2(CH_3_)]^−^, and 227.0347 [M − H − 2(OCH_3_)^−^ C_2_H_2_O], respectively, were observed in its MS/MS spectrum. Further comparison of its MS data with authentic compounds and the literature (11, 22) confirmed the tentative identification of this peak as tricin.

In the extracted ion chromatogram for *m/z* 509.1448, three main peaks appeared, and as a result of analyzing their MS, the peaks at 9.15 and 9.37 min were vailed as major molecular ions, and the peak at 5.81 min was observed as the fragment ion of very low intensity (Supplementary Figure S56). Peaks 39 (t_R_ = 9.15 min, mass error 0.8 ppm) possessed a molecular ion at *m*/*z* 509.1452 [M − H]^−^ and a major fragment ion at *m*/*z* 329.0656 [M − H − 180]^−^, representing the molecular weight of tricin in which the lignan group is not substituted. Comparison with the HRMS data of the original compound, 4'-*O*-[*threo*-*β*-*p*-hydroxyphenyl-(7"-*O*-methyl)-glyceryl] ether revealed that this fragment ion corresponded to the loss of the hydroxyphenylmethylglyceryl group. Thus, this peak was unequivocally identified as 4'-*O*-[*threo*-*β*-*p*-hydroxyphenyl-(7"-*O*-methyl)-glyceryl] ether (50). Peak 40 (t_R_ = 9.37 min, mass error − 0.2 ppm), appearing at a very similar retention time to that of peak 39, also gave a molecular ion at *m/z* 509.1477 [M − H]^−^ and a major fragment ion at *m*/*z* 329.0660 [M − H − 180]^−^, indicating the same molecular structure as peak 39. In addition, the molecule of this peak was clearly identified by comparing with the HRMS data of the standard compound, 4'-*O*-[*erythro*-*β*-*p*-hydroxyphenyl-(7"-*O*-methyl)-glyceryl] ether (50). Since these two compounds were diastereomers, their CCS values and HDMS^E^ were measured and compared with each other using an IMS-MS system. As a result, CCS values of 4'-*O*-[*threo*-*β*-*p*-hydroxyphenyl-(7"-*O*-methyl)-glyceryl] ether and 4'-*O*-[*erythro*-*β*-*p*-hydroxyphenyl-(7"-*O*-methyl)-glyceryl] ether were 229.75 and 219.70 Å^2^, respectively, indicating that the difference between the values was significantly higher than 2 Å^2^. In the HDMS^E^, the fragment ion at m/z 447.11401 [M − H − 62]^−^ by the loss of CH_2_OH and OCH_3_ moieties of the glyceryl group was observed; the intensity of this fragment ion in the *erythro*-form was characteristically higher than that in the *threo*-form (Supplementary Figures S57, S58).

In the extracted ion chromatogram for *m/z* 539.155, two peaks, 41 and 42, were shown as almost undivided peaks (Supplementary Figure S59). Peaks 41 (t_R_ = 9.48 min, mass error − 2.6 ppm) and 42 (t_R_ = 9.55 min, mass error − 0.9 ppm) exhibited the identical precursor ion at *m/z* 539.1539 [M − H]^−^ and the identical fragment ion at *m/z* 329.0653 [M − H − 210]^−^, corresponding to the loss of the guaiacylmethylglyceryl group. The MS/MS spectra of the precursor ion at m/z 539 [M − H]^−^ gave a product ion at *m/z* 329 [M − H − 210] ^−^, corresponding to the loss of the guaiacylmethylglyceryl group. Further comparison of the HRMS data of the original compounds, tricin 4'-*O*-[*threo*-*β*-guaiacyl-(7"-*O*-methyl)-glyceryl] ether (59) and tricin 4'-*O*-[*erythro*-*β*-guaiacyl-(7"-*O*-methyl)-glyceryl] ether (59) confirmed that peaks 41 and 42 belonged to the respective molecules. In the IMS-MS, tricin 4'-*O*-[*threo*-*β*-guaiacyl-(7"-*O*-methyl)-glyceryl] ether and tricin 4'-*O*-[*erythro*-*β*-guaiacyl-(7"-*O*-methyl)-glyceryl] ether showed their CCS values of 237.84 and 228.18 Å^2^, respectively, that differed by >2 Å^2^. The fragment ion at m/z 477.12597 [M − H − 62]^−^ in their HDMS^E^ corresponded to the loss of CH_2_OH and OCH_3_ moieties of the glyceryl group. As in the case of peaks 41 and 42, the intensity of this fragment ion in the *erythro*-form was higher than that in the *threo*-form (Supplementary Figures S60, S61).

In the extracted ion chromatogram for *m/z* 567.150, two main peaks, 43 and 44, were observed (Supplementary Figure S62). Peaks 43 (t_R_ = 9.59 min, mass error − 1.6 ppm) and 44 (t_R_ = 9.82 min, mass error − 0.7 ppm) exhibited the molecular ion at *m*/*z* 567.1494 [M − H]^−^ and the product ion *m/z* 329.0657 [M–H–238]^−^ by the loss of the guaiacylacetylglyceryl group. HRMS data and retention time for peak 44 were identical to those of the standard compound, tricin 4'-*O*-[*threo*-*β*-guaiacyl-(9"-*O*-acetyl)-glyceryl] ether. Thus, peaks 43 and 44 were tentatively identified as tricin 4'-*O*-[*erythro*-*β*-guaiacyl-(9"-*O*-acetyl)-glyceryl] ether (60) and tricin 4'-*O*-[*threo*-*β*-guaiacyl-(9"-*O*-acetyl)-glyceryl] ether (60), respectively. Standard compound, tricin 4'-*O*-[*threo*-*β*-guaiacyl-(9"-*O*-acetyl)-glyceryl] ether exhibited a CCS value of 234.58 Å^2^ and did not show other fragment ions except for one at *m/z* 329.07217 (Supplementary Figure S63).

In the extracted ion chromatogram for *m/z* 581.1650, two main peaks, 49 and 50, were observed (Supplementary Figure S64). Peaks 49 (t_R_ = 11.76 min, mass error − 1.9 ppm) and 50 (t_R_ = 11.90 min, mass error − 1.2 ppm) gave a precursor ion at *m/z* 581.1648 [M − H]^−^ and a product ion at 329.0657 [M–H–252]^−^ by the loss of the guaiacylmethylacetylglyceryl group. Further comparison of the HRMS data of the authentic compounds, tricin 4'-*O*-[*threo*-*β*-guaiacyl-(7"-*O*-methyl-9"-*O*-acetyl)-glyceryl] ether (60) and tricin 4'-*O*-[*erythro*-*β*-guaiacyl-(7"-*O*-methyl-9"-*O*-acetyl)-glyceryl] ether (60) confirmed that peaks 49 and 50 were clearly identified as the respective molecules. CCS values of the *threo*-form and *erythro*-form of this molecule were 245.81 and 239.87 Å^2^, respectively, identifying different diastereomers as the CCS value differs by >2 Å^2^. In the HDMS^E^, other fragment ions except for the fragment ion at *m/z* 329.07217 were not observed (Supplementary Figures S65, S66), unlike the difference in the intensity of the fragment ion seen in HDMS^E^ of compounds corresponding to peaks 39–42.

In addition to comparative identification using standard materials, HRMS and MSMS data of peaks for flavonolignans, 4'-*O*-guaiacylglyceryl tricin, and 4'-*O*-*β*-*p*-hydroxyphenylglyceryl tricin, which have been usually found in rice (*O. sativa* and *Z. latifolia*), rattan palm (*C. quiquesetinervius*), and sugarcane (*Saccharum officinarum*), among others (22), were analyzed as follows. Two main peaks (28 and 31) were shown in the extracted ion chromatogram for *m/z* 495.129 (Supplementary Figure S67). Peaks 28 (t_R_ = 7.00 min, mass error 0.6 ppm) and 31 (t_R_ = 7.43 min, mass error − 0.6 ppm) exhibited the same molecular ion at *m*/*z* 495.1294 [M − H]^−^ and 495.1288 [M − H]^−^, respectively. Their MS/MS spectra also showed the same major fragment ions at *m*/*z* 329.0658 and 329.0663 [M − H − 166]^−^, respectively. Comparison with the literature revealed that this fragment ion corresponded to the loss of the *p*-hydroxyphenylglyceryl group. Therefore, peaks 28 and 31 were tentatively identified as tricin 4'-*O*-(*threo*-*β*-*p*-hydroxyphenylglyceryl) ether (52) and tricin 4'-*O*-(*erythro*-*β*-*p*-hydroxyphenylglyceryl) ether, respectively (52), and they are interchangeable. These compounds have been found in rattan palm (*C. quiquesetinervius*) (52), however, not in wheat (*Triticum* species).

Two main peaks, 29 and 32, appeared in the extracted ion chromatogram for *m/z* 525.1140 (Supplementary Figure S68). Peaks 29 (t_R_ = 7.22 min, mass error 0.4 ppm) and 32 (t_R_ = 7.68 min, mass error − 0.2 ppm) showed the same deprotonated molecular ions at *m*/*z* 525.1399 and 525.1395. The major fragment ions at *m*/*z* 329.0663 and 329.0661 [M − H − 196]^−^ were by the loss of a guaiacylglyceryl group (47). Thus, these peaks were tentatively identified as tricin-4'-*O*-(*threo*-*β*-guaiacylglyceryl) ether and tricin-4'-*O*-(*erythro*-*β*-guaiacylglyceryl) ether, respectively, and they are interchangeable. These compounds have been found in many plants, such as rice (*O. sativa* and *Z. latifolia*), thatching grass (*Hyparrhenia hirta*), vetivergrass (*Vetiveria zizanioides*), and johnsongrass (*Sorghum halepense*) with high isolation yield (47, 53, 54); however, they are not found in wheat (*Triticum* species).

Peak 33 (t_R_ = 8.30 min, mass error − 0.6 ppm) was detected with a deprotonated molecular ion at *m*/*z* 523.1237. The fragmentation ion at *m*/*z* 329.0657 [M − H − 194]^−^ represents the molecular ion that is 2 Da less than that of peaks 29 and 32. These observations suggest that the molecule has a double bond between C-8″ and C-9″ of the guaiacylglyceryl group or the carbonyl group at C-7″. Therefore, peak 33 was tentatively identified as tricin-4'-*O*-[2,3-dihydroxy-3-(4-hydroxy-3-methoxyphenyl)-2-propen-1-ol] ether or tricin-4'-*O*-[2,3-dihydroxy-1-(4-hydroxy-3-methoxyphenyl)-1-propenone] ether. Tricin-4'-*O*-[2,3-dihydroxy-3-(4-hydroxy-3-methoxyphenyl)-2-propen-1-ol] ether has not been described previously. Tricin-4'-*O*-[2,3-dihydroxy-1-(4-hydroxy-3-methoxyphenyl)-1-propenone] ether (= tricin-4'-*O*-(*C*-veratroylglycol) ether) has been found in *Arenaria kansuensis* (56), however, not in wheat (*Triticum* species).

Peak 23 (t_R_ = 5.48 min, mass error − 2.3 ppm) exhibited as a deprotonated ion [M − H]^−^ at *m*/*z* 477.1175, and the MS/MS profile was observed with signals at *m*/*z* 431.0764 [M − H − 46]^−^ by loss of 2(CH_3_) + OH, *m*/*z* 416.0524 [M − H − 46 − 15]^−^ by loss of 3(CH_3_) + OH, and *m*/*z* 329.0653 [M − H − 148]^−^ by the loss of the *p*-hydroxyphenylpropanol group linked by C-3 and C-2′, which has been elucidated as a functional group in the flavonolignan previously reported as natansin A (50). Therefore, peak 23 was tentatively identified as natansin A, which was found in *Triticum* species for the first time.

Peak 26 (t_R_ = 5.81 min, mass error − 0.4 ppm) showed the major molecular ion at *m*/*z* 507.1289 [M − H]^−^ and the fragment ions at *m*/*z* 461.0572 [M − H − 46]^−^ by loss of 2(CH_3_) + OH, *m*/*z* 446.0638 [M − H − 46 − 15]^−^ by loss of 3(CH_3_) + OH, consistent with the fragment pattern of peak 23, and the fragment ion at *m*/*z* 329.0653 [M − H − 178]^−^ by the loss of the guaiacylpropanol group linked by C-3 and C-2′, which has been elucidated as a functional group in the flavonolignan previously discovered by Wenzig et al. (51). Based on this information, peak 26 was tentatively identified as 5,6-dihydro-3,8,10-trihydroxy-5-(4-hydroxy-3-methoxyphenyl)-6-hydroxymethyl-2,4-dimethoxy-7*H*-benzo[c]xanthen-7-one, which was found in *Triticum* species for the first time.

### 3.4 Flavonolignan-glycosides (tricin-lignan-glycoside derivatives)

Tricin-lignan-glycosides have been found in a few plants, such as rice plants (*O. sativa* and *Z. latifolia*), alfalfa (*M. sativa*), and sugarcane (*S. officinarum*) (22). Tricin-lignan-glycosides have a structure in which the lignan (phenylpropanoid) group is usually located at the 4'-OH of tricin, with a sugar group linked to the 7-OH of tricin or the lignan group. In the MS/MS analysis of tricin-lignan-glycosides, characteristic fragment ions by the loss of guaiacylglyceryl ether (196 Da), hexoside (162 Da), or lignanglycosides were observed.

In the extracted ion chromatogram for *m/z* 687.193, eight peaks, 10, 13, 15, 17, 19, 21, 22, and 25, were observed (Supplementary Figure S69). Peak 10 (t_R_ = 3.59 min, mass error − 2.3 ppm) possessed a major molecular ion [M − H]^−^ at *m*/*z* 687.1917 and two major fragment ions were observed at *m*/*z* 491.1172 [M − H − 196]^−^ corresponding to tricin-*O*-glycoside due to loss of the guaiacylglyceryl group and at *m*/*z* 329.2835 [M − H − 196 − 162]^−^ by the loss of glucoside from the molecule of tricin-*O*-glycoside. In addition, since peak 10 was the least abundant among the extracted peaks, it was assumed that *O*-glucoside was located at the 5-OH rather than 7-OH, which is mainly subjected to glycosylation. Therefore, peak 10 was tentatively identified as tricin-4'-*O*-(*β*-guaiacylglyceryl) ether 5-*O*-glucoside, which has not been described previously.

Peaks 13 (t_R_ = 4.03 min, mass error − 0.7 ppm) and 15 (t_R_ = 4.45 min, mass error − 1.3 ppm) presented major molecular ions at *m*/*z* 687.1920 [M − H]^−^, and the MS/MS ions at *m*/*z* 525.1398 [M − H − 162]^−^ by the loss of glucoside, 491.1172 [M − 196]^−^ corresponding to tricin-*O*-glycoside due to loss of the guaiacylglyceryl group, and 329.0653 [M − H − 162 − 196]^−^ by the loss of the glucose and guaiacylglyceried groups. Therefore, these peaks were tentatively identified as tricin-4'-*O*-(*threo*-*β*-guaiacylglyceryl) ether 7-*O*-glucoside and tricin-4'-*O*-(*erythro*-*β*-guaiacylglyceryl) ether 7-*O*-glucoside, and they are interchangeable. These compounds have been isolated from rice (*O. sativa* and *Z. latifolia*), thatching grass (*H. hirta*), and bamboo (*Neosinocalamus affinis*) (22, 47, 48); however, they were found in *Triticum* species for the first time.

The precursor ions and fragmentation patterns of peaks 17 (t_R_ = 4.82 min, mass error − 1.2 ppm) and 19 (t_R_ = 5.19 min, mass error 1.0 ppm) were similar to those of peaks 13 and 15; however, the fragment ion at *m/z* 491 [M − H − 196]^−^ was not shown for tricin-*O*-glycoside resulting from the guaiacylglyceryl group cleavage, which was observed in the MS/MS data of peaks 10, 13, and 15, indicating that *O*-glucoside was positioned at -OH group of glyceryl group. Therefore, peaks 17 and 19 were tentatively identified as tricin 4'-*O*-(*threo*-*β*-guaiacylglyceryl) ether 7"-*O*-*β*-glucoside and tricin 4'-*O*-(*erythro*-*β*-guaiacylglyceryl) ether 7"-*O*-*β*-glucoside, respectively, by comparing the MS/MS data of each single compounds reported in the literature (49), but they are interchangeable. These compounds have been found in *Triticum* species for the first time.

Peak 21 (t_R_ = 5.31 min, mass error − 1.2 ppm) exhibited identical major molecular ions at *m*/*z* 687.1917 [M − H]^−^; however, the only fragment ion was shown at *m*/*z* 329.0654 [M − H − 162 − 196]^−^ by the loss of the glucose and guaiacylglyceried groups. Therefore, this peak was tentatively identified as tricin 4'-*O*-(*β*-guaiacylglyceryl) ether 9"-*O*-*β*-D-glucoside, which has been previously found in rice (*O. sativa*) (22); however, it has not been reported in *Triticum* species.

Peaks 22 (t_R_ = 5.44 min, mass error 0.6 ppm) and 25 (t_R_ = 5.79 min, mass error − 0.2 ppm) showed an identical major deprotonated molecular ion at *m*/*z* 687.1929; however, the fragment ions at *m*/*z* 525.1398 [M − H − 162]^−^ by the loss of glucoside and at *m*/*z* 491 [M − H − 196]^−^ corresponding to tricin-*O*-glycoside were not observed, suggesting that these are not a glycoside (presenting the absence of sugar group). Peak 22 produced the fragment ion at *m*/*z* 477.1285 [M − H − 210]^−^ by the loss of the guaiacylmethylglyceryl group and *m*/*z* 329.0654 [M − H − 210 − 148]^−^ by loss of another *p*-hydroxyphenylpropanol group linked by C-3 and C-2′, as shown by the molecular ions corresponding to 210 Da and 148 Da found from the fragment ion patterns of peaks 41 and 23, respectively, mentioned in above (section 3.3). Therefore, peak 22 was tentatively identified as natasin A 4'-*O*-[*β*-guaiacyl-(7"-*O*-methyl)-glyceryl] ether, which has not been described previously. Peak 25 showed the fragment ion at *m*/*z* 507.1295 [M − H − 180]^−^ by the loss of the guaiacylmethylglyceryl group and *m*/*z* 328.0587 [M − H − 180 − 178]^−^ by loss of another guaiacylpropanol group linked by C-3 and C-2′, which were observed as molecular ions of 180 Da and 178 Da found from the fragment ion patterns of peaks 38 and 25, respectively (section 3.3). Thus, peak 25 was tentatively identified as tricin-2′,3-dihydropropanol-2′-(4-hydroxy-3-methoxyphenyl) 4'-*O*-[*β*-guaiacyl-(7"-*O*-methyl)-glyceryl] ether, which has not been described previously.

In the extracted ion chromatogram for *m/z* 657.182, six peaks, 12, 14, 16, 18, 20, and 24 were observed (Supplementary Figure S70). Peaks 12 (t_R_ = 3.85 min, mass error − 2.3 ppm) and 14 (t_R_ = 4.23 min, mass error − 3.8 ppm) gave a precursor ion at *m/z* 657.1804 [M − H]^−^ and a product ion at *m/z* 495.1280 [M–H–162]^−^ by the loss of glucoside and *m/z* 329.0659 [M–H–162–166]^−^ by the loss of *p*-hydroxyphenylglyceryl. Therefore, these peaks were tentatively identified as tricin 4'-*O*-(*threo*-*β*-*p*-hydroxyphenylglyceryl) ether 7-*O*-glucoside and tricin 4'-*O*-(*erythro*-*β*-*p*-hydroxyphenylglyceryl) ether 7-*O*-glucoside, and they are interchangeable. These two molecules have not been reported so far.

Peaks 16 (t_R_ = 4.80 min, mass error − 0.5 ppm), 18 (t_R_ = 5.15 min, mass error − 0.8 ppm), and 20 (t_R_ = 5.24 min, mass error − 0.5 ppm) showed similar precursor ions to those of peaks 12 and 14. However, only a fragment ion at *m/z* 329.0653 [M − H − 162 − 166]^−^ was observed, presenting the loss of the glucose and *p*-hydroxyphenylglyceryl group. Therefore, these peaks were tentatively identified as any of the three molecules among the four structures: tricin 4'-*O*-(*threo*-*β*-*p*-hydroxyphenylglyceryl) ether 7"-O- glucoside, tricin 4'-*O*-(*erythro*-*p*-hydroxyphenylglyceryl) ether 7"-*O*-glucoside, tricin 4'-*O*-(*threo*-*β*-*p*-hydroxyphenylglyceryl) ether 9"-*O*-glucoside, and tricin 4'-*O*-(*erythro*-*β*-*p*-hydroxyphenylglyceryl) ether 9"-*O*-glucoside, which have not been described yet.

Peak 24 (t_R_ = 5.60 min, mass error 0.3 ppm) exhibited identical precursor ions at *m*/*z* 657.1821 [M − H]^−^ and yielded the product ions at *m*/*z* 477.11181 [M − H − 180]^−^ by the loss of the *p*-hydroxyphenylmethylglyceryl group and *m*/*z* 329.0654 [M − H − 210 − 148]^−^ by loss of another *p*-hydroxyphenylpropanol group linked by C-3 and C-2′, of which 180 Da and 148 Da were found from the fragment ion patterns of peaks 39 and 23, respectively, mentioned above (section 3.3). Therefore, peak 24 was tentatively identified as natasin A 4'-*O*-[*β*-*p*-hydroxyphenyl-(7"-O-methyl)-glyceryl] ether, which has not been described previously.

### 3.5 Others

The chemical profiles of fatty acids and glycerophosphocholines from whole wheat bread have been obtained using LC–MS (54). Their molecular structures have been identified through a comparison of their accurate mass confirmation and MS/MS fragmentation by LC-QTOF MS analysis with the standards (54). Based on the MS information of these compounds, the molecular structures of the same complex peaks in the non-polar range in the BPI chromatograms of wheat hull samples were analyzed. In the extracted ion chromatogram for *m/z* 329.233 [M − H]^−^ for pinellic acid (57), a number of clumped peaks (peaks 34–38 and 45–48) were observed (Supplementary Figure S71).

Among them, the peaks 34–37 showed major molecular ions at *m*/*z* 329.2330 [M − H]^−^ and the fragment ions at *m*/*z* 229.1432 [M − C_6_H_13_O]^−^ and *m*/*z* 211.1332 [M − C_6_H_13_O–H_2_O]^−^, identical to the MS/MS fragment pattern of pinellic acid (9,12,13-trihydroxy-*trans*-10-octadecenoic acid), which was reported in whole wheat bread (54). Therefore, the peaks 34–37 were tentatively identified as pinellic acid isomers. The peak 38 (t_R_ = 8.92 min, mass error − 0.3 ppm) exhibited major molecular ions at *m*/*z* 329.2327 [M − H]^−^; however, the different fragment ions were observed at *m*/*z* 243.8795 [M–C_5_H_9_ − H_2_O]^−^ and *m*/*z* 171.1021 [M–C_9_H_17_O − H_2_O]^−^, suggesting that this peak was identified as 5,8,12-trihydroxy-*trans*-9-octadecenoic acid, which has been isolated from wheat bran (58).

Peaks 45–48 showed the same major molecular ions at *m*/*z* 329.2330 [M − H]^−^ as peaks 34–38 but yielded different product ion patterns. Thus, these peaks were tentatively identified as trihydroxy octadecenoic acid isomers.

In the extracted ion chromatogram for *m/z* 313.2379 [M − H]^−^ for 12,13-dihydroxy-9*Z*-octadecenoic acid (54), peak 51 (t_R_ = 13.96 min, mass error 1.0 ppm) showed a major molecular ion at *m*/*z* 313.2376 [M − H]^−^ and the fragment ions at *m*/*z* 295.2268 [M–H_2_O]^−^ and *m*/*z* 183.1383 [M − H_2_O–C_7_H_13_O]^−^, identical to the MS/MS fragment pattern of 12,13-dihydroxy-9*Z*-octadecenoic acid, which was reported in whole wheat bread (57).

In the extracted ion chromatogram for *m/z* 564.3313 [M + FA − H]^−^ for 1-(9*Z*,12*Z*-octadecadienoyl)-*sn*-glycero-3-phosphocholine (57), the precursor ions and fragmentation patterns of peak 55 (t_R_ = 17.81 min, mass error 0.7 ppm) was identical to those of 1-(9*Z*,12*Z*-octadecadienoyl)-*sn*-glycero-3-phosphocholine, exhibiting the major molecular ions at *m*/*z* 564.3305 [M + FA − H]^−^ and fragment ions at *m/z* 279.2333 [M − H − C_8_H_19_NO_5_P]^−^ and *m/z* 224.0687 [M − H − CH_3_ − C_18_H_31_O_2_]^−^.

Peaks 52 and 55 showed the identical adduct ion and fragment pattern of peak 54. Peak 52 (t_R_ = 16.15 min, mass error − 1.8 ppm) was detected with a precursor molecular ion at *m*/*z* 562.3135 [M + FA − H]^−^ and the product ion at *m*/*z* 277.2168 [M + FA − H − C_8_H_19_NO_5_P]^−^, representing the molecular ion that is 2 Da less than that of peak 54. A fragment ion at *m/z* 224.0687 [M − H − CH_3_ − C_18_H_31_O_2_]^−^ was also observed. Therefore, this peak was tentatively identified as 1-(9*Z*,12*Z*,15*Z*-octadecatrienoyl)-*sn*-glycero-3-phosphocholine, which has not been found in wheat (*Triticum* species). Peak 55 (t_R_ = 19.81 min, mass error − 0.4 ppm) exhibited a major molecular ion at *m*/*z* 566.3456 [M + FA − H]^−^ with a molecular weight of 2 Da more than that of peak 54. The fragment ions were observed at *m*/*z* 339.1991 [M + FA − H − C_5_H_14_NO_4_P]^−^ and *m*/*z* 281.2479 [M + FA − H − C_8_H_19_NO_5_P]^−^. Therefore, this peak was tentatively identified as 1-(9*Z*-octadecenoyl)-*sn*-glycero-3-phosphocholine, which has not been found in wheat (*Triticum* species).

In the extracted ion chromatogram for *m/z* 295.2273 [M − H]^−^ for 10*E*,12*Z*-9-hydroxyoctadecadienoic acid (9-HODE) or 9*Z*,11*E*-13-hydroxyoctadecadienoic acid (13-HODE) (57), peak 53 (t_R_ = 17.61 min, mass error − 1.0 ppm) exhibited an identical precursor ion at *m/z* 295.2276 [M − H]^−^ and the product ions at *m/z* 277.2167 [M − H_2_O]^−^ and *m/z* 195.1380 [M − H_2_O − C_6_H_11_]^−^, similar to those of 9*E*,11*E*-13-hydroxyoctadecadienoic acid (13-HODE). Therefore, peak 53 was tentatively identified as 9*E*,11*E*-13-hydroxyoctadecadienoic acid (13-HODE) reported in the literature (57).

### 3.6 Biological activity of wheat hull samples

**Figure 2:**
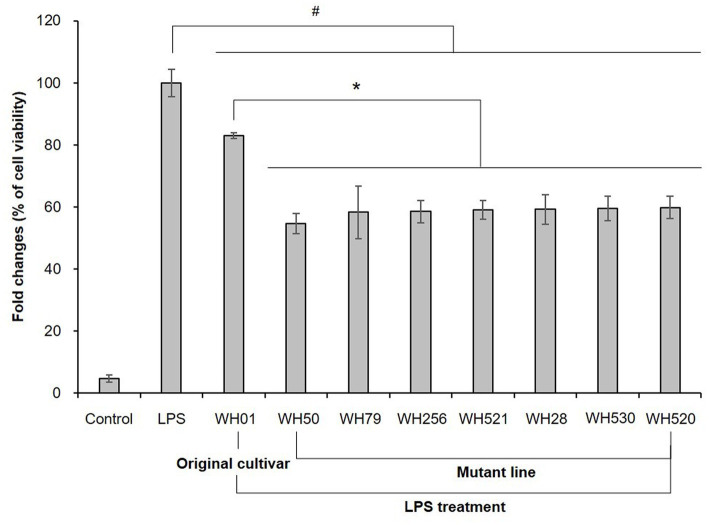
The effects of the original cultivars and selected mutant lines on NO production in LPS-stimulated RAW 264.7 cells. The values are expressed as the mean ± SD of three independent experiments. ^#^*p* < 0.05 versus LPS only treated group. ^*^p < 0.05 versus original cultivar.

All wheat hull extracts (50 μg/mL) were evaluated for nitric oxide (NO) production inhibitions and cell viabilities in lipopolysaccharide (LPS)-stimulated RAW 264.7 macrophage cells (Supplementary Table S1). The two samples of the original cultivar (WH1 and WH2) showed NO production inhibition activity with 16.92 ± 0.92% and 19.42 ± 3.28%, respectively. Among 983 mutant lines, 686 mutant lines exhibited greater inhibitory activity than the original cultivar; however, they either showed similar activity or there was no significant difference between them. Thus, only mutant lines with an inhibition rate of ≥40% were designated as an active group, followed by WH50, WH79, WH256, WH521, WH28, WH530, and WH520 (Figure 2). The LPS treatment markedly increased NO production in RAW 264.7 cells compared with the control cells, whereas the pretreatment of WH50, WH79, WH256, WH521, WH28, WH530, and WH520 inhibited NO production. All samples exhibited no obvious cytotoxicity at 50 μg/mL. In our previous study, phytochemical investigation of the ethyl acetate-soluble fraction of wheat hull, which inhibited LPS-induced NO production with 62% at 50 μg/mL, led to the isolation and identification of a flavone, tricin, and seven flavonolignans. Tricin 4'-O-[*erythro*-*β*-guaiacyl-(9"-*O*-acetyl)-glyceryl] ether exhibited their IC_50_ value of 24.14 μM, indicating greater inhibitory activity than the positive control, N^G^-monomethyl-L-arginine acetate salt (51.01 μM). Additionally, it has been reported that wheat hull is a rich source of tricin, which showed selective cytotoxicity against two cancer cell lines, hepatic HepG2 and pancreatic IN383/12, but no effect on normal cells NIH3T3 (11). Our study reports for the first time the anti-inflammatory evaluation and comparison of the extracts of collected hulls from the original wheat variety and its radiation-bed wheat lines. Therefore, wheat mutant lines with excellent anti-inflammatory activity may contribute to the higher valorization of wheat by-products, although further efficacy verification and standardization studies are needed.

### 3.7 Multivariate analysis

**Figure 3:**
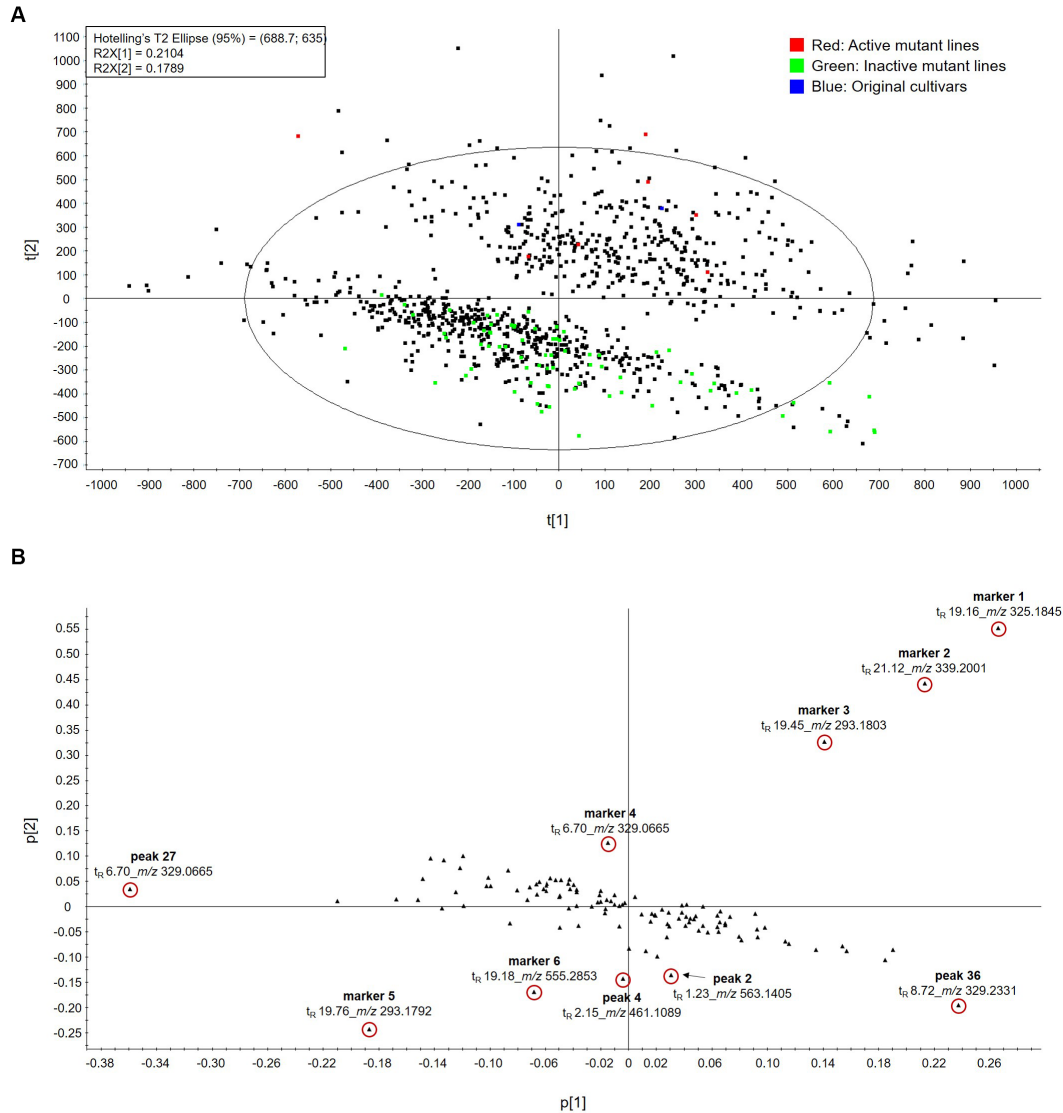
Principal component analysis (PCA) score plot **(A)** and loading plot **(B)** of metabolome analysis of the 983 wheat hull samples.

Recently, multivariate statistical analysis has been shown to be an effective approach for comparing differences between experimental setups in untargeted and targeted metabolomic studies. The metabolite profiles of the wheat original cultivar and its 983 mutant lines, which were grown under the same environmental conditions, were analyzed via UPLC-QTOF MS. It was difficult to find significant differences between the BPI chromatograms of the mutant lines; thus, we performed PCA, orthogonal partial least squares-discriminant analysis (OPLS-DA), S-plots, and VIP plot, all of which have been widely used in recent years for the metabolomic analysis of extremely complex samples. The 985 samples, including two original cultivars, were clustered into two groups by the PCA scores (Figure 3A). The first two principal components accounted for 85.0% of the total variance, with 21.0 and 17.9% by PC1 and PC2, respectively. Upon detailed examination of the samples belonging to the two stratified groups, group I contained wheat hull extract that inhibited LPS-induced NO production by more than 40%, and group II contained samples, almost all of which were inactive. The corresponding PCA loading plot (Figure 3B; Supplementary Figure S72) demonstrated that 10 markers were responsible for group segregation. However, since multivariate statistical analysis was processed, including all molecular ions detected in the BPI chromatogram, molecular ions that were not identified in the above in-house library appeared to be an important variable. Therefore, the most valid molecular formula for their HRMS ion values was obtained from MassLynx (Waters Corporation, Milford, MA, United States), which collects exact mass and exact MS/MS data, and molecular structure prediction was attempted using databases of GNPS (36), PubMed (68), and ChemSpider (69). In the PCA loading plot, the four markers located away from the center in the same direction as group I displayed their molecular ions ([M – H]^−^) at *m/z* 325.1845 (t_R_ = 19.16 min; marker 1), *m/z* 339.2001 (t_R_ = 21.12 min; marker 2), *m/z* 293.1803 (t_R_ = 19.45 min; marker 3), and *m/z* 265.1478 (t_R_ = 15.70 min; marker 4). Marker 1 exhibited its retention time at 19.13 min in the BPI chromatogram and a major molecular ion at *m*/*z* 325.1838 [M − H]^−^ in its HRMS, which corresponded to an elemental formula of C_21_H_26_O_3_ (Supplementary Figures S73, S74). Thus, this peak was predicted to be heptaethylene glycol as the most relevant structure searched in the database of PubMed (68) and ChemSpider (69); however, its mass error was calculated to be 10.5 ppm. Marker 2 showed a major molecular ion at *m*/*z* 339.1989 [M − H]^−^, which is 14 Da more than that of marker 1, and a molecular formula C_22_H_28_O_3_ with an added CH_2_ group (Supplementary Figures S75, S76). This molecular formula corresponded to heptaethylene glycol monomethyl ether, the most relevant searched in PubMed (68) and ChemSpider (69) databases. Marker 3 was predicted using the GNPS database (36). GNPS is an open-access community-curated analysis platform for sharing natural product MS data, including raw, processed, or annotated fragmentation MS data (MS/MS) (41). Raw LC–MS/MS data for the wheat hull sample (WH50) was submitted to the GNPS platform and searched for the molecules by comparing it with the MS/MS data of natural products in the GNPS database; however, only a small number of molecules were found (Supplementary Figure S77). Among the 13 molecules found in the database of GNPS, 6-gingerol was found, which showed a similar HRMS ion (*m*/*z* 293.1795 [M − H]^−^) as marker 3 (t_R_ = 19.30 min), which was detected with a major molecular ion at *m*/*z* 293.1795 [M − H]^−^ (Supplementary Figure S78). Thus, marker 3 was tentatively identified as 6-gingerol, but its mass error of 14.3 ppm was calculated. 6-Gingerol was reported in stilbenoid, diarylheptanoid, and gingerol biosynthesis of *Aegilops tauschii* (the diploid progenitor of the D genome of hexaploid wheat (*T. aestivum*) and rice (*O. sativa*) in Kyoto Encyclopedia of Genes and Genomes (KEGG) pathway database) (70). In addition, 6-gingerol has been detected in metabolite analysis of plants from Poaceae, including wheat (*T. aestivum*) (71). Marker 4 showed a precursor molecular ion at *m*/*z* 265.1470 [M − H]^−^, representing the molecular ion that is 28 Da less than that of marker 3 (Supplementary Figure S79). Therefore, this peak was tentatively identified as 4-gingerol (mass error 12.1 ppm), where two methylene (CH_2_) groups were removed from the alkane chain of 6-gingerol. This compound has not been found in wheat (*Triticum* species). Marker 5 exhibited its retention time at 19.57 min in the BPI chromatogram and was found to have the molecular formula C_17_H_26_O_4_, corresponding to its molecular ion at *m*/*z* 293.1784 [M − H]^−^ (Supplementary Figures S80, S81). This molecular formula was tentatively identified as phytuberin, best matched in the database of PubMed (68). However, its mass error was calculated to be 10.6 ppm. Phytuberin has been found in the LC-HRMS-based metabolite profile of wheat flag leaves (*T. aestivum*) (72). Marker 6 was found to have the molecular formula C_28_H_44_O_11_ by the HRMS data (*m*/*z* 555.2839 [M − H]^−^) (Supplementary Figures S82, S83). This molecular formula corresponded to several structures among the compounds searched in the PubMed and ChemSpider databases, excluding glycosides. Thus, the molecule for this peak was not predicted. Table 3 lists the t_R_ values, calculated and observed deprotonated molecular ions *m*/*z* [M − H]^−^, calculated error ppm, and proposed molecular formula of markers 1–6. However, because the mass errors between markers 1–6 and their tentatively identified molecular structures are too large, there are limits to the proposed molecular structures, and thus, further comparison with their standards is necessary.

**Table 3:** Characterization and tentative identification of markers 1–6 using UPLC-ESI QTOF MS in negative mode.

Marker No.	t_R_ (min)	Observed mass (*m*/*z*)	Calculated Mass (*m*/*z*)	Error (ppm)	Molecular formula	Identification^1^
1	19.13	325.1838	325.1804	10.5	C_21_H_26_O_3_	Heptaethylene glycol
2	21.10	339.1989	339.1960	8.5	C_22_H_28_O_3_	Heptaethylene glycol monomethyl ether
3	19.30	293.1795	293.1753	14.3	C_17_H_26_O_4_	6-Gingerol
4	15.40	265.1470	265.1440	11.3	C_15_H_22_O_4_	4-Gingerol
5	19.57	293.1784	293.1753	10.6	C_17_H_26_O_4_	Phytuberin
6	18.93	555.2839	555.2805	6.1	C_28_H_44_O_11_	Unkown

^1^Molecular structure prediction was further attempted using databases GNPS (41), PubMed (68), and ChemSpider (69).

**Figure 4:**
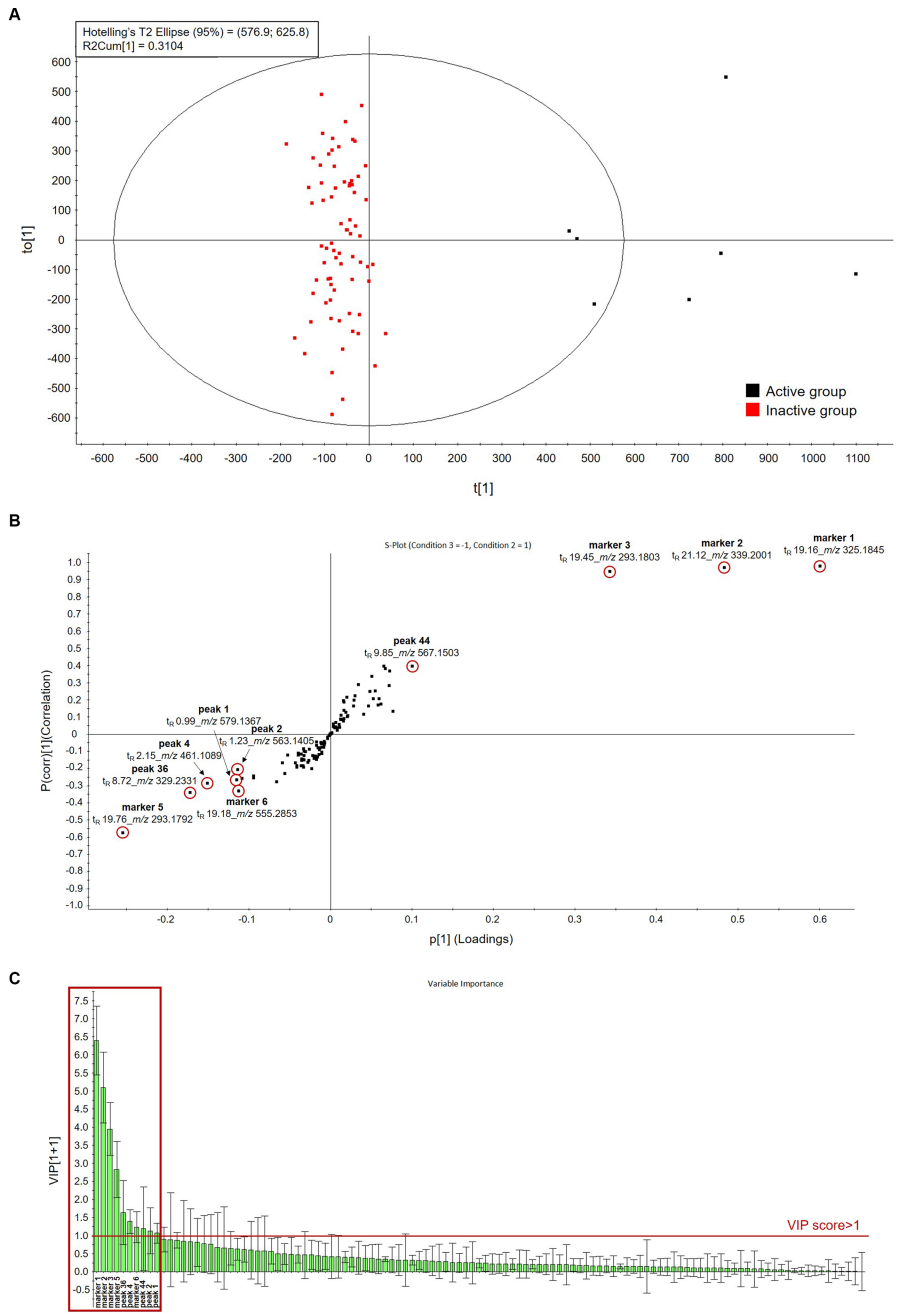
Orthogonal partial least-squares discriminant analysis (OPLS-DA) score plot **(A)**, S-plot **(B)**, and importance in projection (VIP) plot **(C)** between active and inactive groups.

PCA is capable of identifying overall variability directions, whereas OPLS-DA is capable of distinguishing variabilities among groups. Therefore, OPLS-DA was performed after designating the top seven samples that inhibited NO production >40% as the active group and the bottom 73 samples that did not show inhibition of NO production as the inactive group. The OPLS-DA score plot showed a clear separation of the clusters when applying multivariate analysis to the normalized dataset (Figure 4A). The OPLS-DA model quality can be estimated using the cross-validation parameters Q2 (model predictability) and R2(y) (total explained variation for the X matrix). OPLS-DA of the samples produced one predictive and one orthogonal (1 + 3) component and showed that the cross-validated predictive ability Q2 was 0.84, and the variance related to the differences between the two origins R2(y) was 0.88. In most cases, a Q2 value greater than 0.5 is adequate, and the difference between R2 and Q2 values should be less than 0.3. The S-plot (Point, t_R_-*m*/*z* pair) from the OPLS-DA model, which is a useful tool for comparing the magnitude and reliability of a variable, was also analyzed. The markers associated with the 80 samples were examined based on the distribution on the S-plot (Figure 4B; Supplementary Figure S84). Among the markers with the threshold of VIP value (VIP > 1.0) (Figure 4C; Supplementary Figure S85), four markers shown by their molecular ions ([M – H]^−^) at *m/z* 325.1845 (t_R_ = 19.16 min; marker 1), *m/z* 339.2001 (t_R_ = 21.12 min; marker 2), *m/z* 293.1803 (t_R_ = 19.45 min; marker 3), and *m/z* 567.1503 (t_R_ = 9.85 min; tricin 4'-O-[*erythro*-*β*-guaiacyl-(9"-*O*-acetyl)-glyceryl] ether; peak 44) were located in the same direction as the active group in the S-plot and were therefore suggested as distinguishable markers for the active group which can be attributed to activity. Differential markers in the same direction as the inactive group demonstrated their molecular ions ([M − H]^−^) at *m/z* 293.1792 (t_R_ = 19.76 min; marker 5), *m/z* 329.2331 (t_R_ = 8.72 min; pinellic acid isomer; peak 36), *m/z* 461.1089 (t_R_ = 2.15 min; chrysoeriol-8-*C*-glucoside; peak 4), *m/z* 555.2853 (t_R_ = 19.18 min; C_28_H_44_O_11_; marker 6), *m/z* 563.1405 (t_R_ = 1.23 min; isochaftoside; peak 2), and *m/z* 579.1367 (t_R_ = 0.99 min; isocarlinoside; peak 1) and can be proposed as differential components of the inactive group.

Of the four components attributable to the activity, 6-gingerol is a potent anti-inflammatory agent. 6-Gingerol exerted its anti-inflammatory effect in LPS-induced macrophages by inhibiting the production of NO, tumor necrosis factor-α, interleukin (IL)-1β, IL-6, and prostaglandin E2 and reducing the expression of inducible nitric oxide synthase and cyclooxygenase-2 via the inhibition of the nuclear factor-κB signaling pathways (73). In addition, 6-gingerol suppressed the secretion of pro-inflammatory cytokines and inhibited macrophage cell pyroptosis by targeting mitogen-activated protein kinase signaling pathways, resulting in overall inhibition of sepsis development (74). In addition, related to another component, tricin 4'-O-[*erythro*-*β*-guaiacyl-(9"-*O*-acetyl)-glyceryl] ether, flavonolignans which were isolated from the hull of *T. aestivum* have been evaluated for their inhibitory effect of NO production in LPS-stimulated RAW 264.7 macrophage cells in our previous study, and tricin 4'-O-[*threo*-*β*-guaiacyl-(9"-*O*-acetyl)-glyceryl] ether showed the greatest effect (17). Thus, its diastereomer, tricin 4'-O-[*erythro*-*β*-guaiacyl-(9"-*O*-acetyl)-glyceryl] ether can also be expected to have an anti-inflammatory effect. Although the biological action of heptaeylene glycol is not clear, it was discovered to be a differential metabolite in patients with hepatocellular carcinoma compared to healthy controls with downregulated plasma levels (75). Moreover, heptaeylene glycol inhibited the proliferation, migration, and invasion of hepatocellular carcinoma cells (HuH-7 and SNU-449) (70). Thus, it can be postulated that heptaethylene glycol monomethyl ether may also have an anticancer activity with its other biological activities.

## 4 Conclusion

The chemical compositions of wheat hull samples of the original cultivars (WH01 and WH02) and its 983 mutant lines were analyzed by UPLC-QTOF MS, and a total of 55 molecules were tentatively identified, including 21 compounds found in the *Triticum* species for the first time and 13 compounds not previously described. In addition, the results of distinguishing flavonolignan diastereomers using the calculation of their CCS values were derived for the first time in this study, and these results serve as a reference for further identification of the isomeric properties of tricin-lignan isomers. OPLS-DA based on the results of untargeted metabolite analysis of 985 wheat hull samples and their LPS-induced NO production measurements led to the proposal of 10 markers that contributed a distinction between the different groups of the mutant lines, the active group containing seven samples with NO production inhibitory effect >40%, and the inactive group containing 73 samples with the lowest effect. Four metabolites tentatively identified, heptaethylene glycol, heptaethylene glycol monomethyl ether, 6-gingerol, and tricin 4'-O-[*erythro*-*β*-guaiacyl-(9"-*O*-acetyl)-glyceryl] ether, were selected as key bioactive markers that could contribute to characteristic anti-inflammatory mutant lines. Therefore, these results suggested that wheat hull samples from the selected mutant lines and marker compounds can be excellent sources of therapeutic agents for treating or preventing conditions related to inflammatory diseases. In addition, these results will serve as a reference for future investigation of the mutation mechanism of γ-irradiated wheat mutant lines and quality assessment for improved mutation selection.

## Data availability statement

The original contributions presented in the study are included in the article/Supplementary material, further inquiries can be directed to the corresponding authors.

## Ethics statement

Ethical approval was not required for the studies on animals in accordance with the local legislation and institutional requirements because only commercially available established cell lines were used.

## Author contributions

JP: Data curation, Formal analysis, Investigation, Methodology, Writing – original draft. Y-SK: Data curation, Formal analysis, Methodology, Writing – original draft. G-HR: Data curation, Formal analysis, Investigation, Writing – review & editing. CHJ: Data curation, Formal analysis, Investigation, Writing – review & editing. MJH: Resources, Writing – review & editing. J-BK: Resources, Writing – review & editing. C-HJ: Formal analysis, Methodology, Validation, Writing – review & editing. J-WN: Conceptualization, Methodology, Supervision, Validation, Writing – review & editing. A-RH: Conceptualization, Funding acquisition, Methodology, Project administration, Supervision, Writing – original draft, Writing – review & editing.
