# Supplementary material for: Phytochemical profile and anti-inflammatory activity of the hull of γ-irradiated wheat mutant lines (Triticum aestivum L.)
Source: Front Nutr. 2023 Dec 22;10:1334344. doi: 10.3389/fnut.2023.1334344 (PMC10771830; doi:10.3389/fnut.2023.1334344)
Supplement: Supplementary file 1 [file Data_Sheet_1.docx]

Supplementary Material

**Phytochemical Profile and Anti-Inflammatory Activity of the Hull of γ-Irradiated Wheat Mutant Lines (*Triticum aestivum* L.)**

Jisu Park ^1,†^, Yun-Seo Kil ^2,†^, Ga-Hee Ryoo ^1^, Chang Hyun Jin ^1^, Min Jeong Hong ^1^, Jin-Baek Kim ^1^, Joo-Won Nam ^2,^* and Ah-Reum Han ^1,^*

^1^Advanced Radiation Technology Institute, Korea Atomic Energy Research Institute, Jeongeup-si, Jeollabuk-do 56212, Republic of Korea

^2^College of Pharmacy, Yeungnam University, Gyeongsan-si, Gyeongsangbuk-do 38541, Republic of Korea

*** Correspondence:**Corresponding Author
[arhan@kaeri.re.kr](mailto:arhan@kaeri.re.kr) (A.-R.H.); [jwnam@yu.ac.kr](mailto:jwnam@yu.ac.kr) (J.-W.N.); Tel.: +82-63-570-3167 (A.-R.H.); Tel.: +82-53-810-2818 (J.-W.N.)

**^†^**These authors contributed equally to this work

**Table of Contents**

|  |  | Page |
| --- | --- | --- |
| **Figure 1.** | ESI-QTof-MS spectrum of luteolin-6-*C*-arabinoside-8-*C*-glucoside (peak 1) | 6 |
| **Figure 2.** | ESI-QTof-MS spectrum of apigenin-6-*C*-arabinoside-8-*C-*glucoside (peak 2) | 7 |
| **Figure 3.** | ESI-QTof-MS spectrum of apigenin-8-*C*-glucoside-2"-*O*-glucoside (peak 3) | 8 |
| **Figure 4.** | ESI-QTof-MS spectrum of chrysoeriol-8-*C*-glucoside (peak 4) | 9 |
| **Figure 5.** | ESI-QTof-MS spectrum of tricin-7-*O*-sophoroside (peak 5) | 10 |
| **Figure 6.** | ESI-QTof-MS spectrum of chrysoeriol-8-*C*-galactoside (peak 6) | 11 |
| **Figure 7.** | ESI-QTof-MS spectrum of tricin-5-*O*-glucoside (peak 7) | 12 |
| **Figure 8.** | ESI-QTof-MS spectrum of tricin-7-*O*-rutinoside (peak 8) | 13 |
| **Figure 9.** | ESI-QTof-MS spectrum of tricin-7-*O*-glucoside (peak 9) | 14 |
| **Figure 10.** | ESI-QTof-MS spectrum of tricin-4'-*O*-(*β*-guaiacylglyceryl) ether 5-*O*-glucoside (peak 10) | 15 |
| **Figure 11.** | ESI-QTof-MS spectrum of tricin-4'-*O*-glucoside (peak 11) | 16 |
| **Figure 12.** | ESI-QTof-MS spectrum of tricin-4'-*O*-(*threo*-*β*-*p*-hydroxyphenylglyceryl) ether 7-*O*-glucopyranoside (peak 12) | 17 |
| **Figure 13.** | ESI-QTof-MS spectrum of tricin-4'-*O*-(*threo*-*β*-guaiacylglyceryl) ether 7-*O*-glucoside (peak 13) | 18 |
| **Figure 14.** | ESI-QTof-MS spectrum of tricin-4'-*O*-(*erythro*-*β*-*p*-hydroxyphenylglyceryl) ether 7-*O*-glucopyranoside (peak 14) | 19 |
| **Figure 15.** | ESI-QTof-MS spectrum of tricin-4'-*O*-(*erythro*-*β*-guaiacylglyceryl) ether 7-*O*-glucopyranoside (peak 15) | 20 |
| **Figure 16.** | ESI-QTof-MS spectrum of tricin-4'-*O*-(*threo*-*β*-*p*-hydroxyphenylglyceryl) ether 7"-*O*-glucopyranoside (peak 16) | 21 |
| **Figure 17.** | ESI-QTof-MS spectrum of tricin-4'-*O*-(*threo*-*β*-guaiacylglyceryl) ether 7"-*O*-glucopyranoside (peak 17) | 22 |
| **Figure 18.** | ESI-QTof-MS spectrum of tricin-4'-*O*-(*erythro*-*β*-*p*-hydroxyphenylglyceryl) ether 7"-*O*-glucopyranoside (peak 18) | 23 |
| **Figure 19.** | ESI-QTof-MS spectrum of tricin-4'-*O*-(*erythro*-*β*-guaiacylglyceryl) ether 7"-*O*-glucopyranoside (peak 19) | 24 |
| **Figure 20.** | ESI-QTof-MS spectrum of tricin-4'-*O*-(*threo*/*erythro*-*β*-*p*-hydroxyphenylglyceryl) ether 9"-*O*-*β*-D-glucopyranoside (peak 20) | 25 |
| **Figure 21.** | ESI-QTof-MS spectrum of tricin-4'-*O*-(*β*-guaiacylglyceryl) ether 9"-*O*-*β*-D-glucopyranoside (peak 21) | 26 |
| **Figure 22.** | ESI-QTof-MS spectrum of natasin A-4'-*O*-*β*-guaiacyl-(7"-*O*-methyl)-glyceryl ether (peak 22) | 27 |
| **Figure 23.** | ESI-QTof-MS spectrum of natasin A (peak 23) | 28 |
| **Figure 24.** | ESI-QTof-MS spectrum of natasin A-4'-*O*-*β*-*p*-hydroxyphenyl-(7"-*O*-methyl)-glyceryl] ether (peak 24) | 29 |
| **Figure 25.** | ESI-QTof-MS spectrum of tricin-2',3-dihydropropanol-2'-(4-hydroxy-3-methoxyphenyl) 4'-*O*-[*β*-guaiacyl-(7"-*O*-methyl)-glyceryl] ether (peak 25) | 30 |
| **Figure 26.** | ESI-QTof-MS spectrum of 5,6-dihydro-3,8,10-trihydroxy-5-(4-hydroxy-3-methoxyphenyl)-6-hydroxymethyl-2,4-dimethoxy-7H-benzo[c]xanthen-7-one (peak 26) | 31 |
| **Figure 27.** | ESI-QTof-MS spectrum of tricin (peak 27) | 32 |
|  |  | Page |
| **Figure 28.** | ESI-QTof-MS spectrum of tricin-4'-*O*-(*threo*-*β*-*p*-hydroxyphenylglyceryl) ether (peak 28) | 33 |
| **Figure 29.** | ESI-QTof-MS spectrum of tricin-4'*O*-(*threo*-*β*-guaiacylglyceryl) ether (peak 29) | 34 |
| **Figure 30.** | ESI-QTof-MS spectrum of luteolin-3',4'-dimethylether-7-*O*-glucoside (peak 30) | 35 |
| **Figure 31.** | ESI-QTof-MS spectrum of tricin-4'-*O*-(*erythro*-*β*-*p*-hydroxyphenylglyceryl) ether (peak 31) | 36 |
| **Figure 32.** | ESI-QTof-MS spectrum of tricin-4'-*O*-(*erythro*-*β*-guaiacylglyceryl) ether (peak 32) | 37 |
| **Figure 33.** | ESI-QTof-MS spectrum of tricin-4'-*O*-(*C*-veratroylglycol) ether (peak 33) | 38 |
| **Figure 34.** | ESI-QTof-MS spectrum of pinellic acid (isomer 1) (peak 34) | 39 |
| **Figure 35.** | ESI-QTof-MS spectrum of pinellic acid (isomer 2) (peak 35) | 40 |
| **Figure 36.** | ESI-QTof-MS spectrum of pinellic acid (isomer3) (peak 36) | 41 |
| **Figure 37.** | ESI-QTof-MS spectrum of pinellic acid (isomer 4) (peak 37) | 42 |
| **Figure 38.** | ESI-QTof-MS spectrum of 5,8,12-trihydroxy-trans-9-octadecenoic acid (peak 38) | 43 |
| **Figure 39.** | ESI-QTof-MS spectrum of tricin-4'-*O*-[*threo*-*β*-*p*-hydroxyphenyl (7"-*O*-methyl)-glyceryl] ether (peak 39) | 44 |
| **Figure 40.** | ESI-QTof-MS spectrum of tricin-4'-*O*-[*erythro*-*β*-*p*-hydroxyphenyl-(7"-*O*-methyl)-glyceryl] ether (peak 40) | 45 |
| **Figure 41.** | ESI-QTof-MS spectrum of tricin-4'-*O*-[*threo*-*β*-guaiacyl-(7"-*O*-methyl)-glyceryl] ether (peak 41) | 46 |
| **Figure 42.** | ESI-QTof-MS spectrum of tricin-4'-*O*-[ery*thro-β*-guaiacyl-(7"-*O*-methyl)-glyceryl] ether (peak 42) | 47 |
| **Figure 43.** | ESI-QTof-MS spectrum of tricin-4'-*O*-[*erythro*-*β*-guaiacyl-(9"-*O*-acetyl)-glyceryl] ether (peak 43) | 48 |
| **Figure 44.** | ESI-QTof-MS spectrum of tricin-4'-*O*-[*threo*-*β*-guaiacyl-(9"-*O*-acetyl)-glyceryl] ether (peak 44) | 49 |
| **Figure 45.** | ESI-QTof-MS spectrum of trihydroxy octadecenoic acid (isomer 1) (peak 45) | 50 |
| **Figure 46.** | ESI-QTof-MS spectrum of trihydroxy octadecenoic acid (isomer 2) (peak 46) | 51 |
| **Figure 47.** | ESI-QTof-MS spectrum of trihydroxy octadecenoic acid (isomer 3) (peak 47) | 52 |
| **Figure 48.** | ESI-QTof-MS spectrum of trihydroxy octadecenoic acid (isomer 4) (peak 48) | 53 |
| **Figure 49.** | ESI-QTof-MS spectrum of tricin-4'-*O*-[*threo*-*β*-guaiacyl-(7"-*O*-methyl-9"-*O*-acetyl)-glyceryl] ether (peak 49) | 54 |
| **Figure 50.** | ESI-QTof-MS spectrum of tricin-4'-*O*-[*erythro*-*β*-guaiacyl-(7"-*O*-methyl-9"-*O*-acetyl)-glyceryl] ether (peak 50) | 55 |
| **Figure 51.** | ESI-QTof-MS spectrum of 12,13-dihydroxy-9*Z*-octadecenoic acid (peak 51) | 56 |
| **Figure 52.** | ESI-QTof-MS spectrum of 1-(9*Z*,12*Z*,15*Z*-octadecadienoyl)-*sn*-glycero-3-phosphocholine (peak 52) | 57 |
| **Figure 53.** | ESI-QTof-MS spectrum of 9*E*,11*E*-13-hydroxyoctadecadienoic acid (peak 53) | 58 |
| **Figure 54.** | ESI-QTof-MS spectrum of 1-(9*Z*,12*Z*-octadecadienoyl)-*sn*-glycero-3-phosphocholine (peak 54) | 59 |
| **Figure 55.** | ESI-QTof-MS spectrum of 1-(9*Z*-octadecenoyl)-*sn*-glycero-3-phosphocholine (peak 55) | 60 |
| **Figure 56.** | The extracted ion chromatogram for *m/z* 509.145 and MS spectra of peaks 39 and 40. | 61 |
| **Figure 57.** | High definition MS^E^ spectrum and collision cross section value (CCS, Å^2^) of 4'-*O*-[*threo*-*β*-*p*-hydroxyphenyl-(7"-*O*-methyl)-glyceryl] ether (peak 39). | 62 |
|  |  |  |
|  |  | Page |
| **Figure 58.** | High definition MS^E^ spectrum and collision cross section value (CCS, Å^2^) of 4'-*O*-[*erythro*-*β*-*p*-hydroxyphenyl-(7"-*O*-methyl)-glyceryl] ether (peak 40). | 63 |
| **Figure 59.** | The extracted ion chromatogram for *m/z* 539.155 and MS spectra of peaks 41 and 42. | 64 |
| **Figure 60.** | High definition MS^E^ spectrum and collision cross section value (CCS, Å^2^) of tricin 4'-*O*-[*threo*-*β*-guaiacyl-(7"-*O*-methyl)-glyceryl] ether (peak 41). | 65 |
| **Figure 61.** | High definition MS^E^ spectrum and collision cross section value (CCS, Å^2^) of tricin 4'-*O*-[*eythro*-*β*-guaiacyl-(7"-*O*-methyl)-glyceryl] ether (peak 42). | 66 |
| **Figure 62.** | The extracted ion chromatogram for *m/z* 567.150 and MS spectra of peaks 43 and 44. | 67 |
| **Figure 63.** | High definition MS^E^ spectrum and collision cross section value (CCS, Å^2^) of tricin 4'-*O*-[*threo*-*β*-guaiacyl-(9"-*O*-acetyl)-glyceryl] ether (peak 44). | 68 |
| **Figure 64.** | The extracted ion chromatogram for *m/z* 581.166 and MS spectra of peaks 49 and 50. | 69 |
| **Figure 65.** | High definition MS^E^ spectrum and collision cross section value (CCS, Å^2^) of tricin 4'-*O*-[*threo*-*β*-guaiacyl-(7"-*O*-methyl-9"-*O*-acetyl)-glyceryl] ether (peak 49). | 70 |
| **Figure 66.** | High definition MS^E^ spectrum and collision cross section value (CCS, Å^2^) of tricin 4'-*O*-[*erythro*-*β*-guaiacyl-(7"-*O*-methyl-9"-*O*-acetyl)-glyceryl] ether (peak 50). | 71 |
| **Figure 67.** | The extracted ion chromatogram for *m/z* 495.129 and MS spectra of peaks 28 and 31. | 72 |
| **Figure 68.** | The extracted ion chromatogram for *m/z* 525.140 and MS spectra of peaks 29 and 32. | 73 |
| **Figure 69.** | The extracted ion chromatogram for *m/z* 687.193 and MS spectra of peaks 10, 13, 15, 17, 19, 21, 22, and 25. | 74 |
| **Figure 70.** | The extracted ion chromatogram for *m/z* 657.182 and MS spectra of peaks 12, 14, 16, 18, 20, and 24. | 76 |
| **Figure 71.** | The extracted ion chromatogram for *m/z* 657.182 and MS spectra of peaks 34‒38 and 45‒48. | 78 |
| **Figure 72.** | Principal component analysis (PCA) loading plot of metabolome analysis of the 983 wheat hull samples. | 80 |
| **Figure 73.** | ESI-QTof-MS spectrum of heptaethylene glycol (marker 1) | 81 |
| **Figure 74.** | Molecular formula for HRMS ion value of marker 1, which is analyzed in MassLynx (Waters Corporation, Milford, MA, USA). | 82 |
| **Figure 75.** | ESI-QTof-MS spectrum of heptaethylene glycol monomethyl ether (marker 2) | 83 |
| **Figure 76.** | Molecular formula for HRMS ion value of marker 2, which is analyzed in MassLynx (Waters Corporation, Milford, MA, USA). | 84 |
| **Figure 77.** | Compound list extracted from database of Global Natural Product Social Networking (GNPS). | 85 |
| **Figure 78.** | ESI-QTof-MS spectrum of 6-gingerol (marker 3) | 86 |
| **Figure 79.** | ESI-QTof-MS spectrum of 4-gingerol (marker 4) | 87 |
| **Figure 80.** | ESI-QTof-MS spectrum of phytuberin (marker 5) | 88 |
| **Figure 81.** | Molecular formula for HRMS ion value of marker 5, which is analyzed in MassLynx (Waters Corporation, Milford, MA, USA). | 89 |
|  |  | Page |
| **Figure 82.** | ESI-QTof-MS spectrum of unknown (marker 6) | 90 |
| **Figure 83.** | Molecular formula for HRMS ion value of marker 6, which is analyzed in MassLynx (Waters Corporation, Milford, MA, USA). | 91 |
| **Figure 84.** | Orthogonal partial least-squares discriminant analysis (OPLS-DA) S-plot between active and inactive groups. | 92 |
| **Figure 85.** | Orthogonal partial least-squares discriminant analysis (OPLS-DA) variable importance in projection (VIP) plot between active and inactive groups. | 93 |
| **Table 1.** | Nitric oxide (NO) production (% of cell viability) of the wheat hull of two original cultivars (WH01 and WH02) and its 983 mutant lines in lipopolysaccharide-stimulated RAW 264.7 macrophage cells. The values are expressed as the mean ± SD of three independent experiments. | 94 |

**
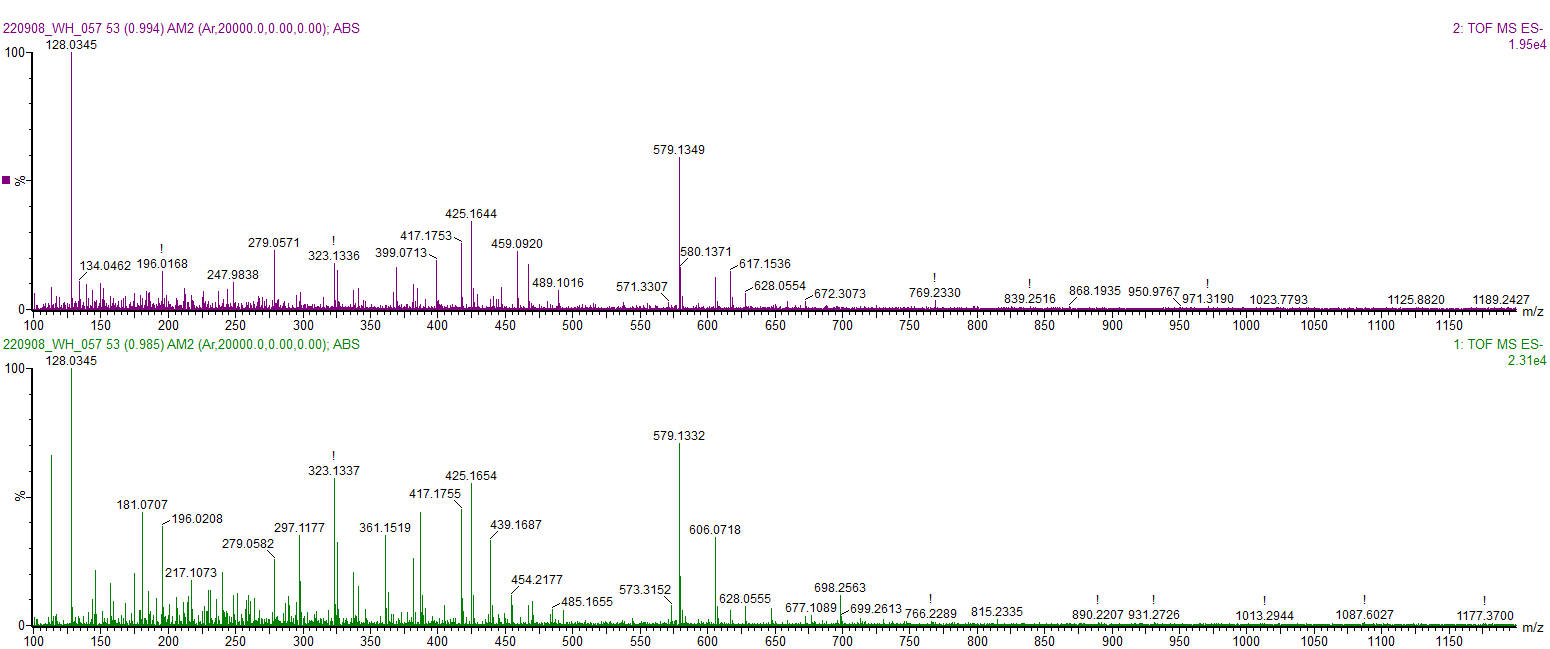
**

**Supplementary Figure 1.** ESI-QTof-MS spectrum of luteolin-6-*C*-arabinoside-8-*C*-glucoside (peak 1).

**
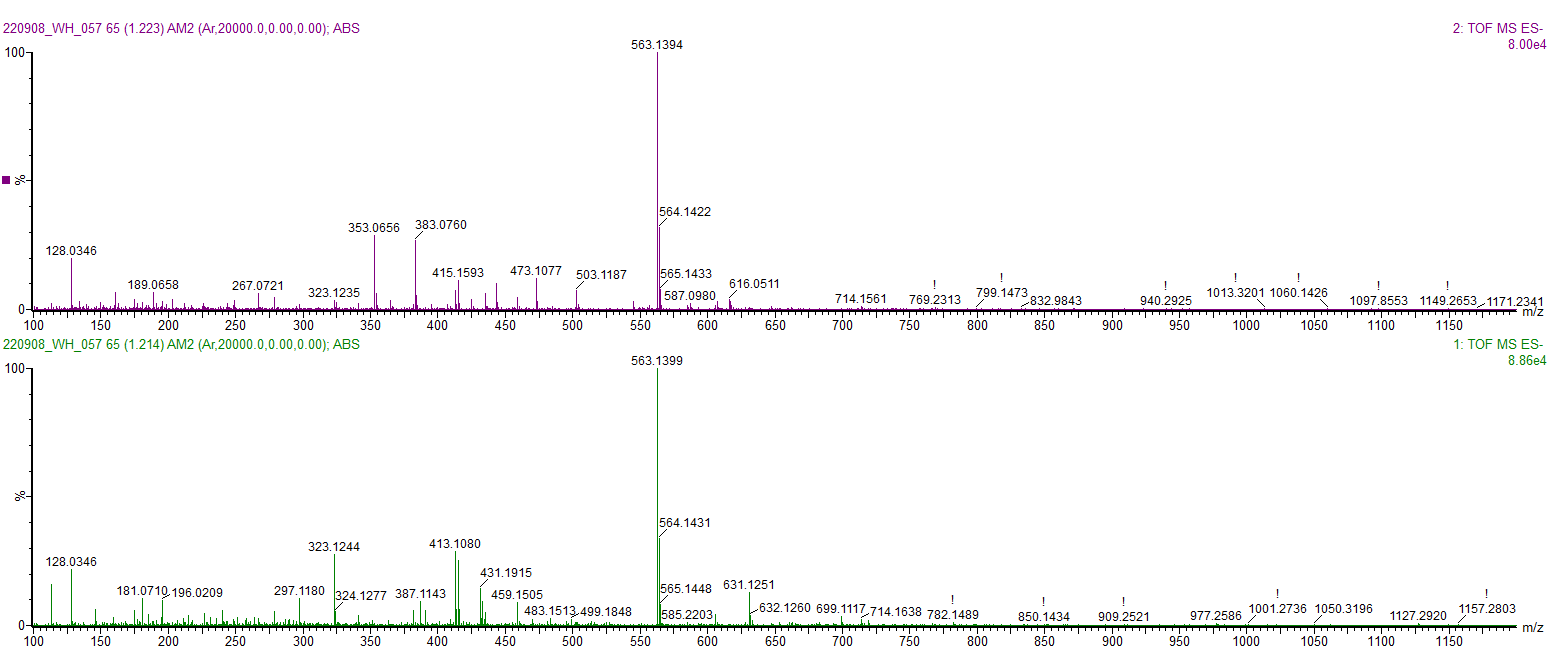
**

**Supplementary Figure 2.** ESI-QTof-MS spectrum of apigenin-6-*C*-arabinoside-8-*C-*glucoside (peak 2).


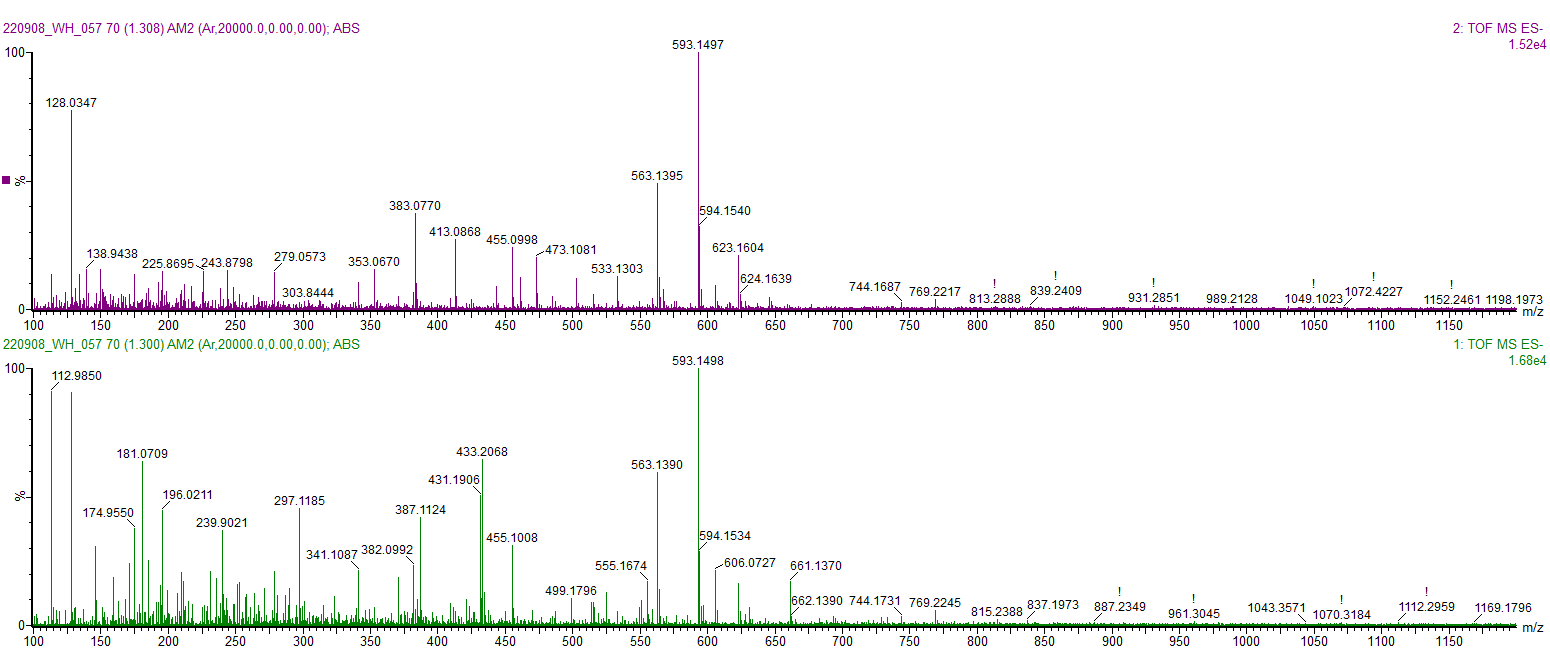


**Supplementary Figure 3.** ESI-QTof-MS spectrum of apigenin-8-*C*-glucoside-2"-*O*-glucoside (peak 3).

**
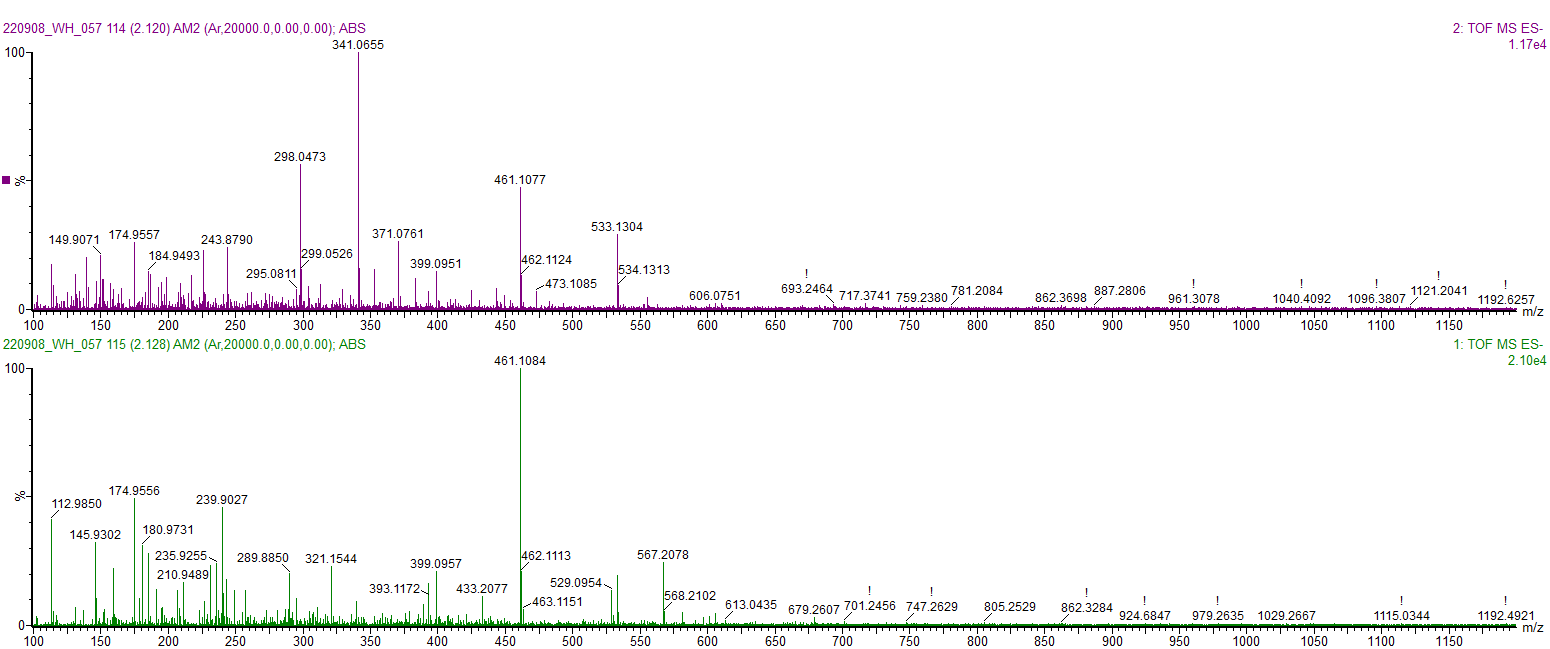
**

**Supplementary Figure 4.** ESI-QTof-MS spectrum of chrysoeriol-8-*C*-glucoside (peak 4).

**
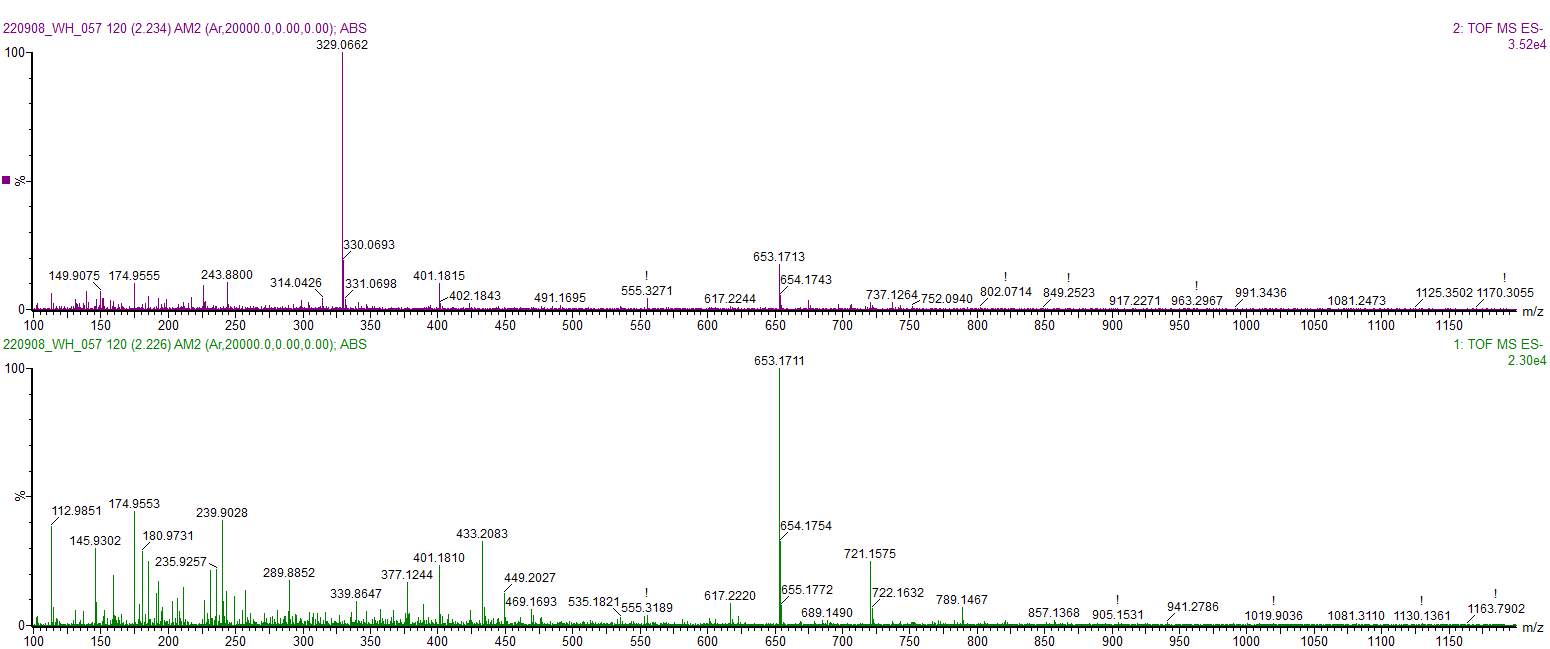
**

**Supplementary Figure 5.** ESI-QTof-MS spectrum of tricin-7-*O*-sophoroside (peak 5).

**
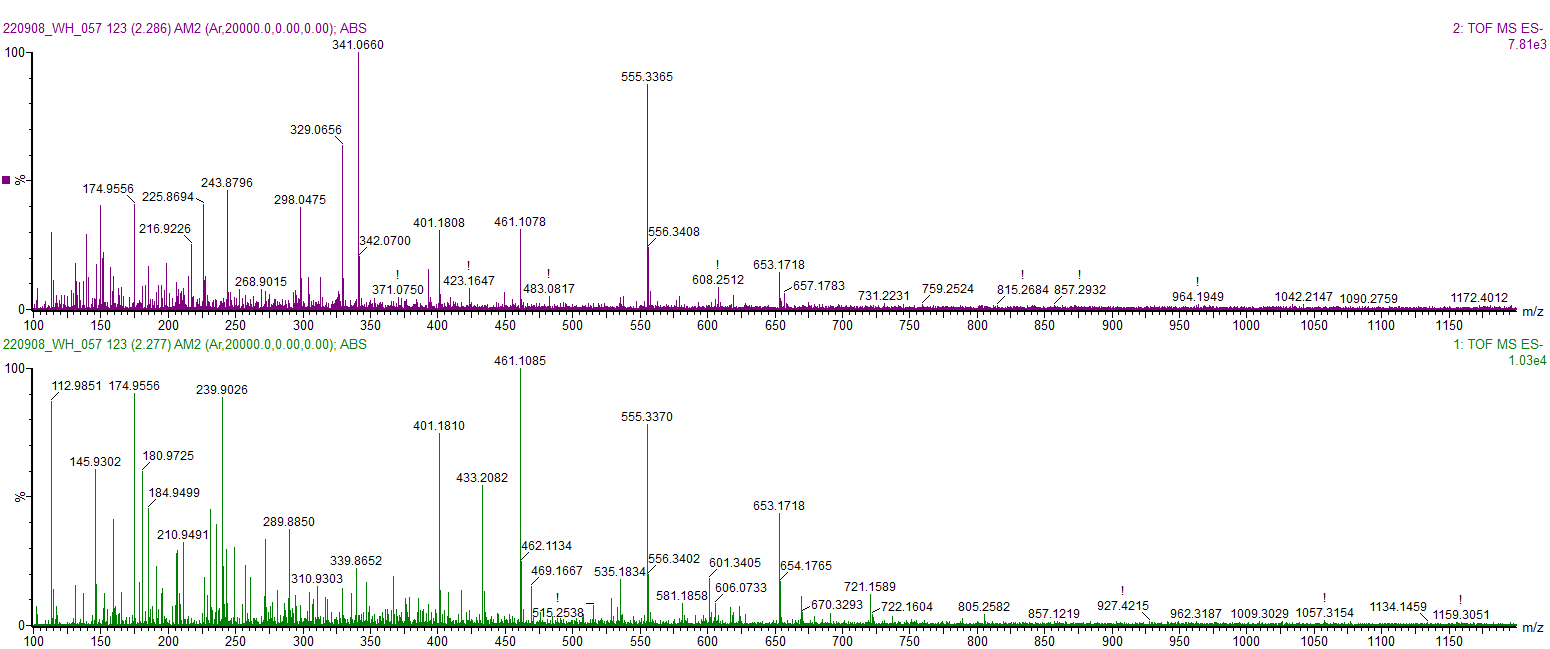
**

**Supplementary Figure 6.** ESI-QTof-MS spectrum of chrysoeriol-8-*C*-galactoside (peak 6).

**
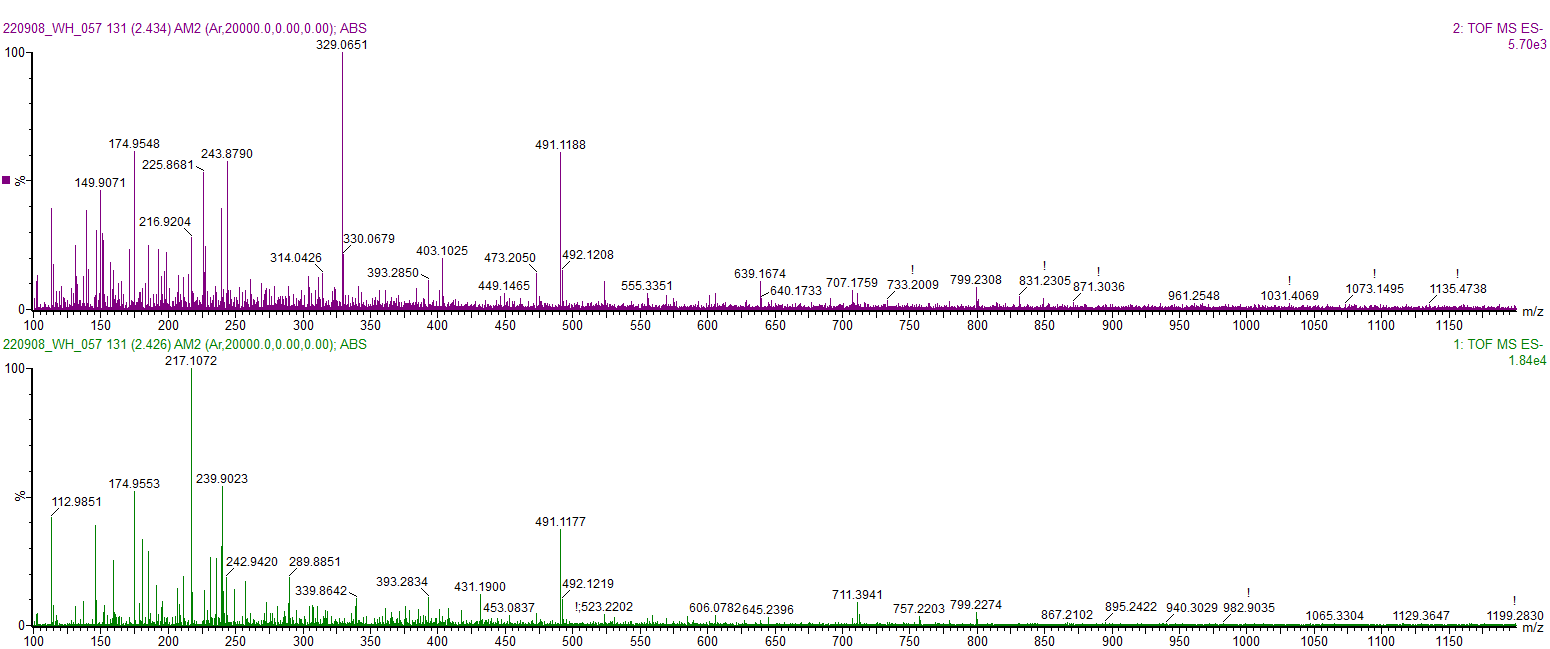
**

**Supplementary Figure 7.** ESI-QTof-MS spectrum of tricin-5-*O*-glucoside (peak 7).

**
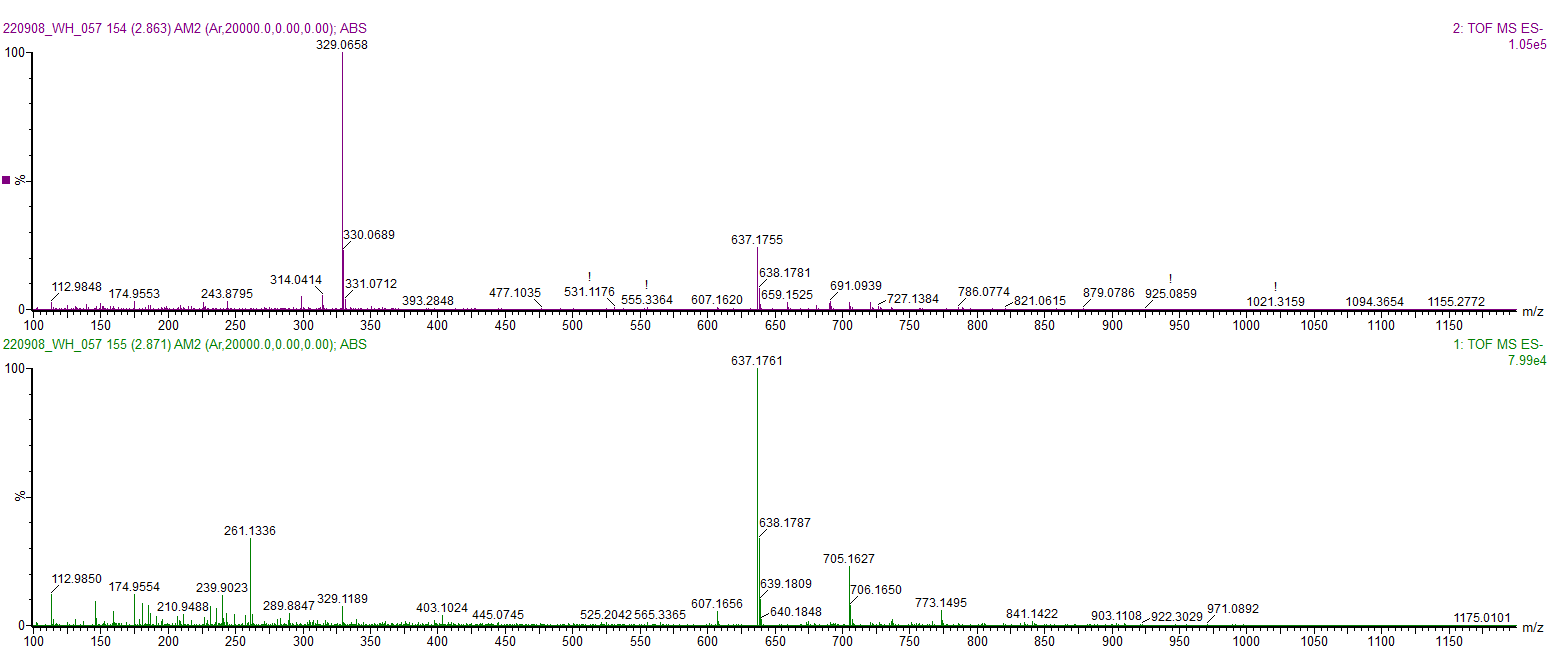
**

**Supplementary Figure 8.** ESI-QTof-MS spectrum of tricin-7-*O*-rutinoside (peak 8).

**
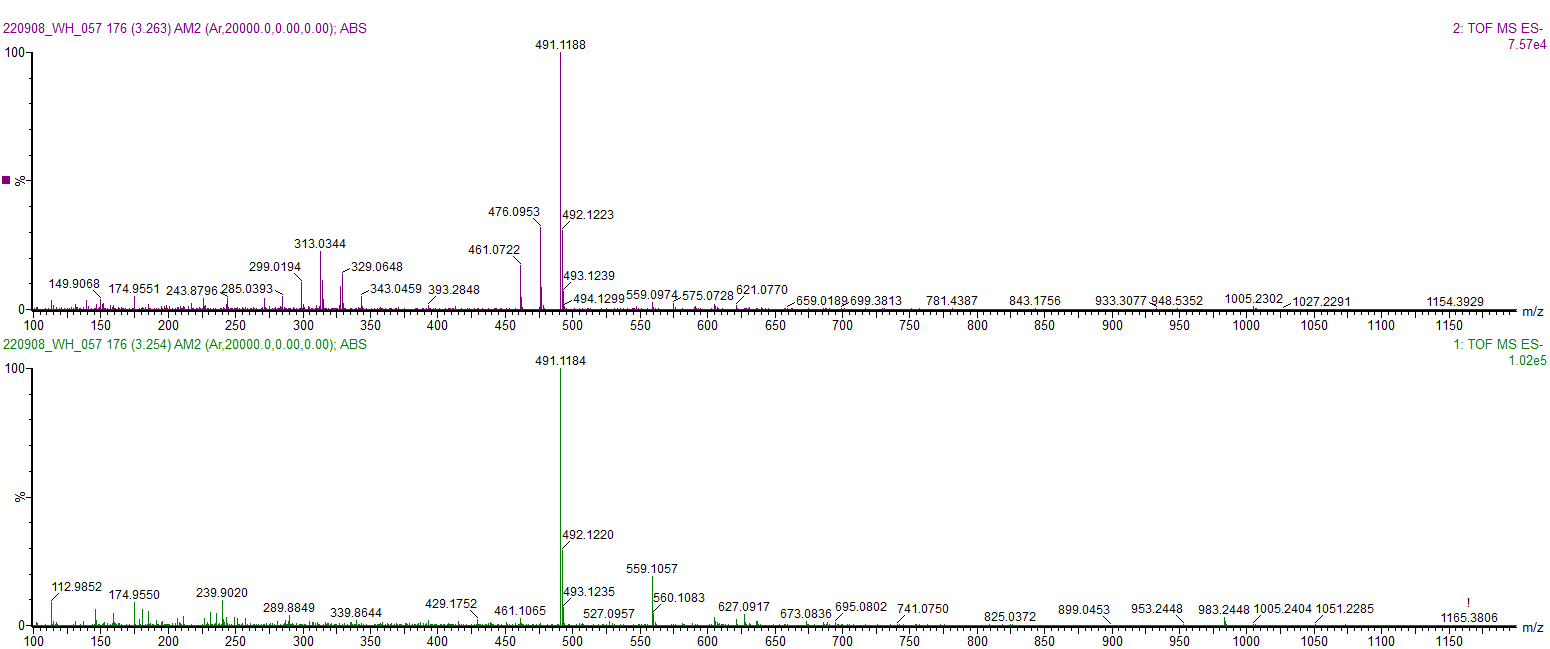
**

**Supplementary Figure 9.** ESI-QTof-MS spectrum of tricin-7-*O*-glucoside (peak 9).

**
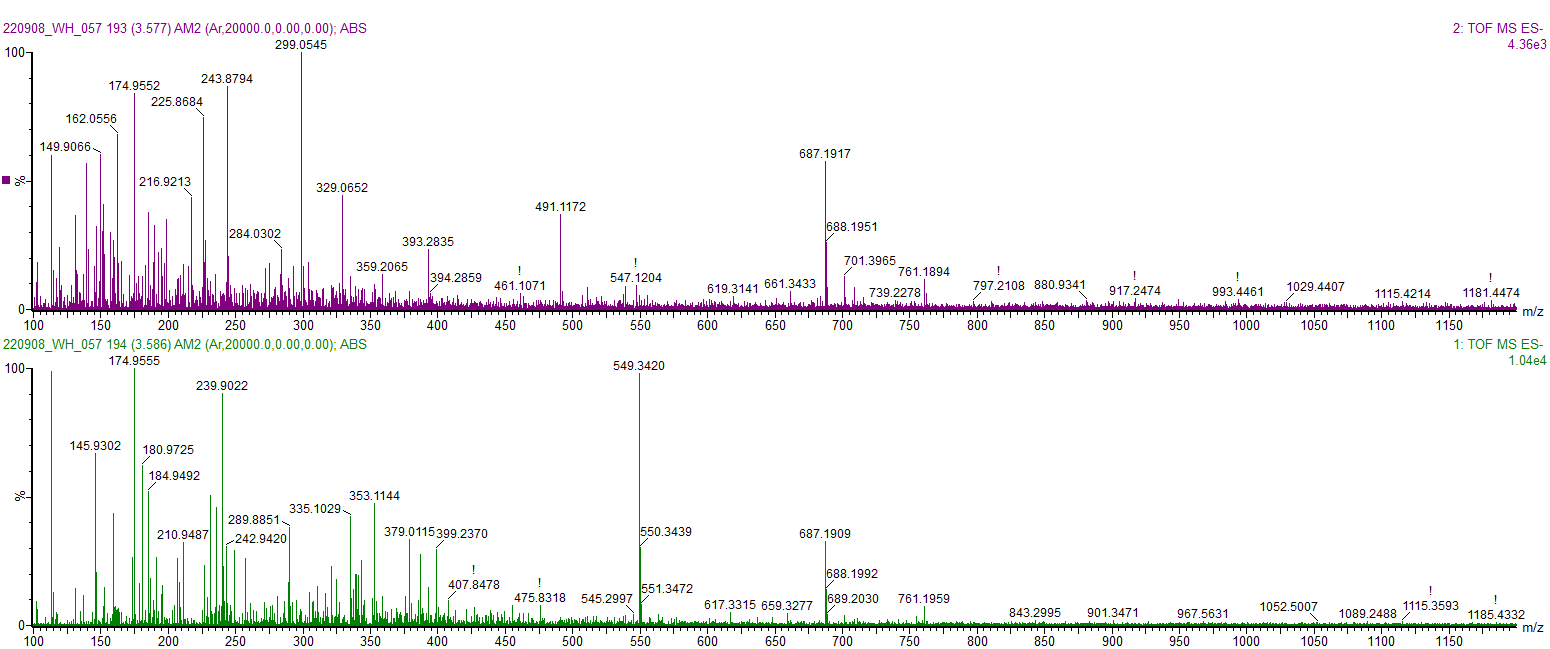
**

**Supplementary Figure 10.** ESI-QTof-MS spectrum of tricin-4'-*O*-(*β*-guaiacylglyceryl) ether 5-*O*-glucoside (peak 10).

**
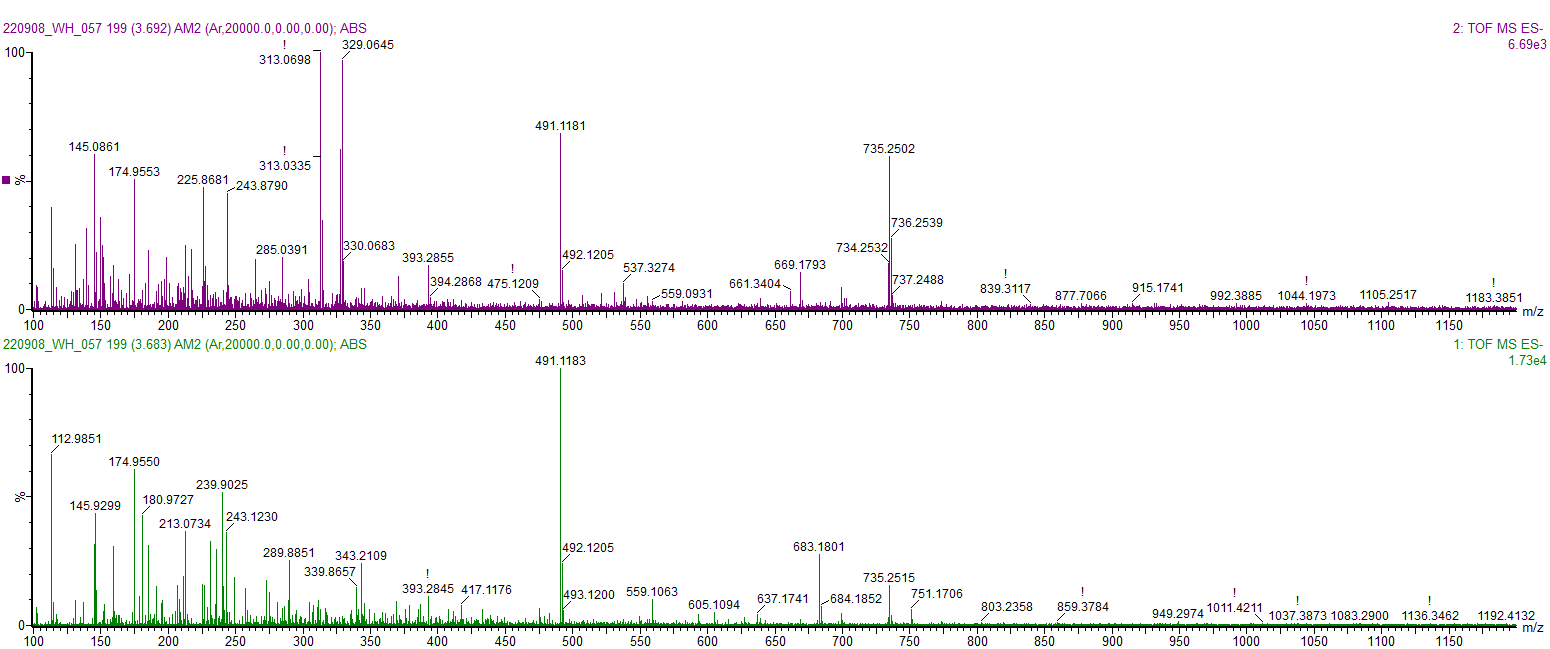
**

**Supplementary Figure 11.** ESI-QTof-MS spectrum of tricin-4'-*O*-glucoside (peak 11).

**
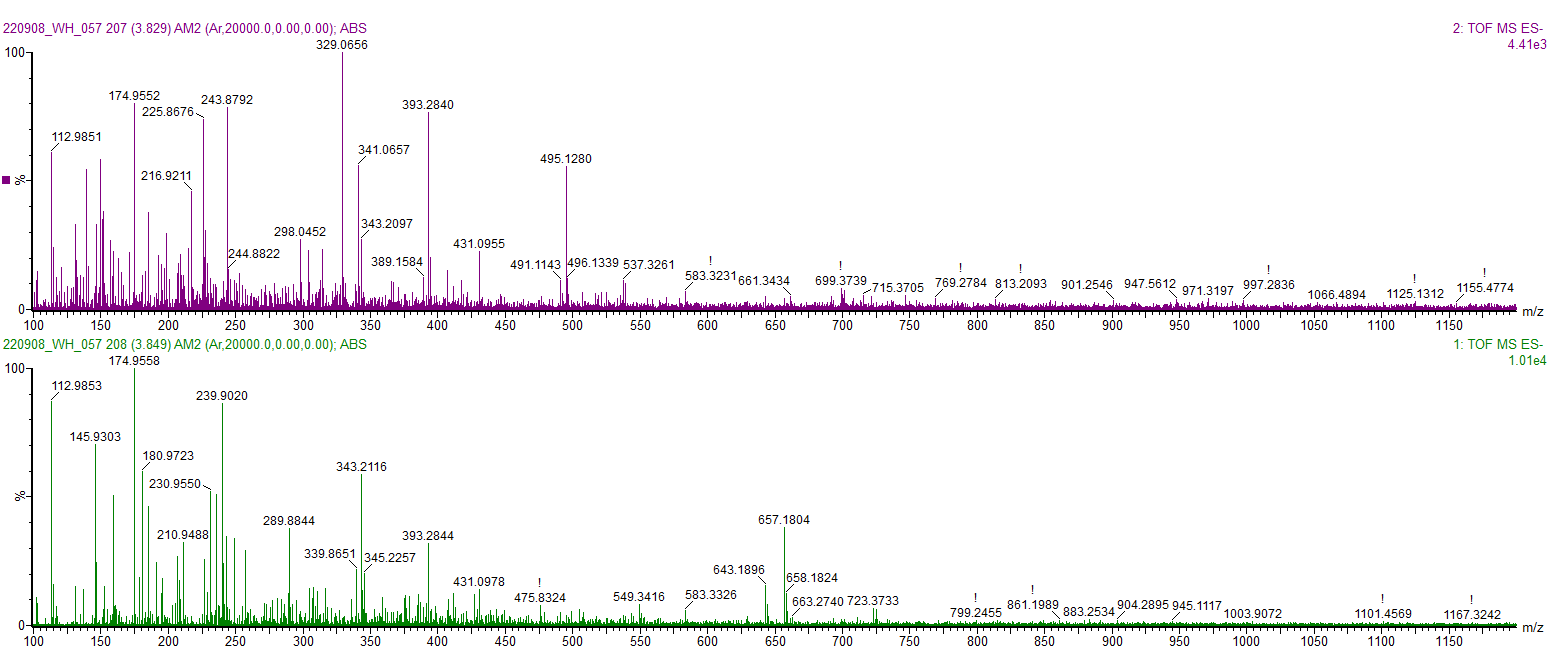
**

**Supplementary Figure 12.** ESI-QTof-MS spectrum of tricin-4'-*O*-(*threo*-*β*-*p*-hydroxyphenylglyceryl) ether 7-*O*-glucopyranoside (peak 12).

**
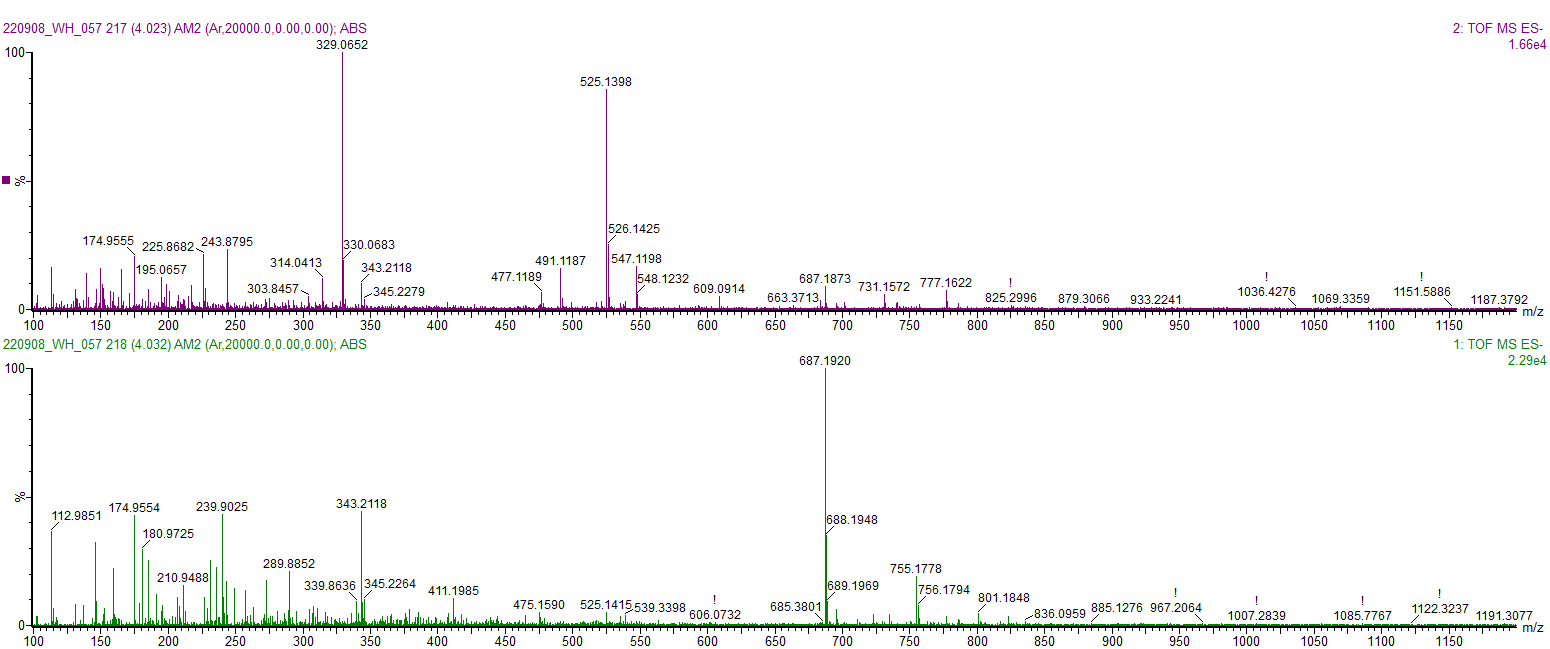
**

**Supplementary Figure 13.** ESI-QTof-MS spectrum of tricin-4'-*O*-(*threo*-*β*-guaiacylglyceryl) ether 7-*O*-glucoside (peak 13).

**
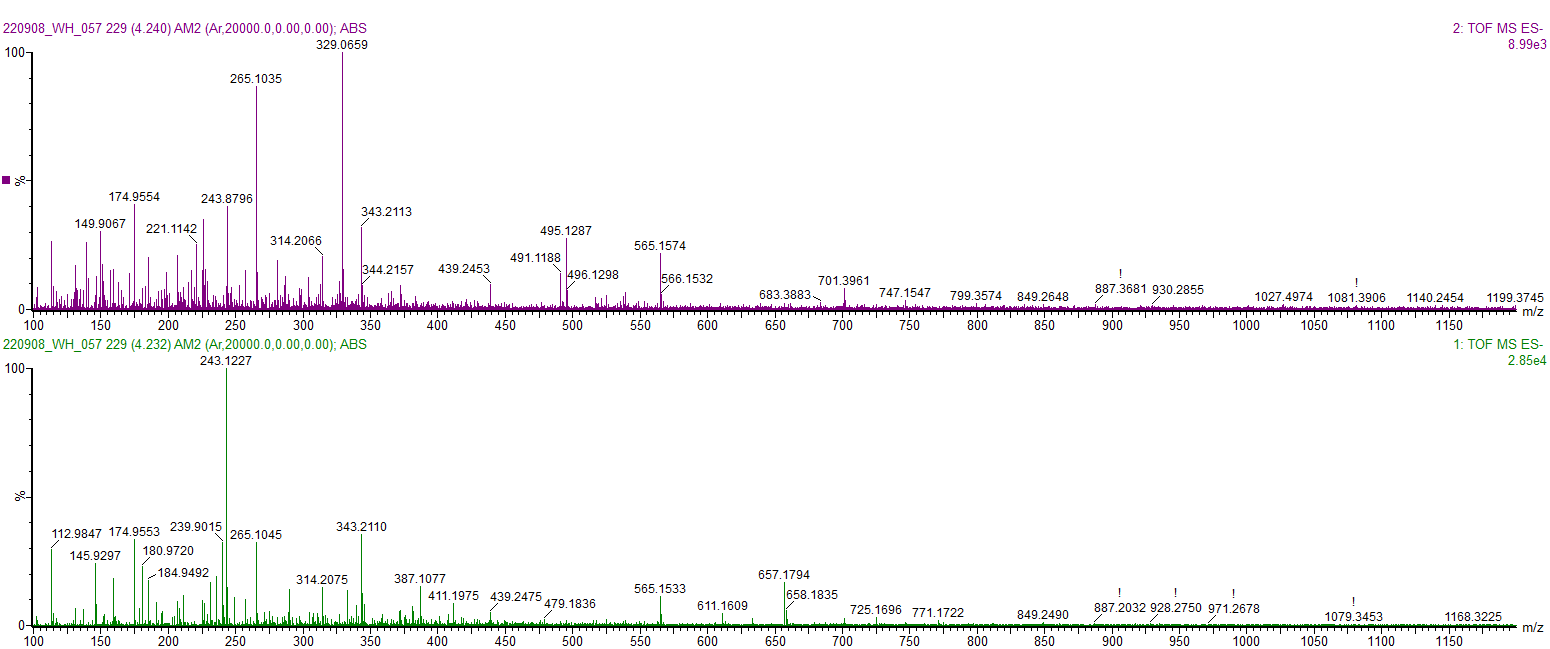
**

**Supplementary Figure 14.** ESI-QTof-MS spectrum of tricin-4'-*O*-(*erythro*-*β*-*p*-hydroxyphenylglyceryl) ether 7-*O*-glucopyranoside (peak 14).

**
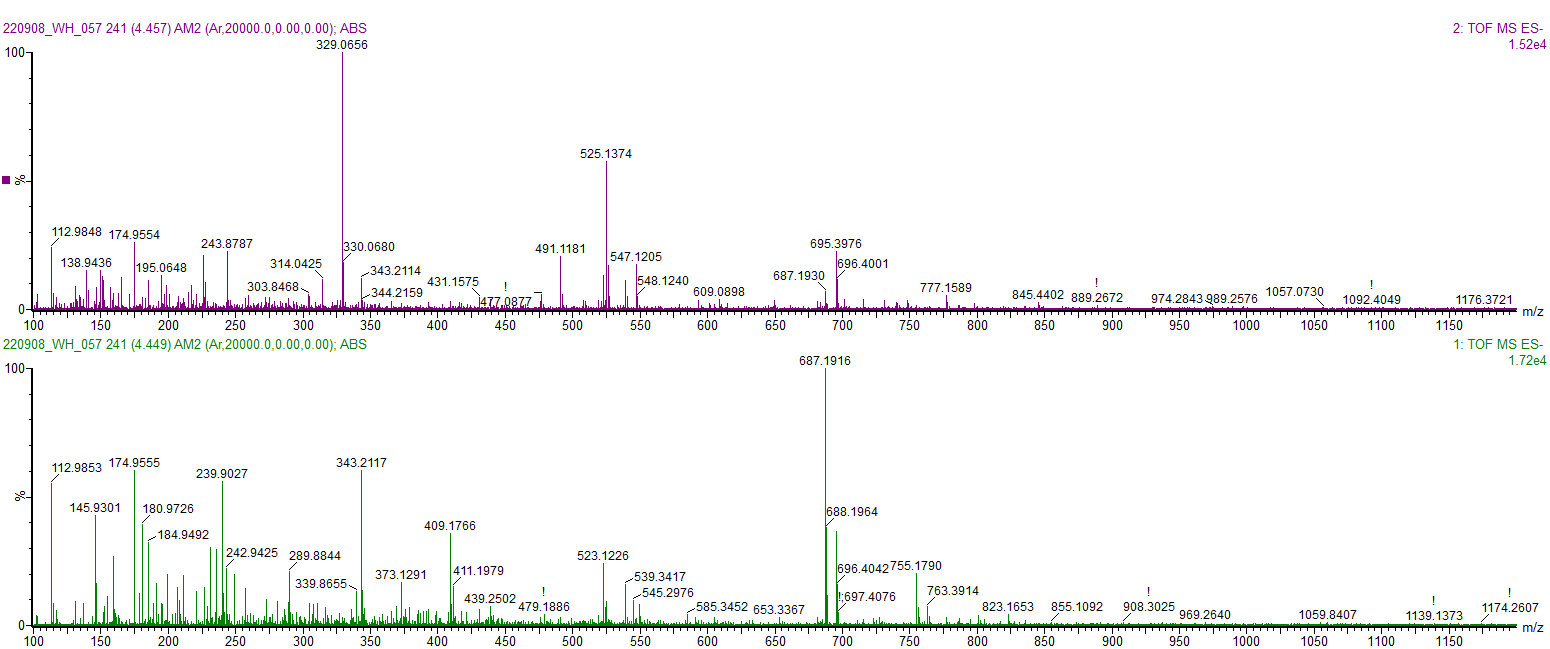
**

**Supplementary Figure 15.** ESI-QTof-MS spectrum of tricin-4'-*O*-(*erythro*-*β*-guaiacylglyceryl) ether 7-*O*-glucopyranoside (peak 15).

**
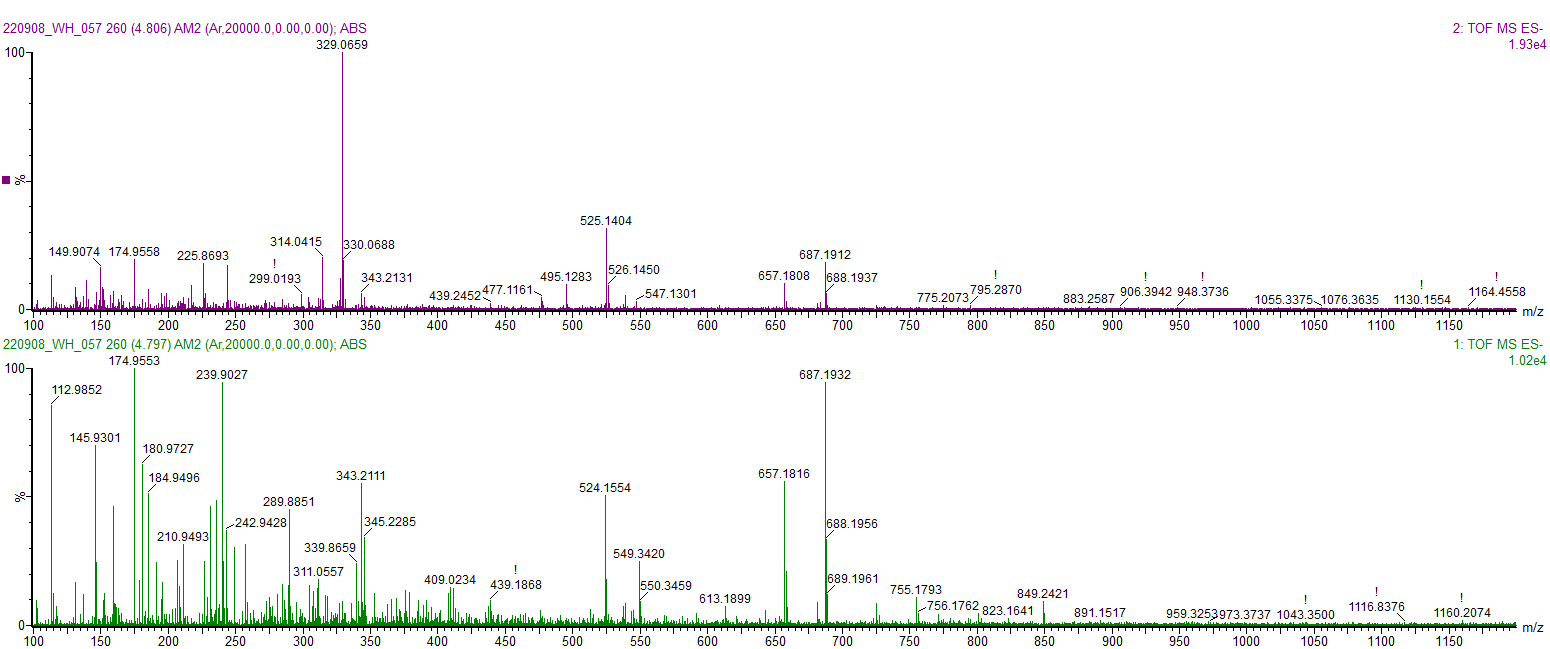
**

**Supplementary Figure 16.** ESI-QTof-MS spectrum of tricin-4'-*O*-(*threo*-*β*-*p*-hydroxyphenylglyceryl) ether 7"-*O*-glucopyranoside (peak 16).

**
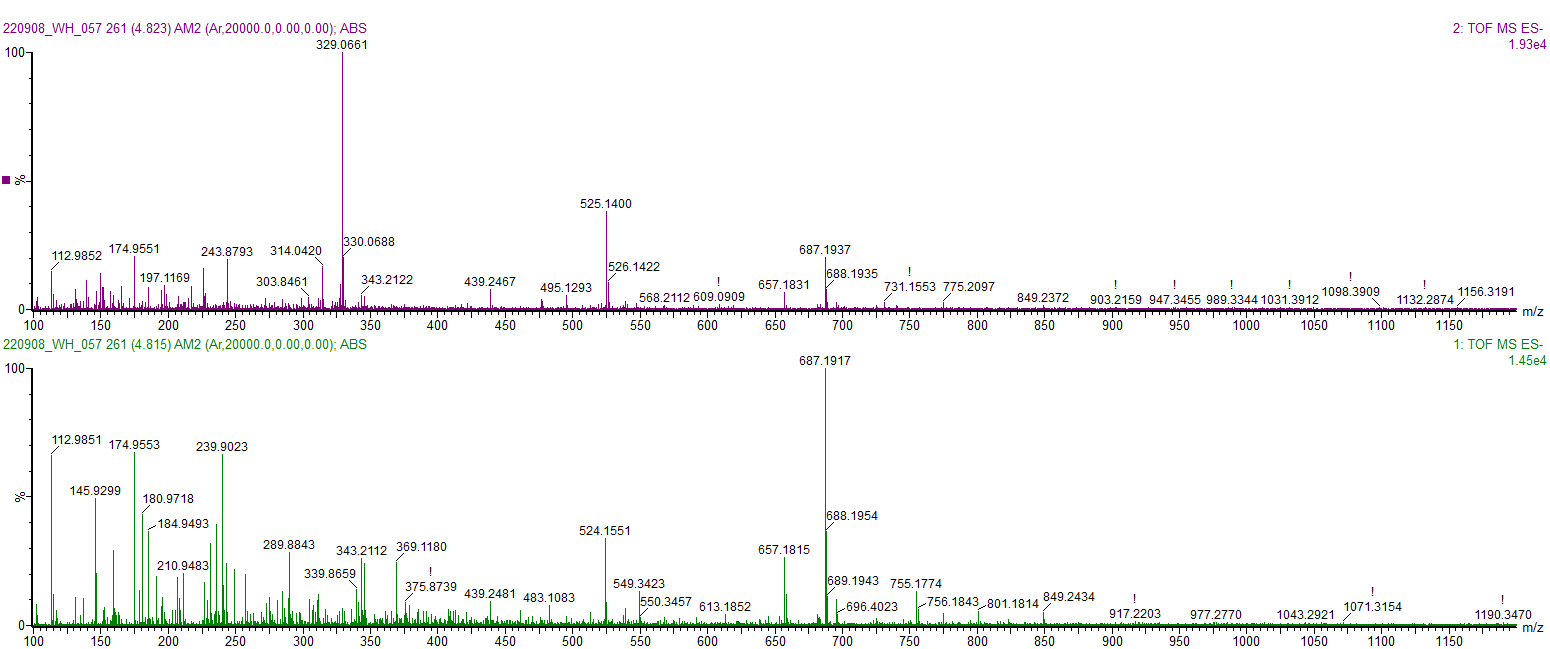
**

**Supplementary Figure 17.** ESI-QTof-MS spectrum of tricin-4'-*O*-(*threo*-*β*-guaiacylglyceryl) ether 7"-*O*-glucopyranoside (peak 17).

**
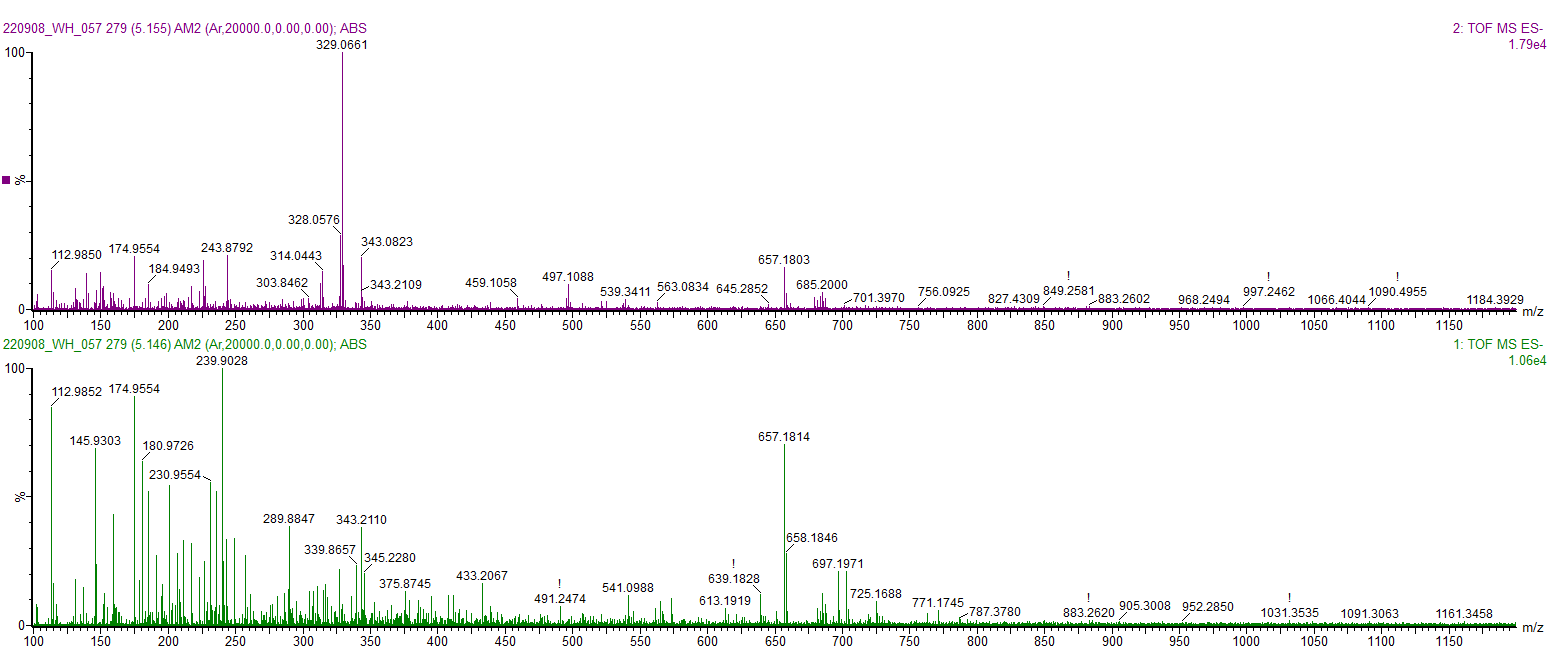
**

**Supplementary Figure 18.** ESI-QTof-MS spectrum of tricin-4'-*O*-(*erythro*-*β*-*p*-hydroxyphenylglyceryl) ether 7"-*O*-glucopyranoside (peak 18).

**
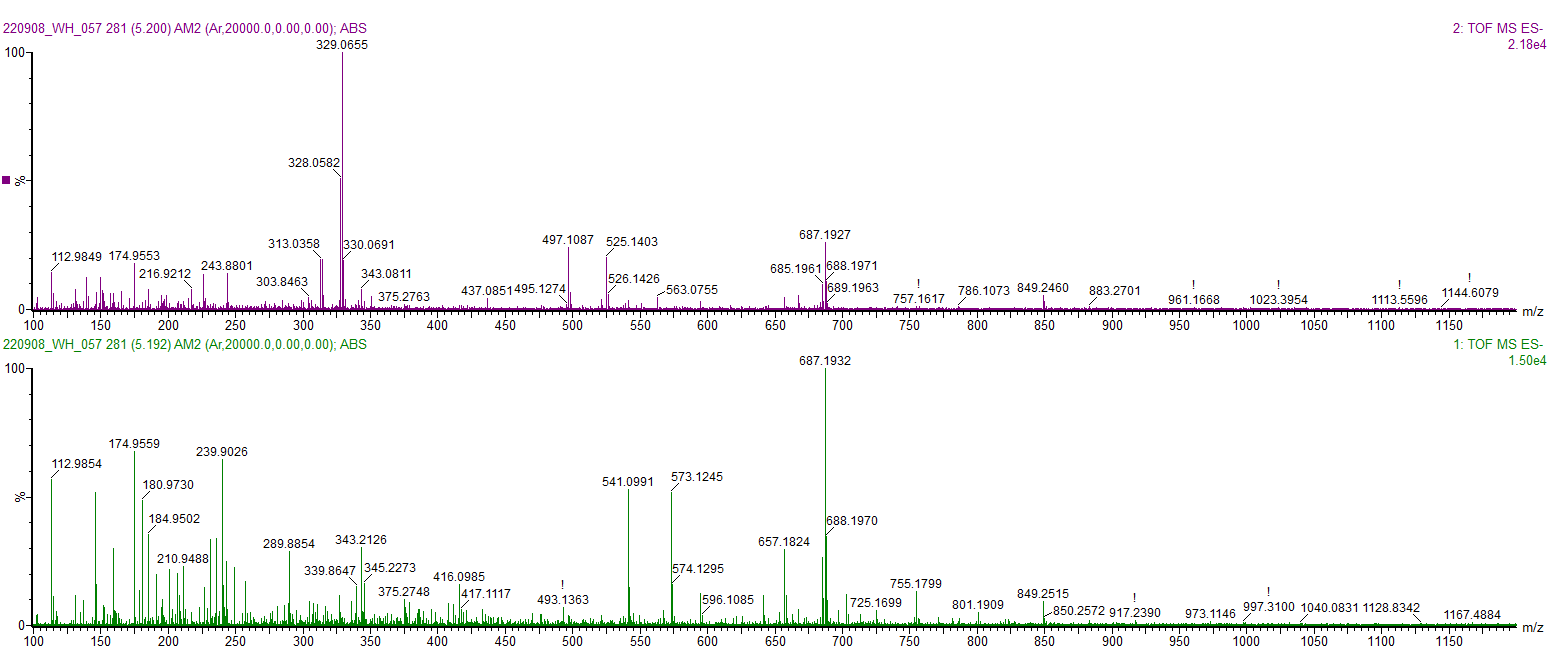
**

**Supplementary Figure 19.** ESI-QTof-MS spectrum of tricin-4'-*O*-(*erythro*-*β*-guaiacylglyceryl) ether 7"-*O*-glucopyranoside (peak 19).

**
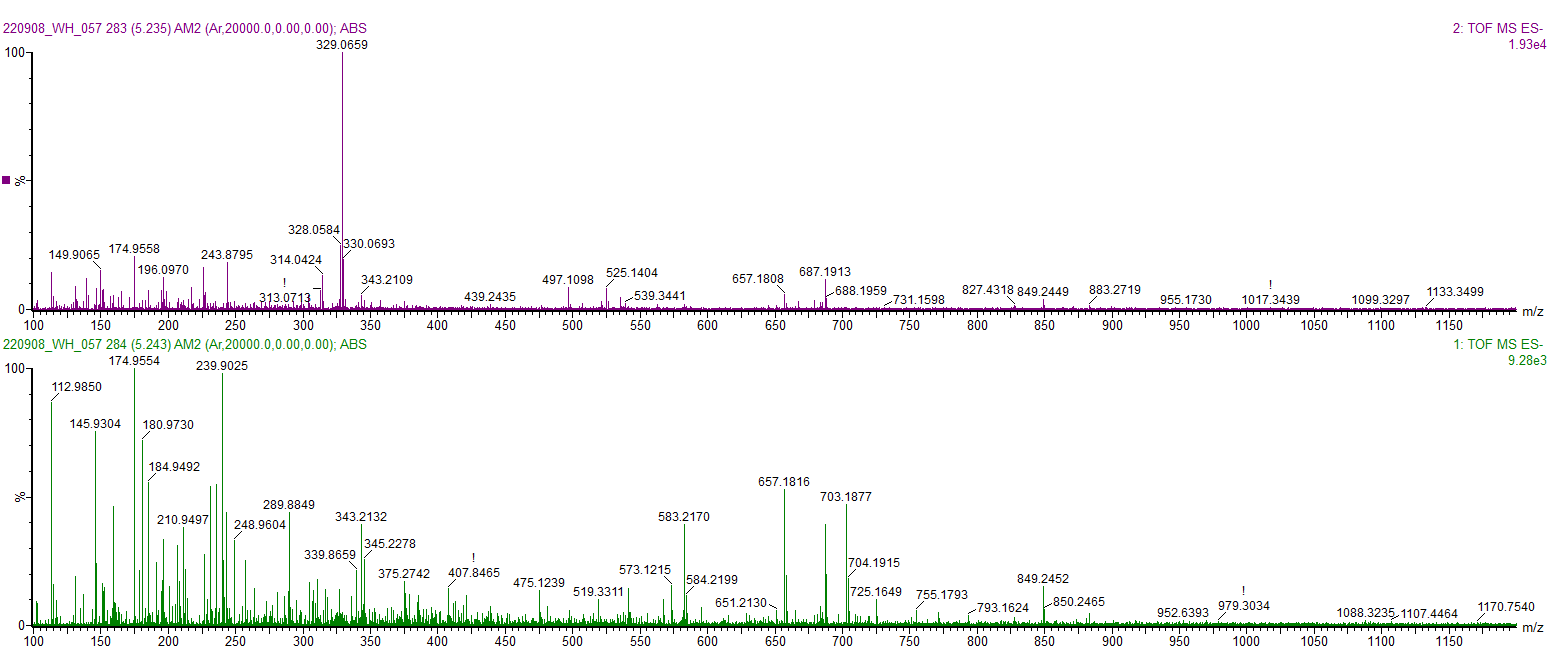
**

**Supplementary Figure 20.** ESI-QTof-MS spectrum of tricin-4'-*O*-(*threo*/*erythro*-*β*-*p*-hydroxyphenylglyceryl) ether 9"-*O*-*β*-D-glucopyranoside (peak 20).

**
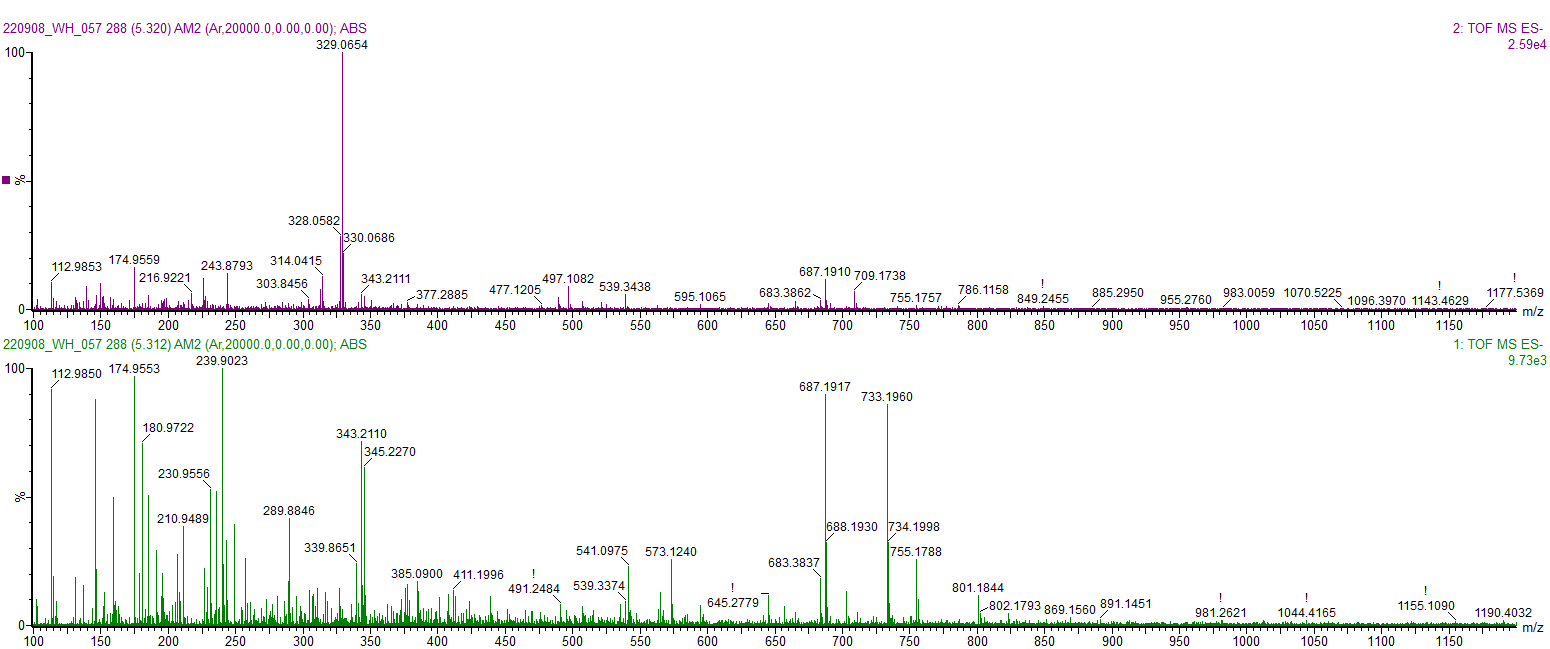
**

**Supplementary Figure 21.** ESI-QTof-MS spectrum of tricin-4'-*O*-(*β*-guaiacylglyceryl) ether 9"-*O*-*β*-D-glucopyranoside (peak 21).

**
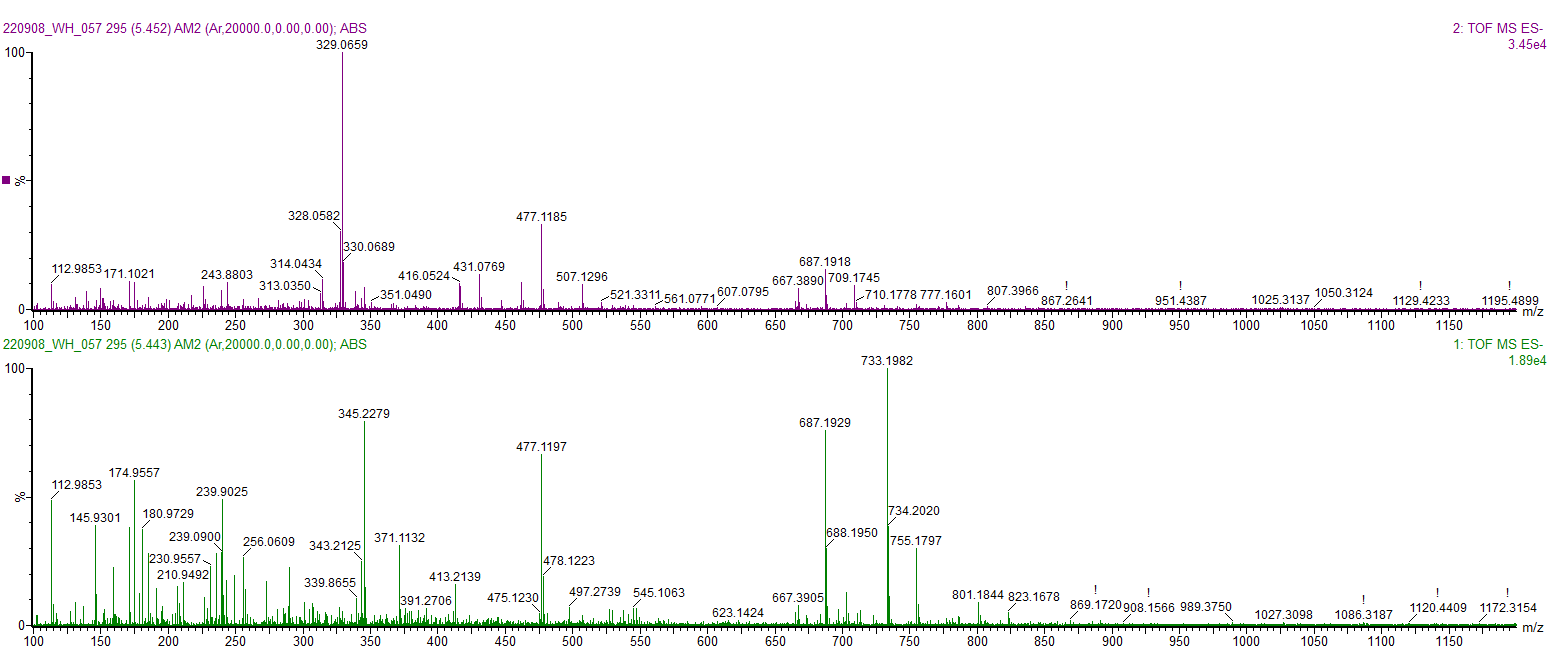
**

**Supplementary Figure 22.** ESI-QTof-MS spectrum of natasin A-4'-*O*-*β*-guaiacyl-(7"-*O*-methyl)-glyceryl ether (peak 22).

**
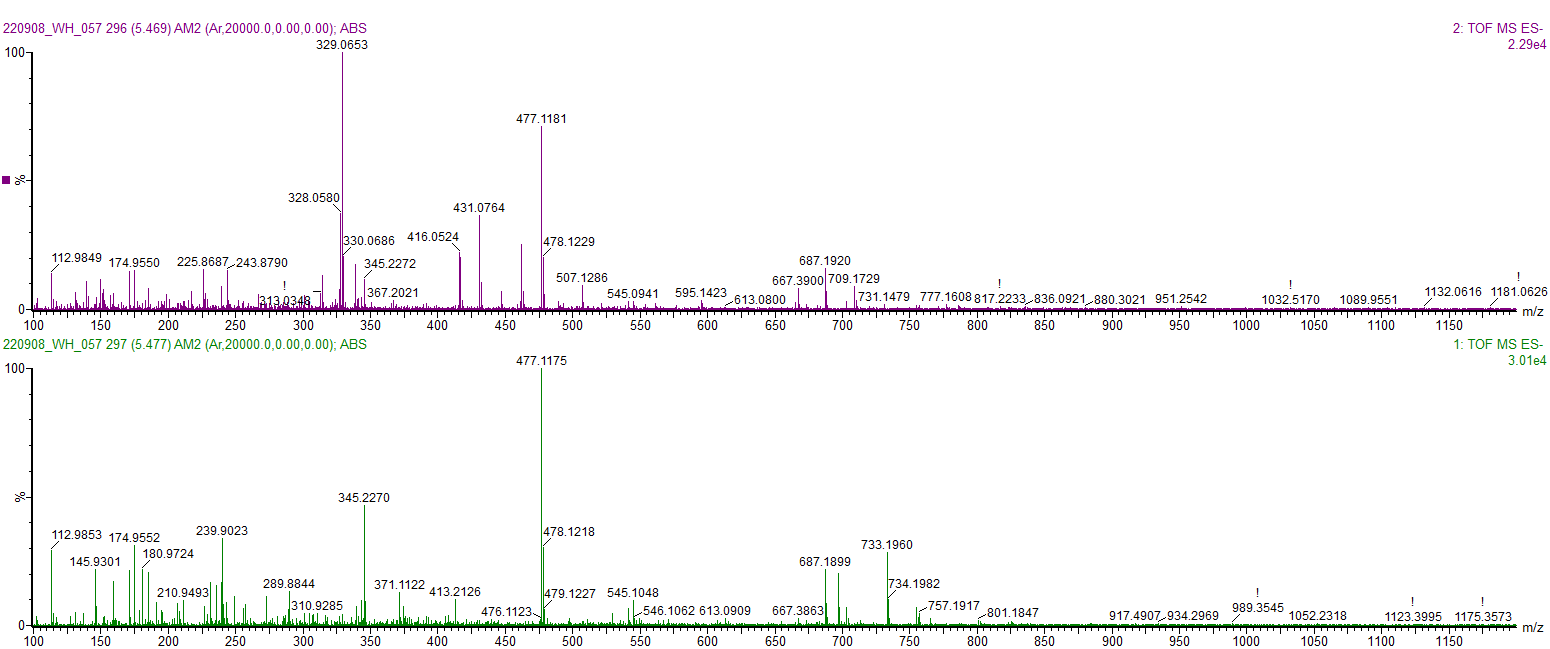
**

**Supplementary Figure 23.** ESI-QTof-MS spectrum of natasin A (peak 23).

**
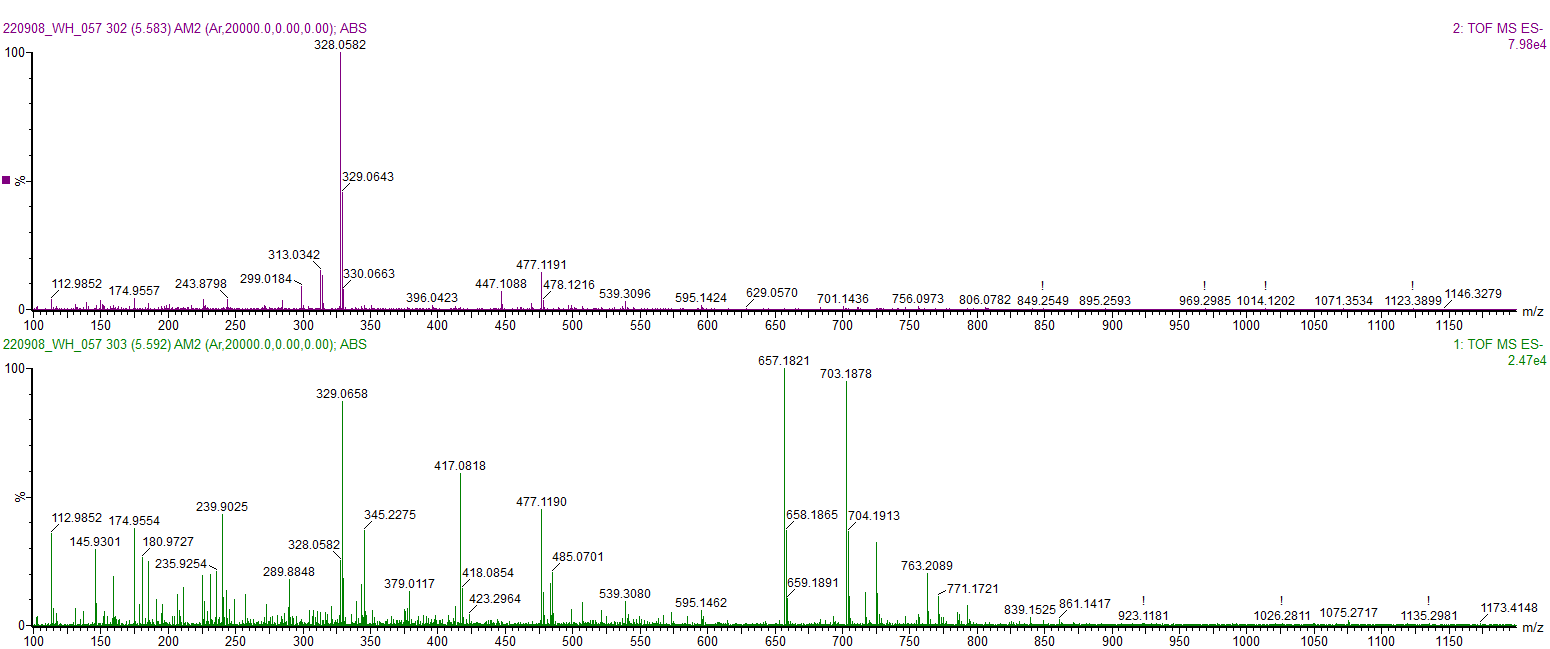
**

**Supplementary Figure 24.** ESI-QTof-MS spectrum of natasin A-4'-*O*-*β*-*p*-hydroxyphenyl-(7"-*O*-methyl)-glyceryl] ether (peak 24).

**
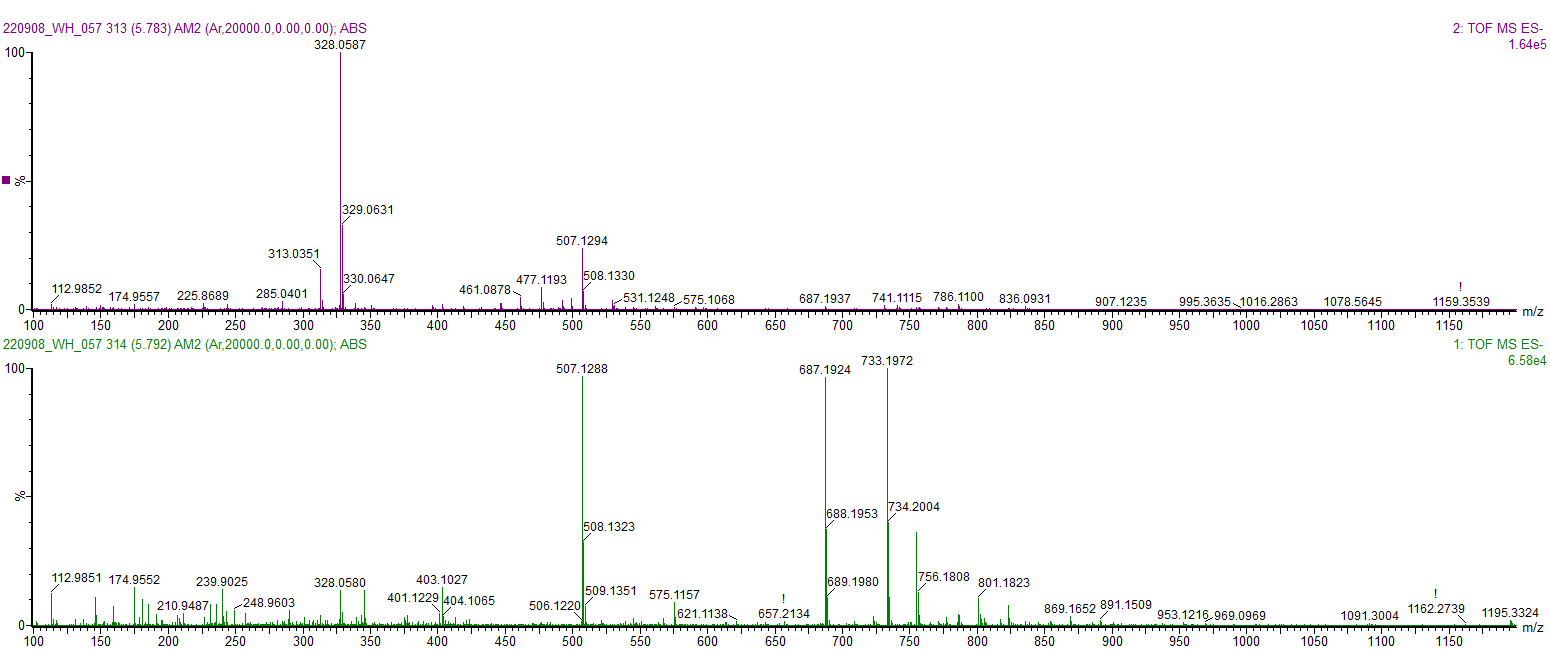
**

**Supplementary Figure 25.** ESI-QTof-MS spectrum of tricin-2',3-dihydropropanol-2'-(4-hydroxy-3-methoxyphenyl) 4'-*O*-[*β*-guaiacyl-(7"-*O*-methyl)-glyceryl] ether (peak 25).

**
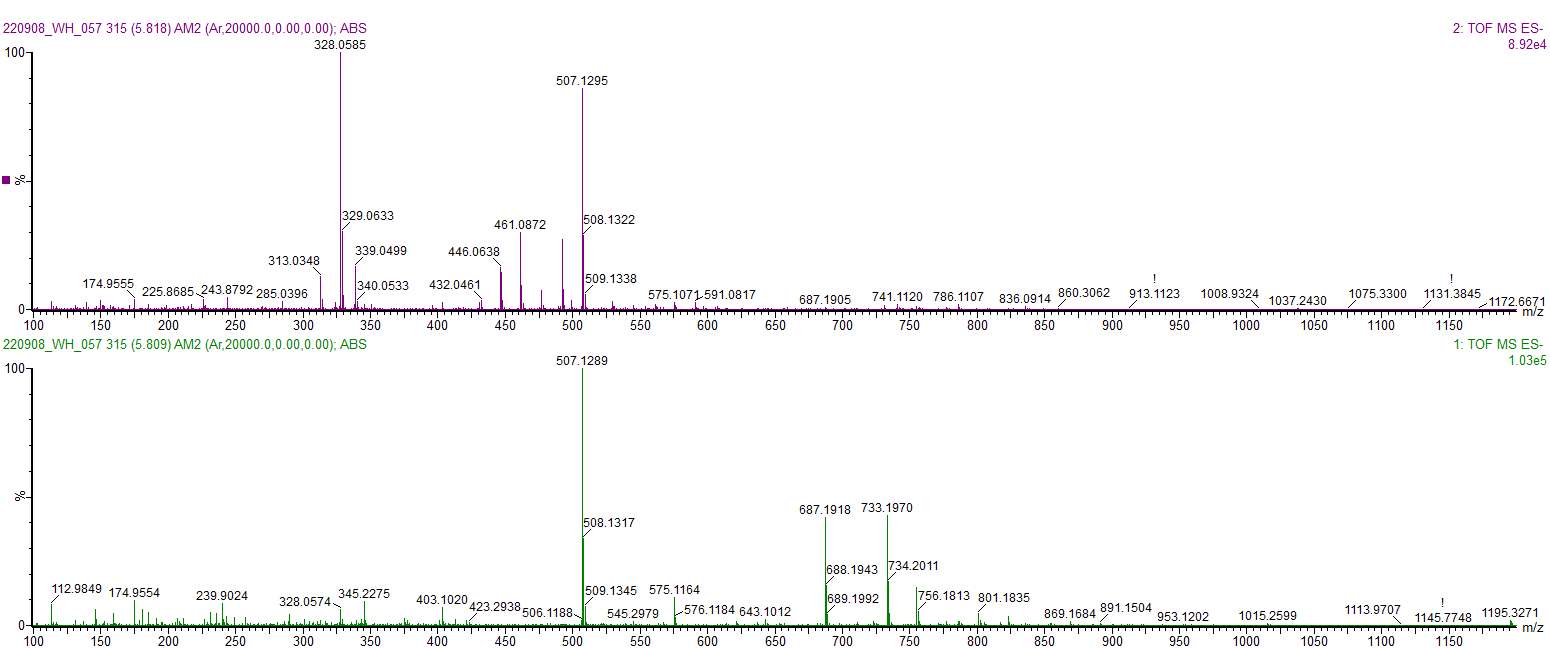
**

**Supplementary Figure 26.** ESI-QTof-MS spectrum of 5,6-dihydro-3,8,10-trihydroxy-5-(4-hydroxy-3-methoxyphenyl)-6-hydroxymethyl-2,4-dimethoxy-7H-benzo[c]xanthen-7-one (peak 26).

**
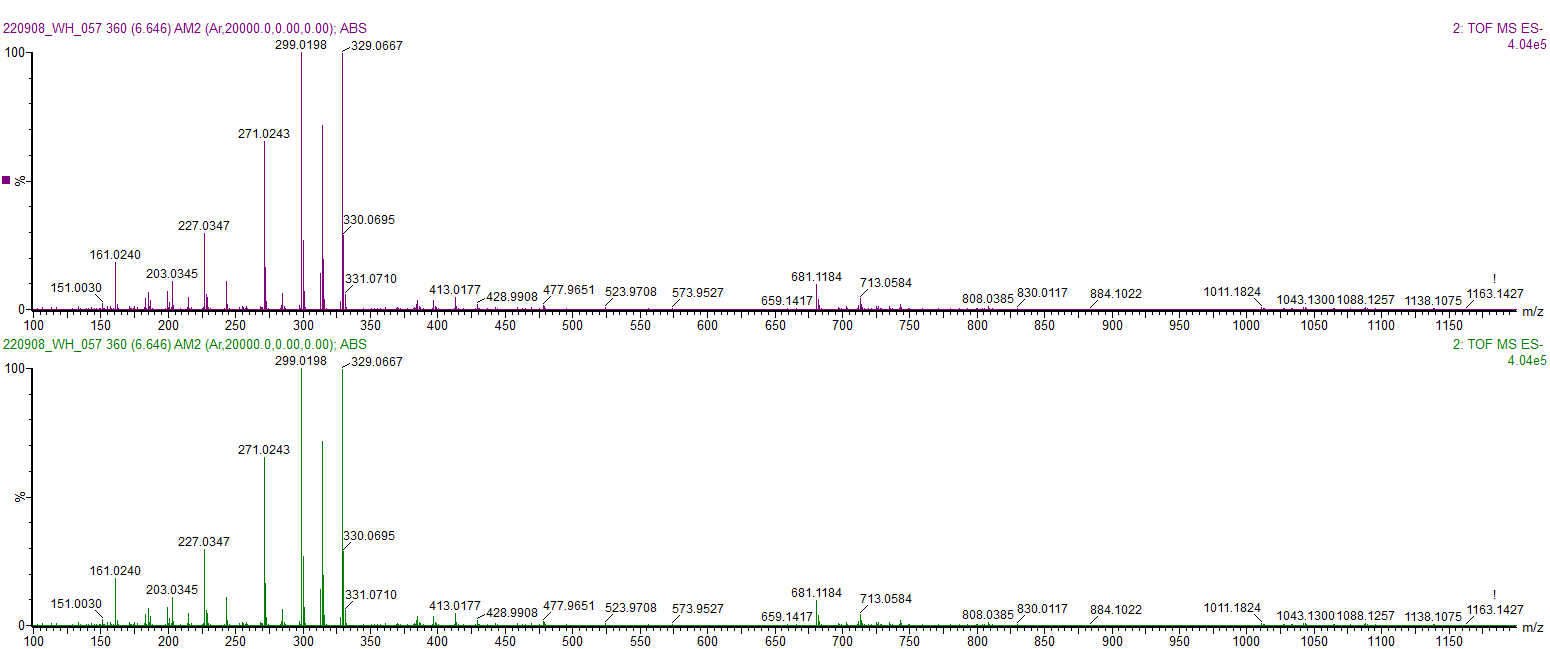
**

**Supplementary Figure 27.** ESI-QTof-MS spectrum of tricin (peak 27).

**
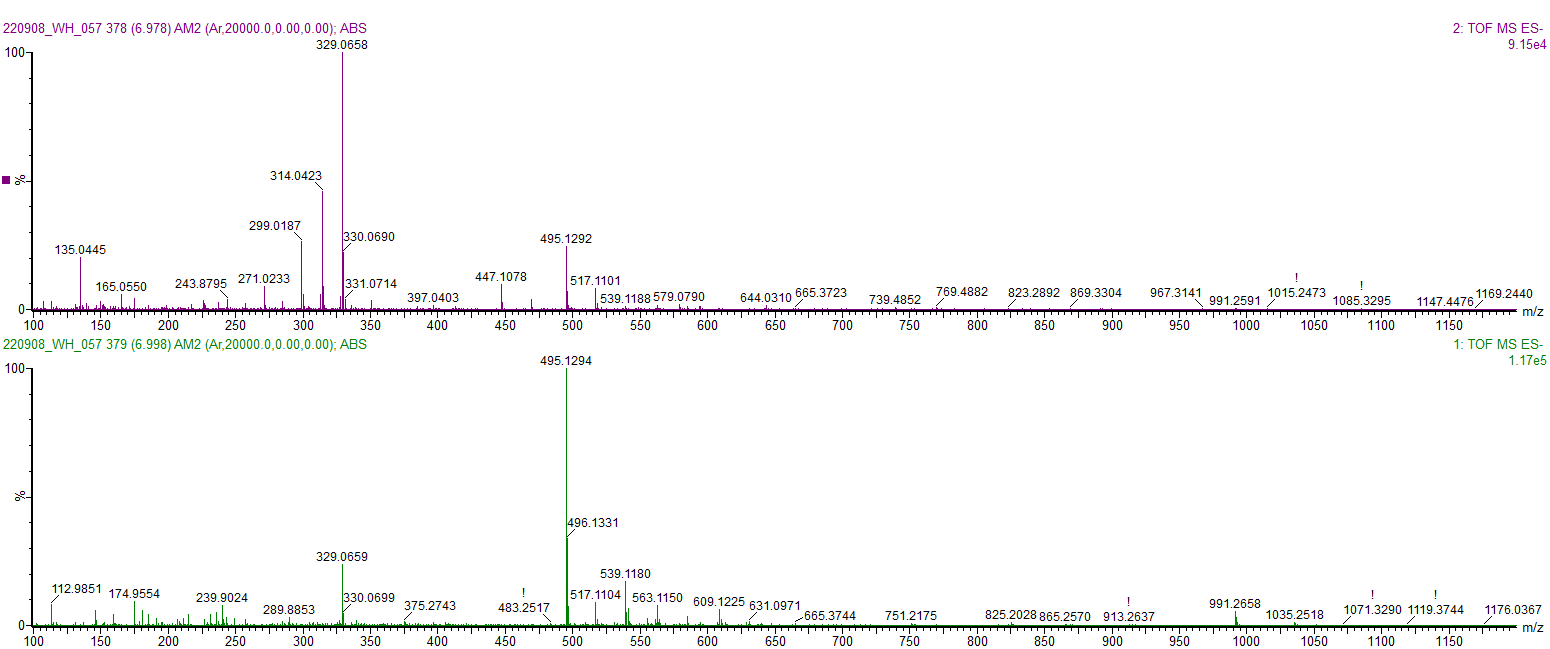
**

**Supplementary Figure 28.** ESI-QTof-MS spectrum of tricin-4'-*O*-(*threo*-*β*-*p*-hydroxyphenylglyceryl) ether (peak 28).

**
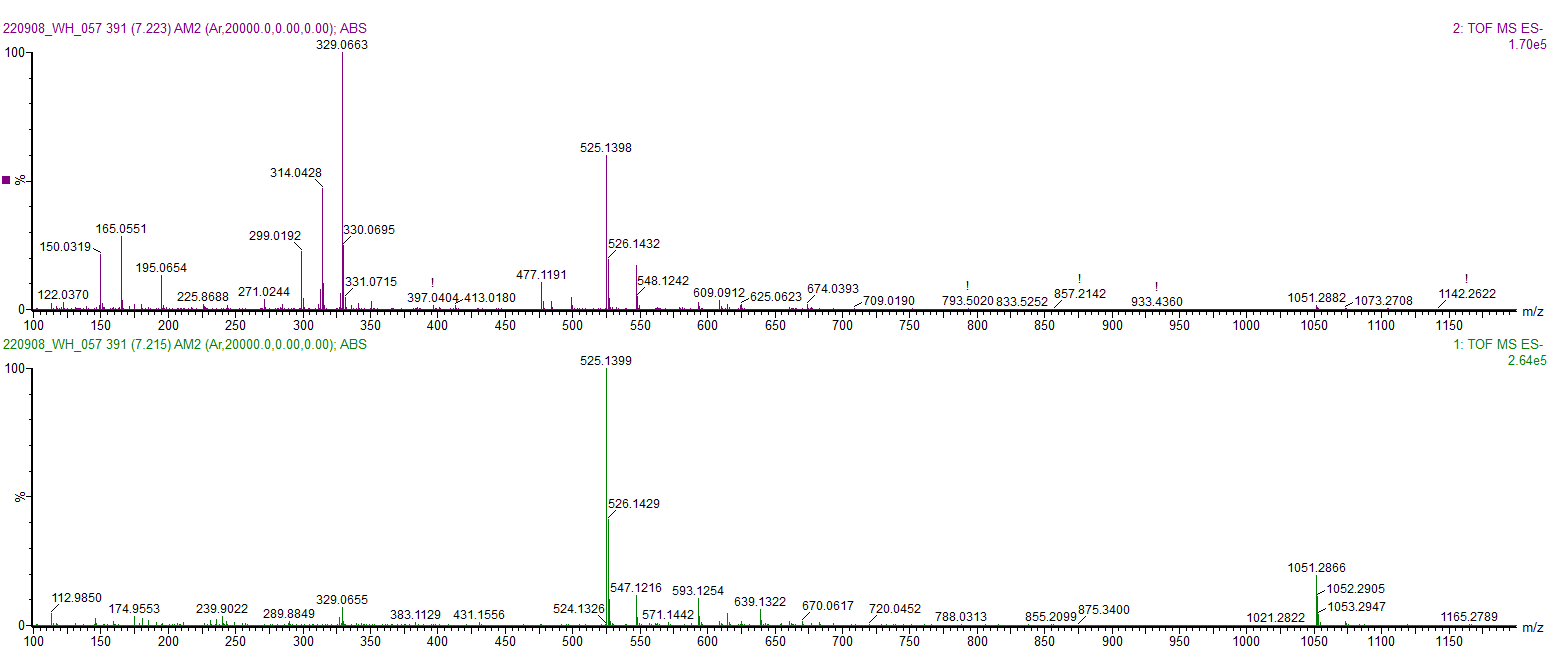
**

**Supplementary Figure 29.** ESI-QTof-MS spectrum of tricin-4'-*O*-(*threo*-*β*-guaiacylglyceryl) ether (peak 29).

**
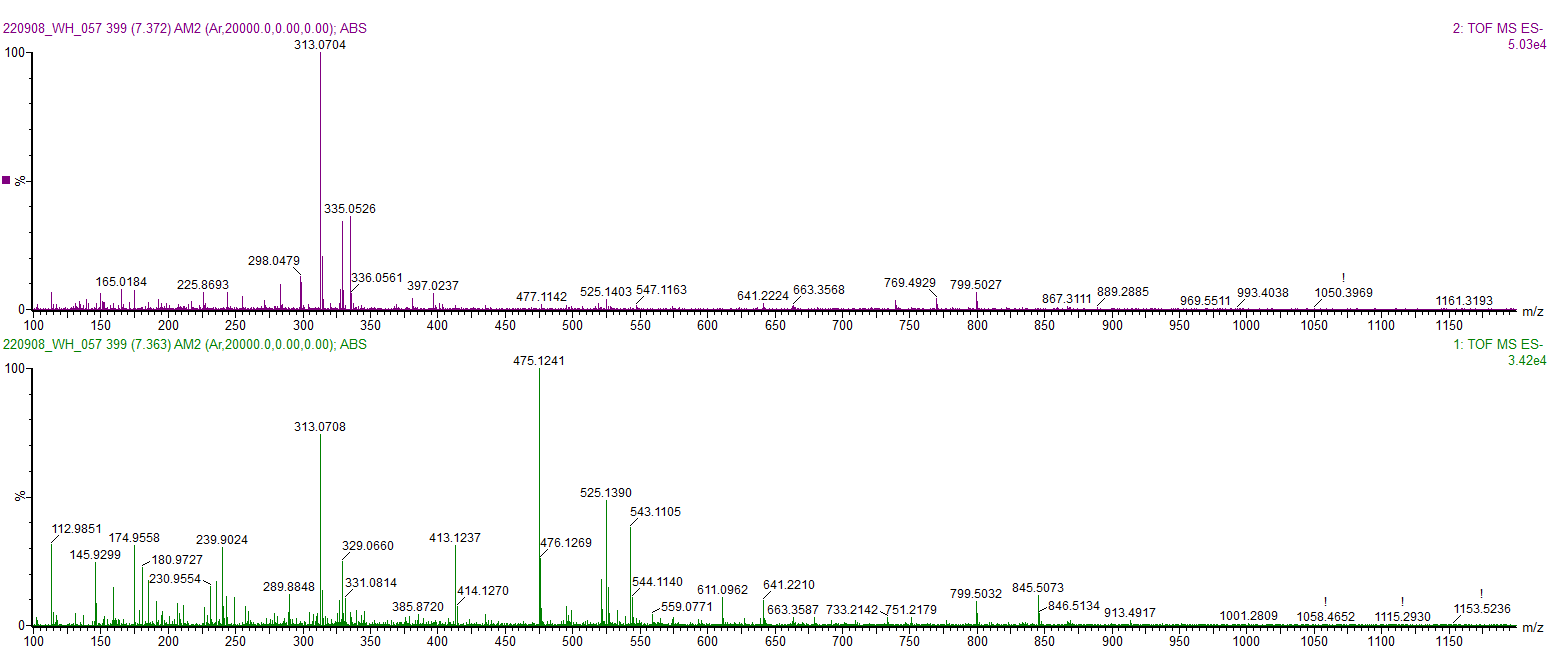
**

**Supplementary Figure 30.** ESI-QTof-MS spectrum of luteolin-3',4'-dimethylether-7-*O*-glucoside (peak 30).

**
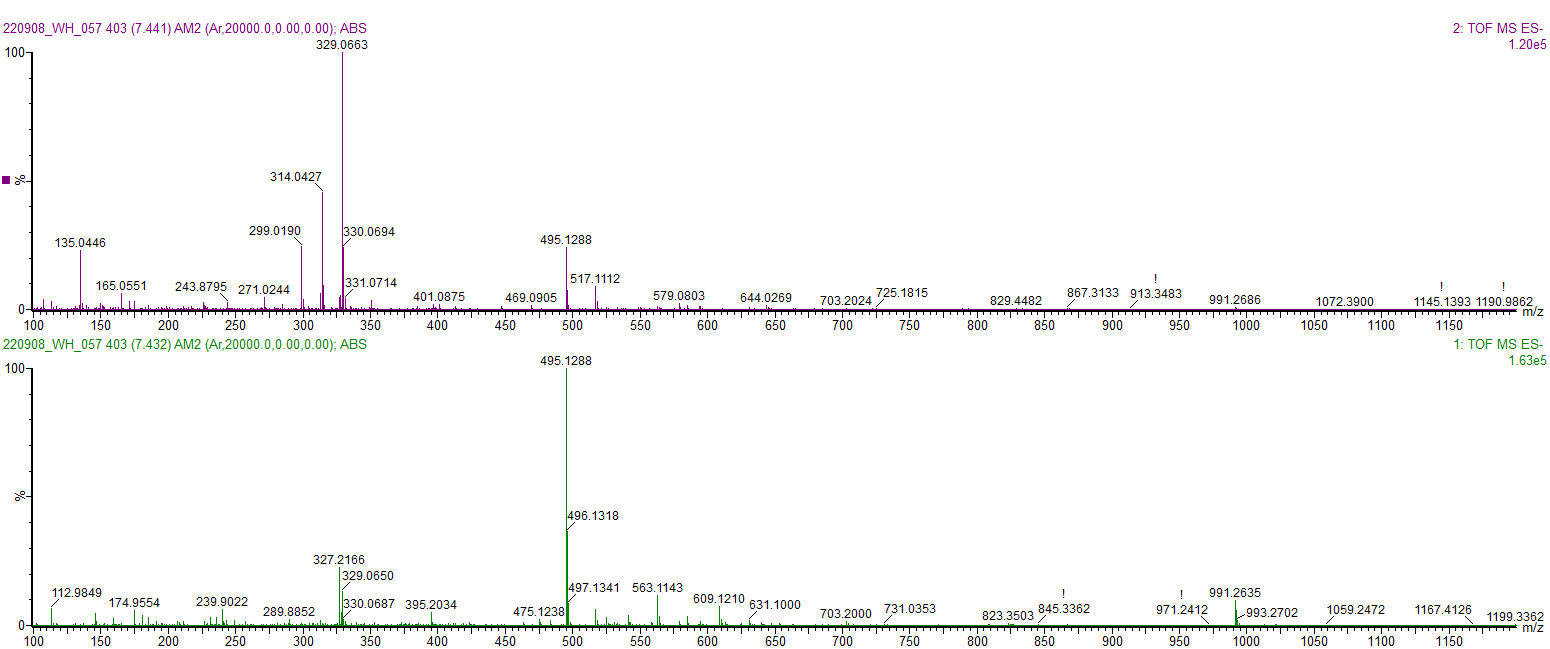
**

**Supplementary Figure 31.** ESI-QTof-MS spectrum of tricin-4'-*O*-(*erythro*-*β*-*p*-hydroxyphenylglyceryl) ether (peak 31).

**
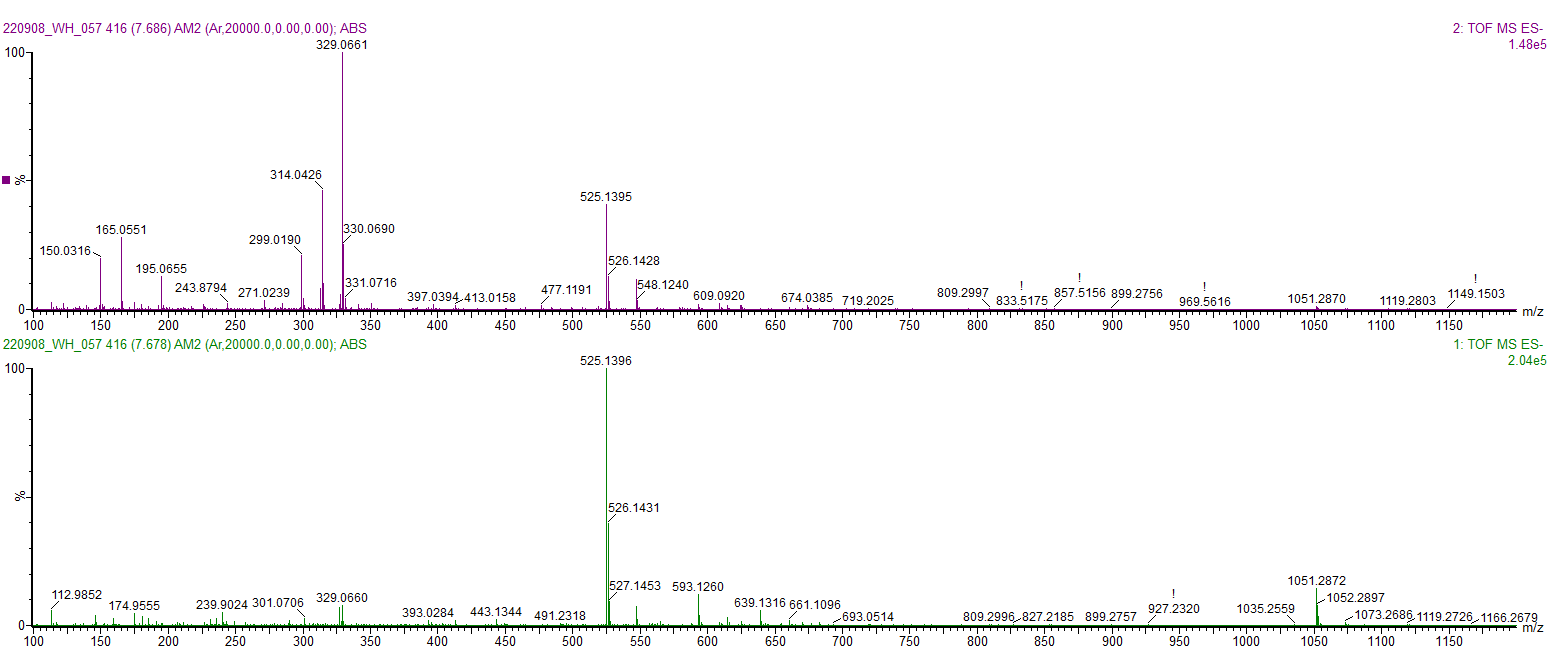
**

**Supplementary Figure 32.** ESI-QTof-MS spectrum of tricin-4'-*O*-(*erythro*-*β*-guaiacylglyceryl) ether (peak 32).

**
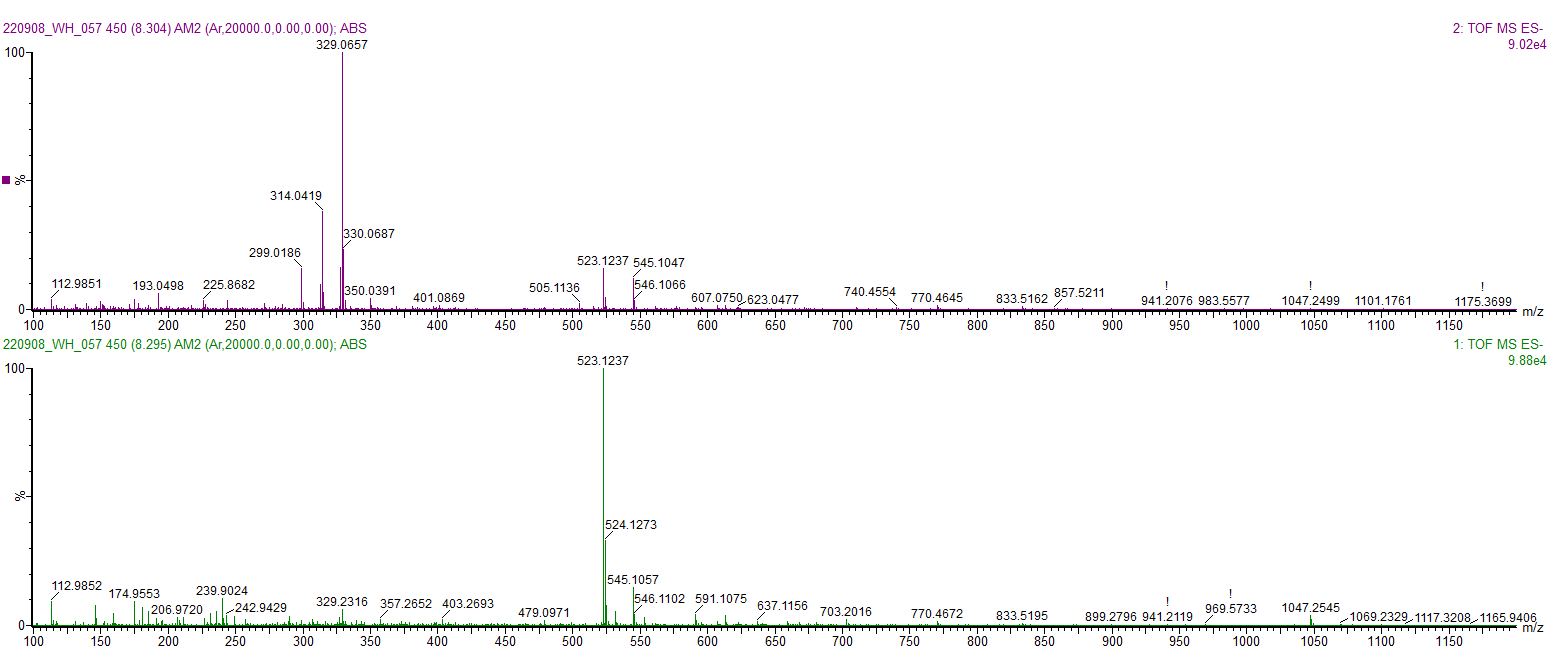
**

**Supplementary Figure 33.** ESI-QTof-MS spectrum of tricin-4'-*O*-(*C*-veratroylglycol) ether (peak 33).

**
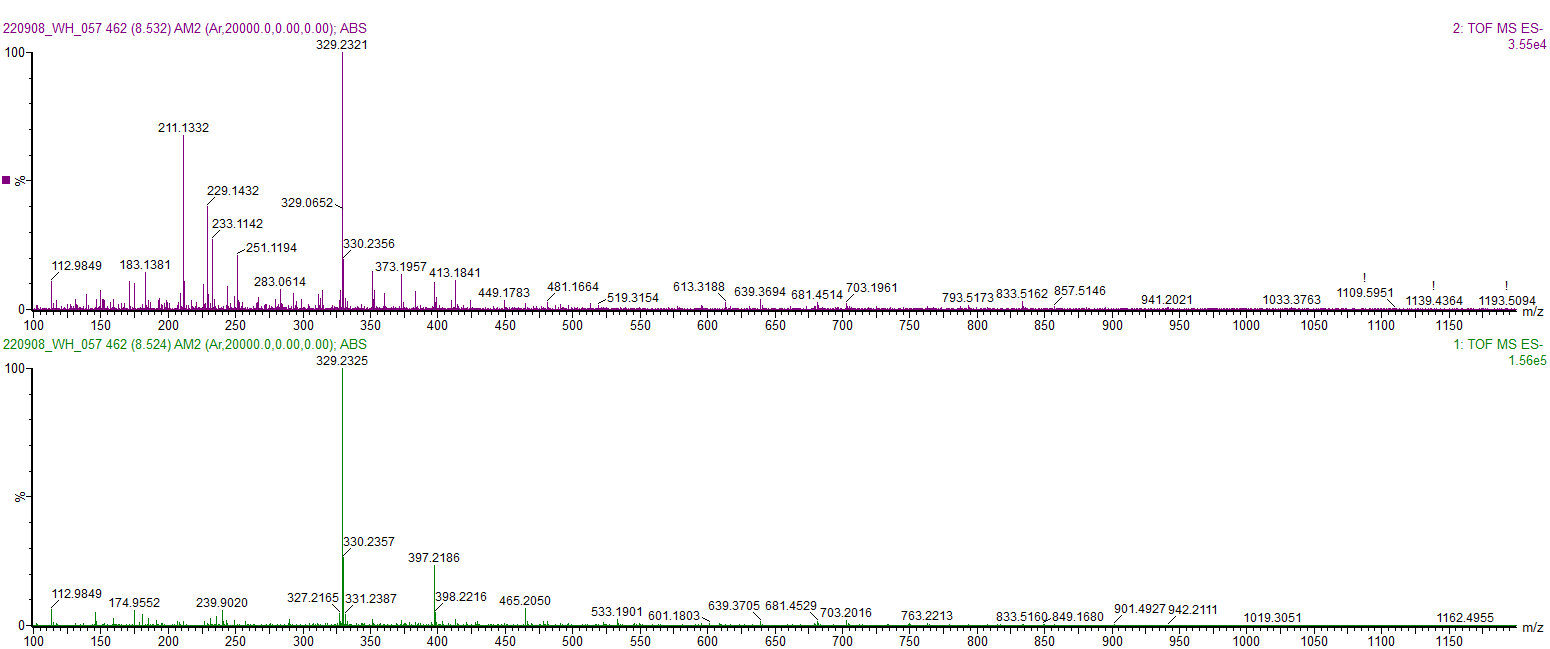
**

**Supplementary Figure 34.** ESI-QTof-MS spectrum of pinellic acid (isomer 1) (peak 34).

**
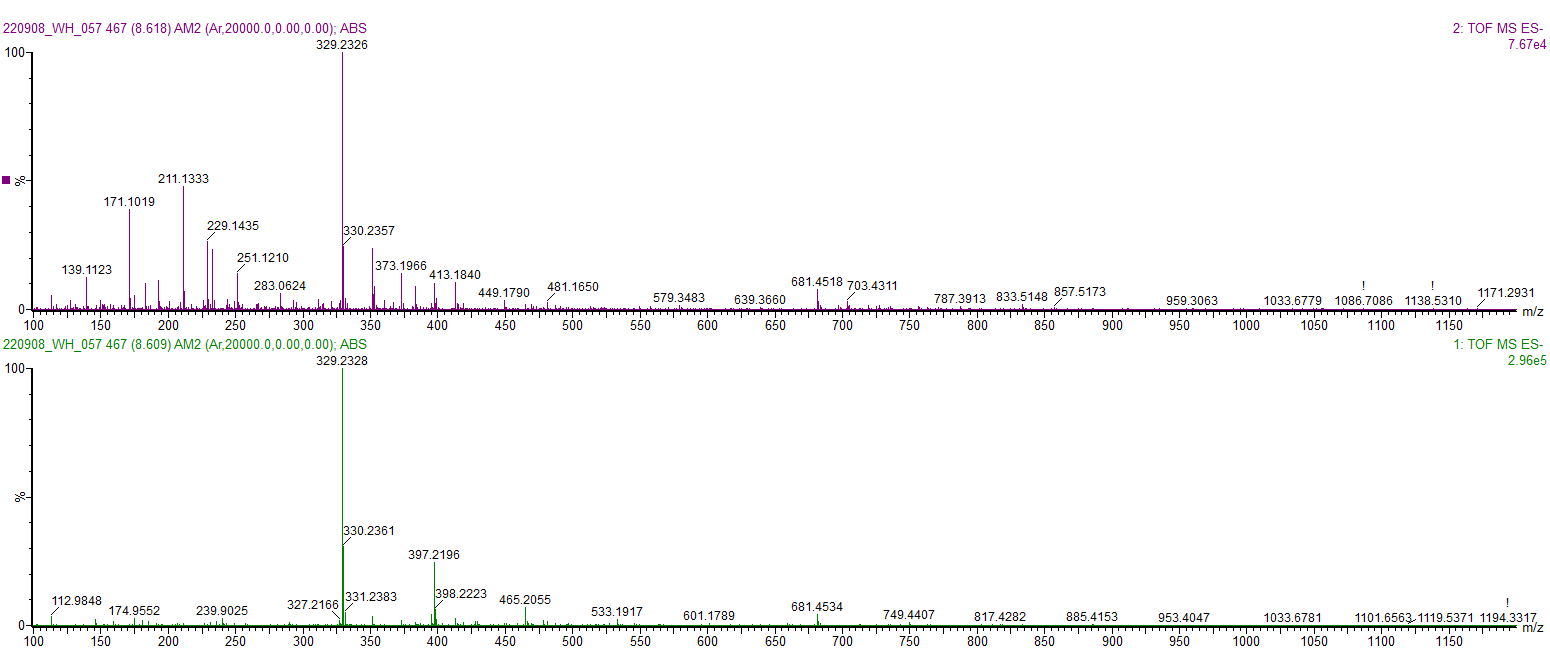
**

**Supplementary Figure 35.** ESI-QTof-MS spectrum of pinellic acid (isomer 2) (peak 35).

**
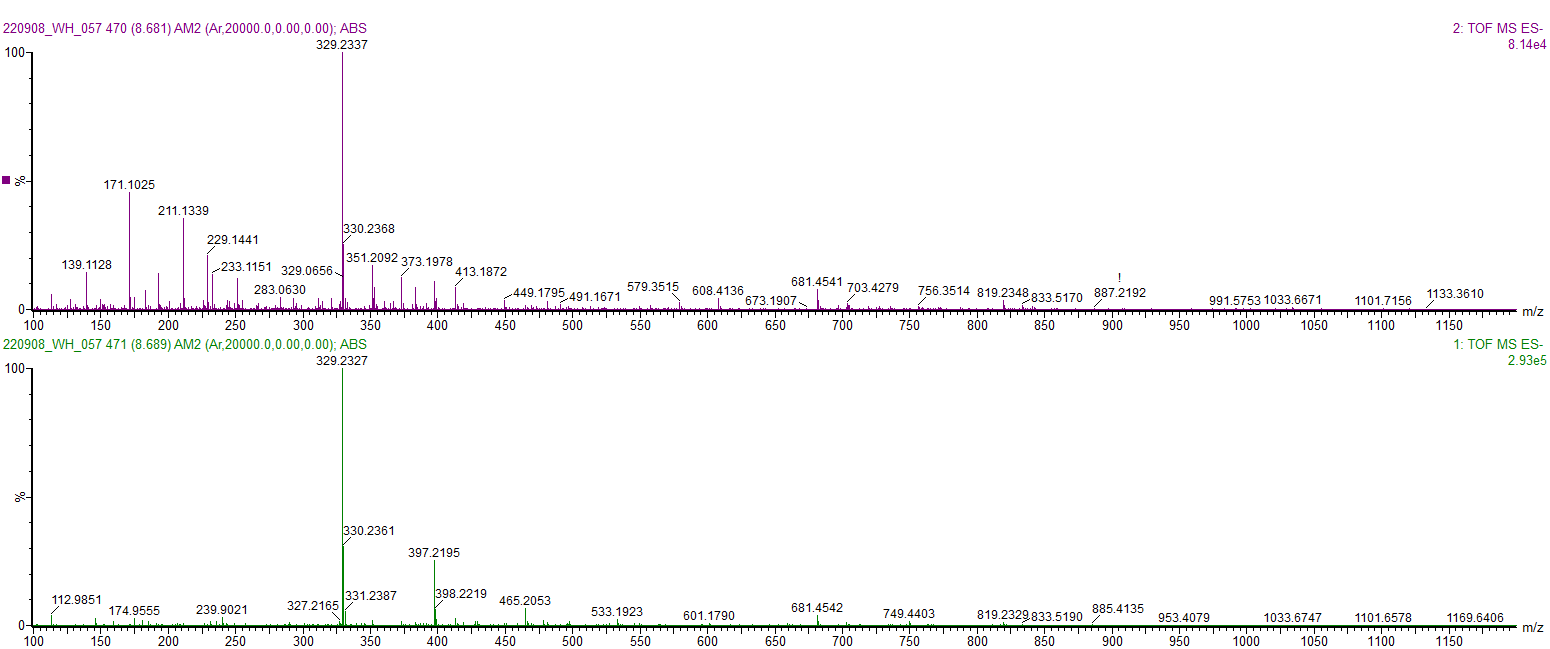
**

**Supplementary Figure 36.** ESI-QTof-MS spectrum of pinellic acid (isomer 3) (peak 36).

**
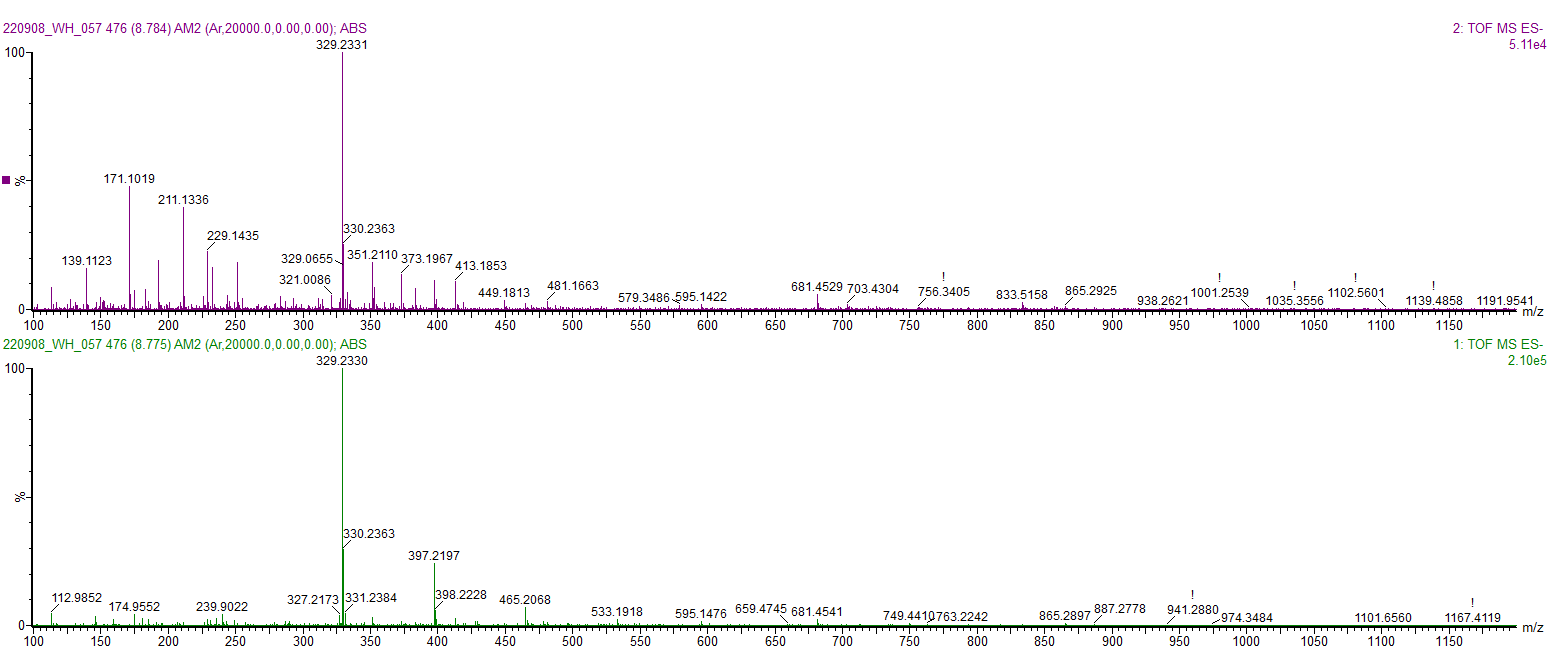
**

**Supplementary Figure 37.** ESI-QTof-MS spectrum of pinellic acid (isomer 4) (peak 37).

**
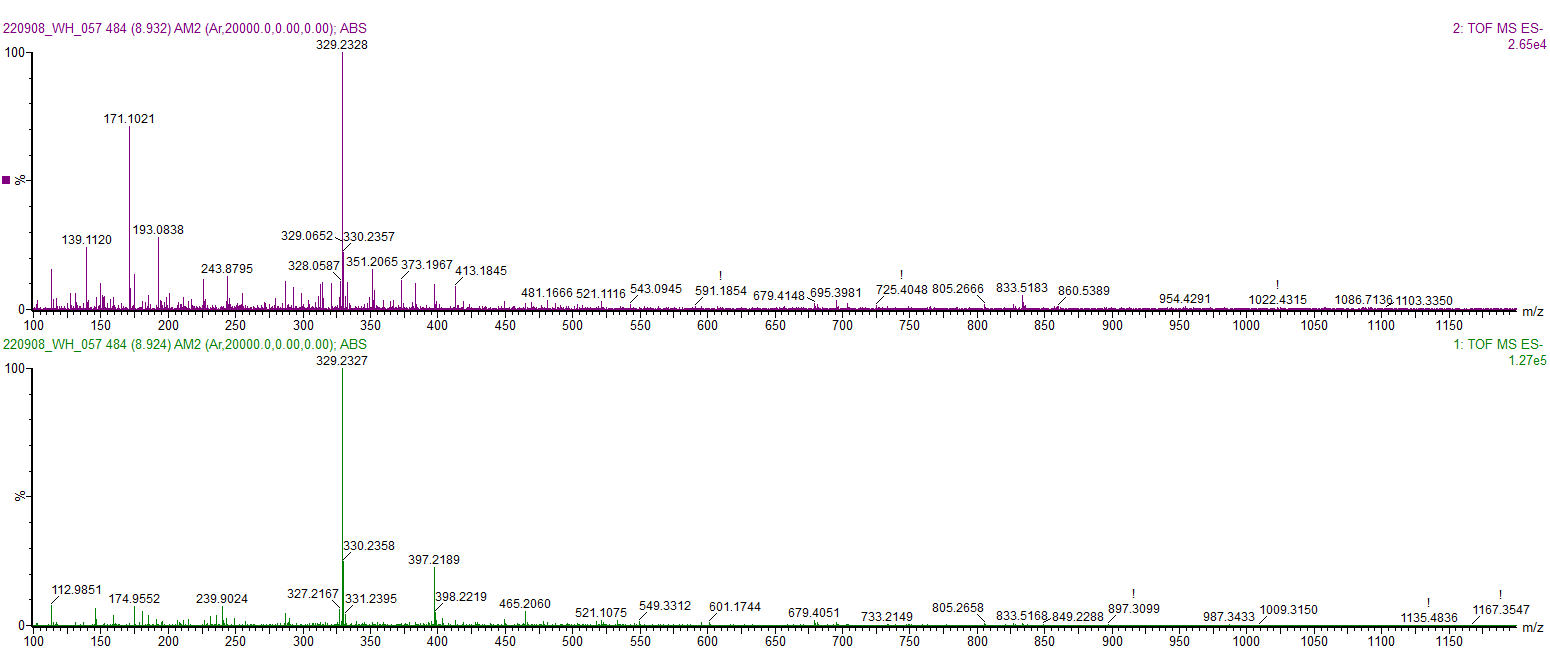
**

**Supplementary Figure 38.** ESI-QTof-MS spectrum of 5,8,12-trihydroxy-trans-9-octadecenoic acid (peak 38).

**
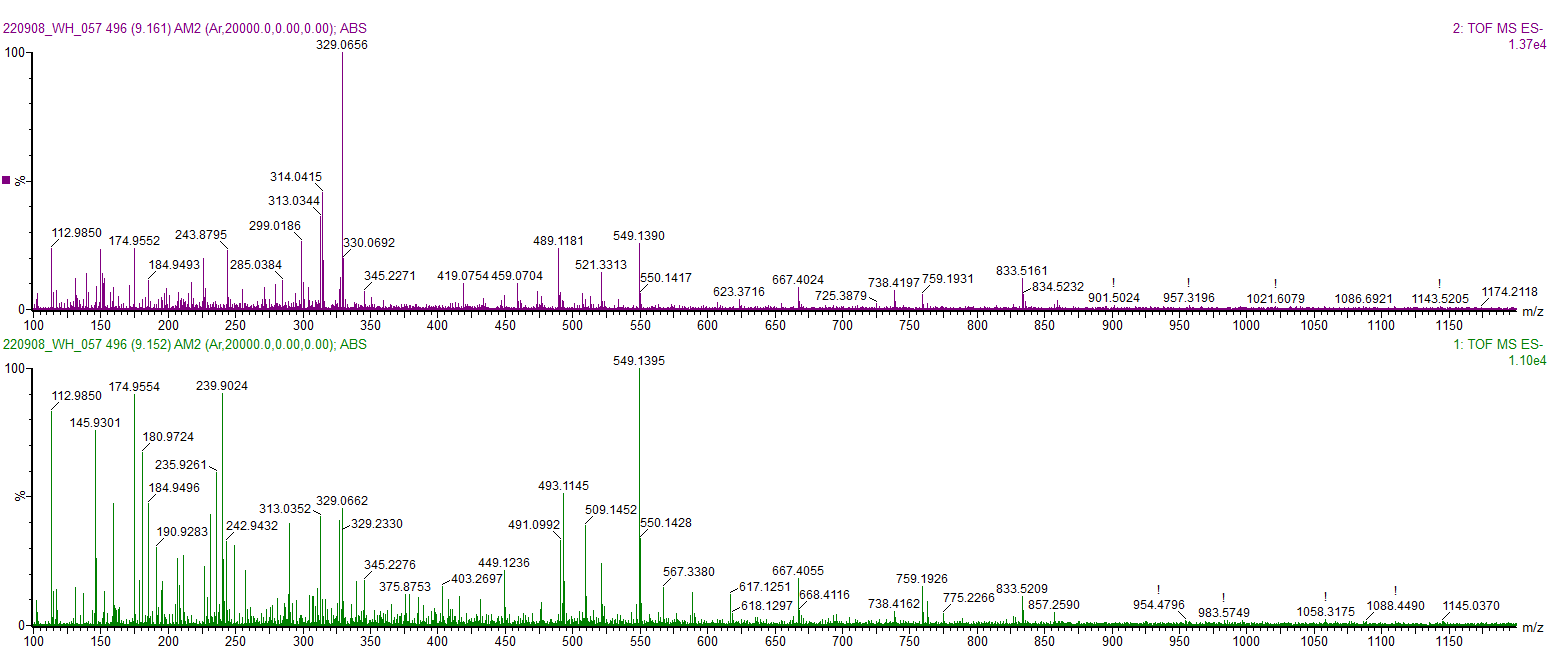
**

**Supplementary Figure 39.** ESI-QTof-MS spectrum of tricin-4'-*O*-[*threo*-*β*-*p*-hydroxyphenyl (7"-*O*-methyl)-glyceryl] ether (peak 39).

**
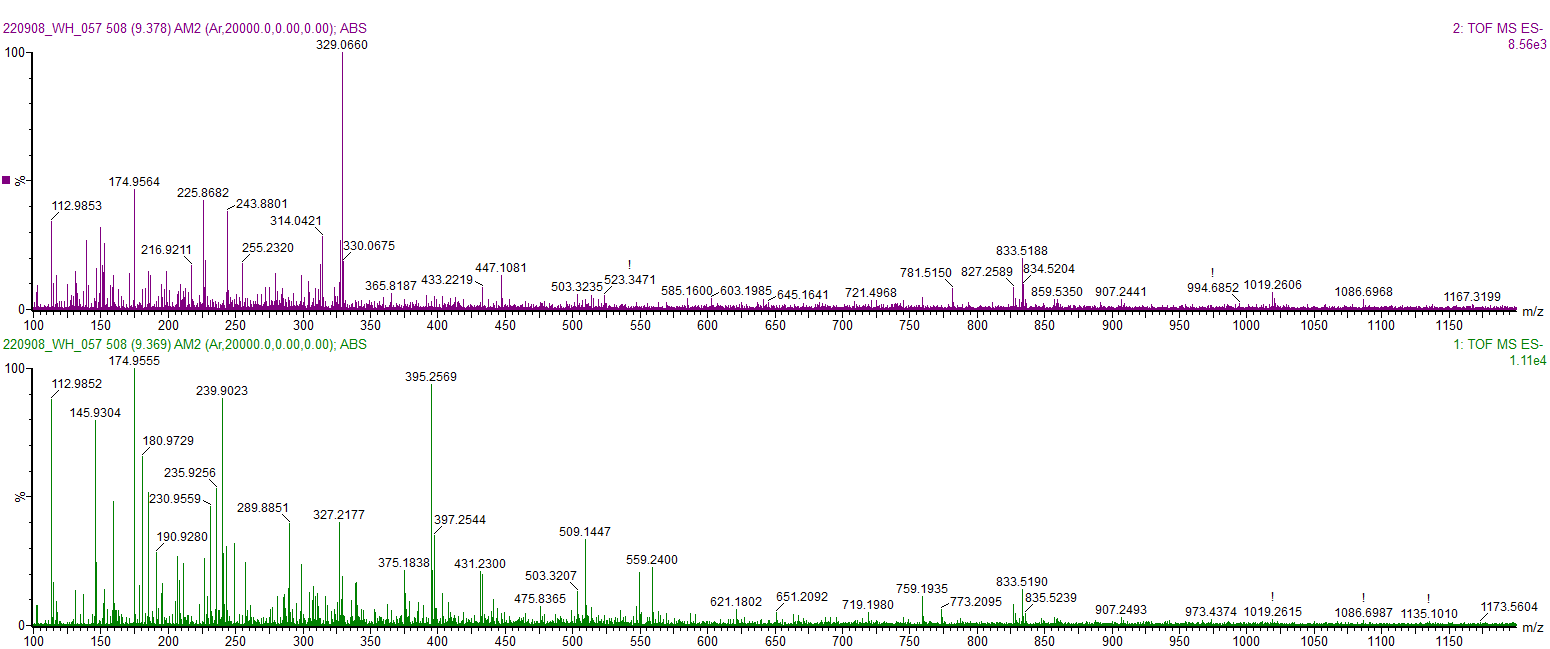
**

**Supplementary Figure 40.** ESI-QTof-MS spectrum of tricin-4'-*O*-[*erythro*-*β*-*p*-hydroxyphenyl-(7"-*O*-methyl)-glyceryl] ether (peak 40).

**
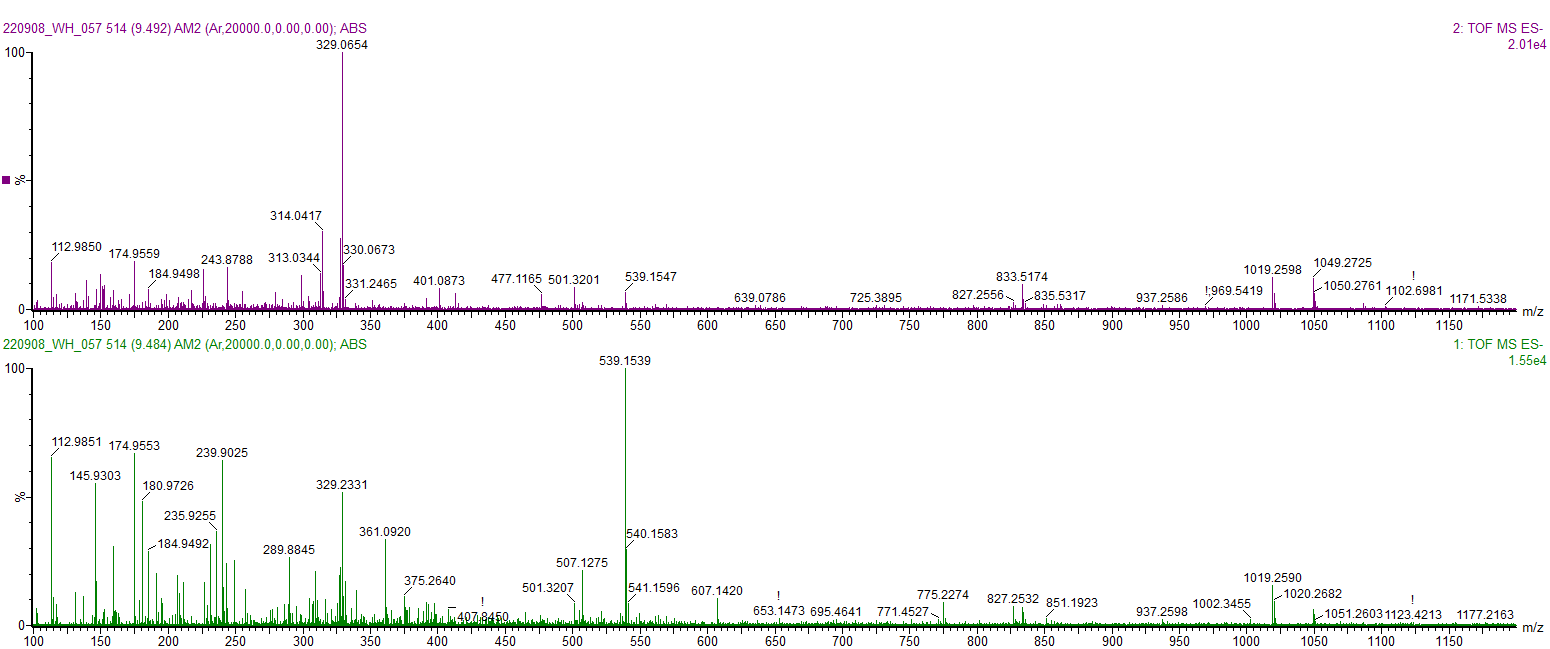
**

**Supplementary Figure 41.** ESI-QTof-MS spectrum of tricin-4'-*O*-[*threo*-*β*-guaiacyl-(7"-*O*-methyl)-glyceryl] ether (peak 41).

**
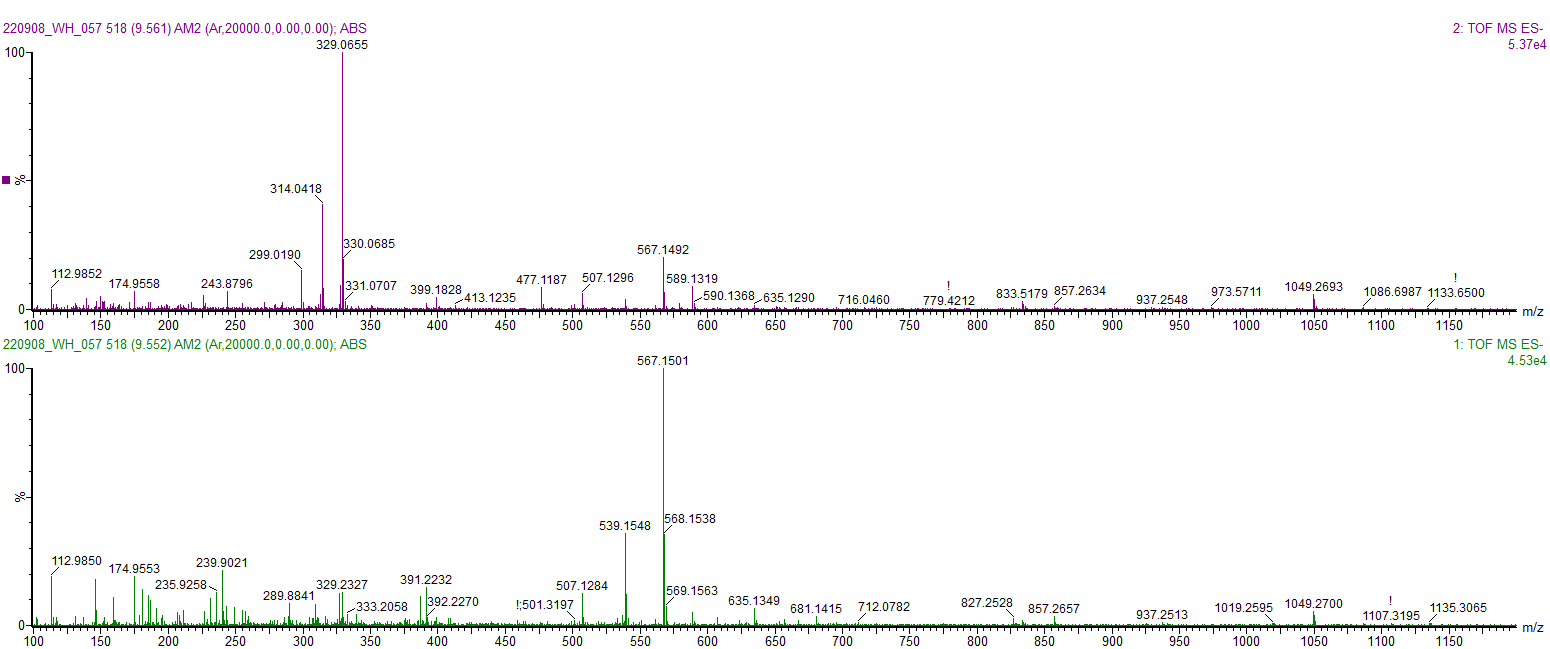
**

**Supplementary Figure 42.** ESI-QTof-MS spectrum of tricin-4'-*O*-[ery*thro-β*-guaiacyl-(7"-*O*-methyl)-glyceryl] ether (peak 42).

**
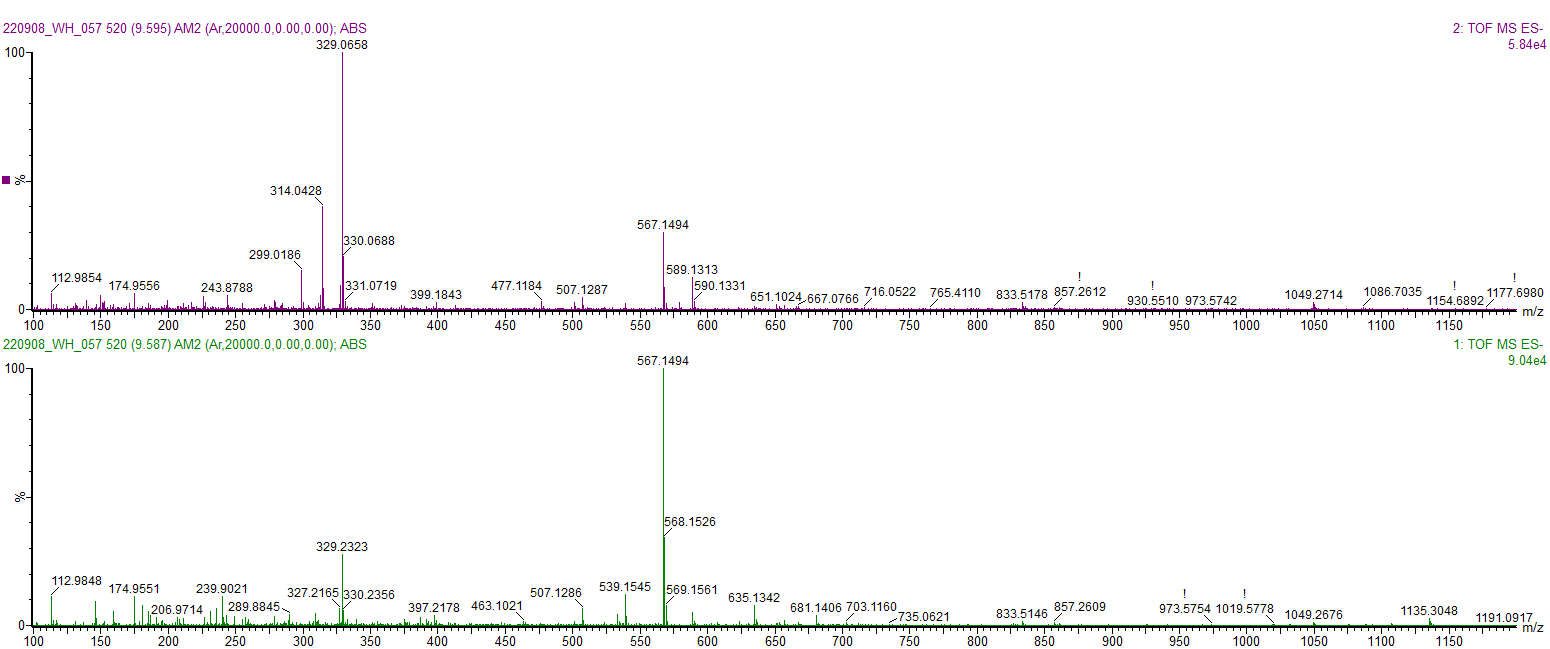
**

**Supplementary Figure 43.** ESI-QTof-MS spectrum of tricin-4'-*O*-[*erythro*-*β*-guaiacyl-(9"-*O*-acetyl)-glyceryl] ether (peak 43).

**
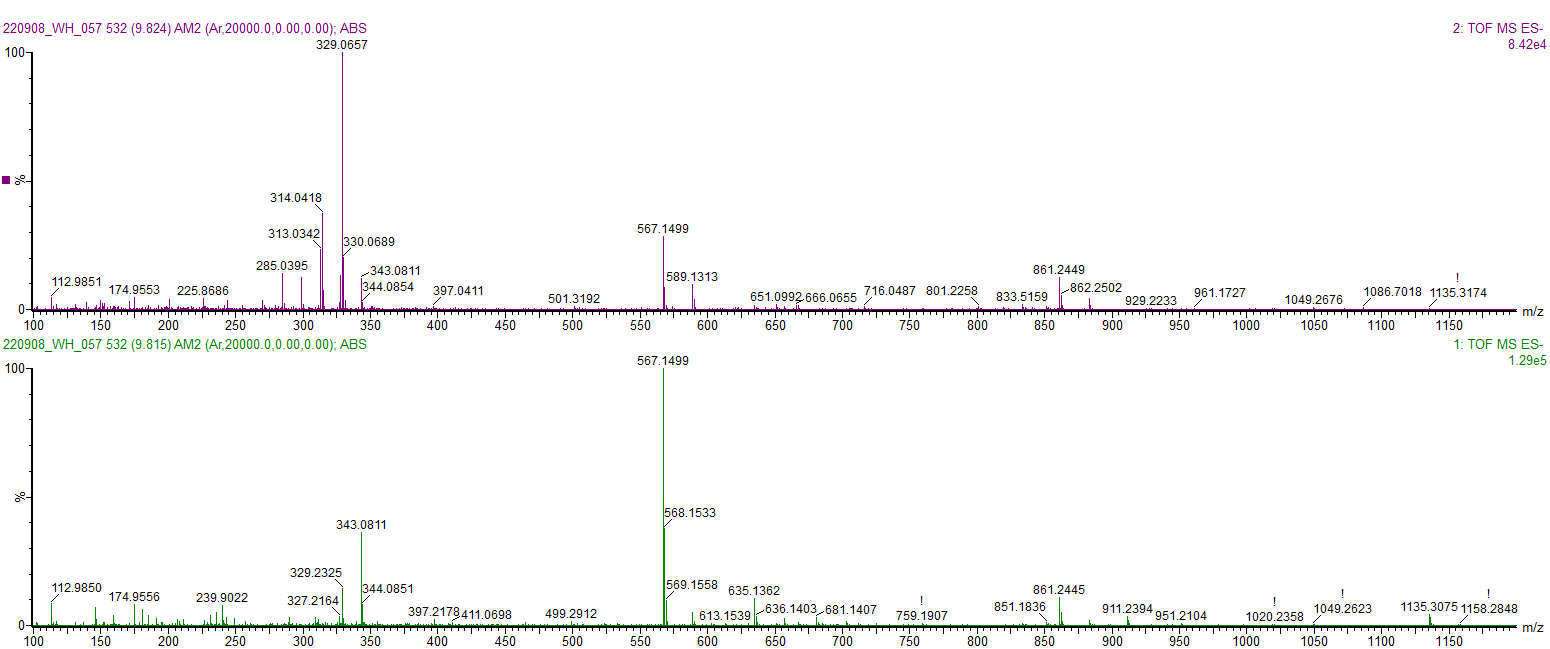
**

**Supplementary Figure 44.** ESI-QTof-MS spectrum of tricin-4'-*O*-[*threo*-*β*-guaiacyl-(9"-*O*-acetyl)-glyceryl] ether (peak 44).

**
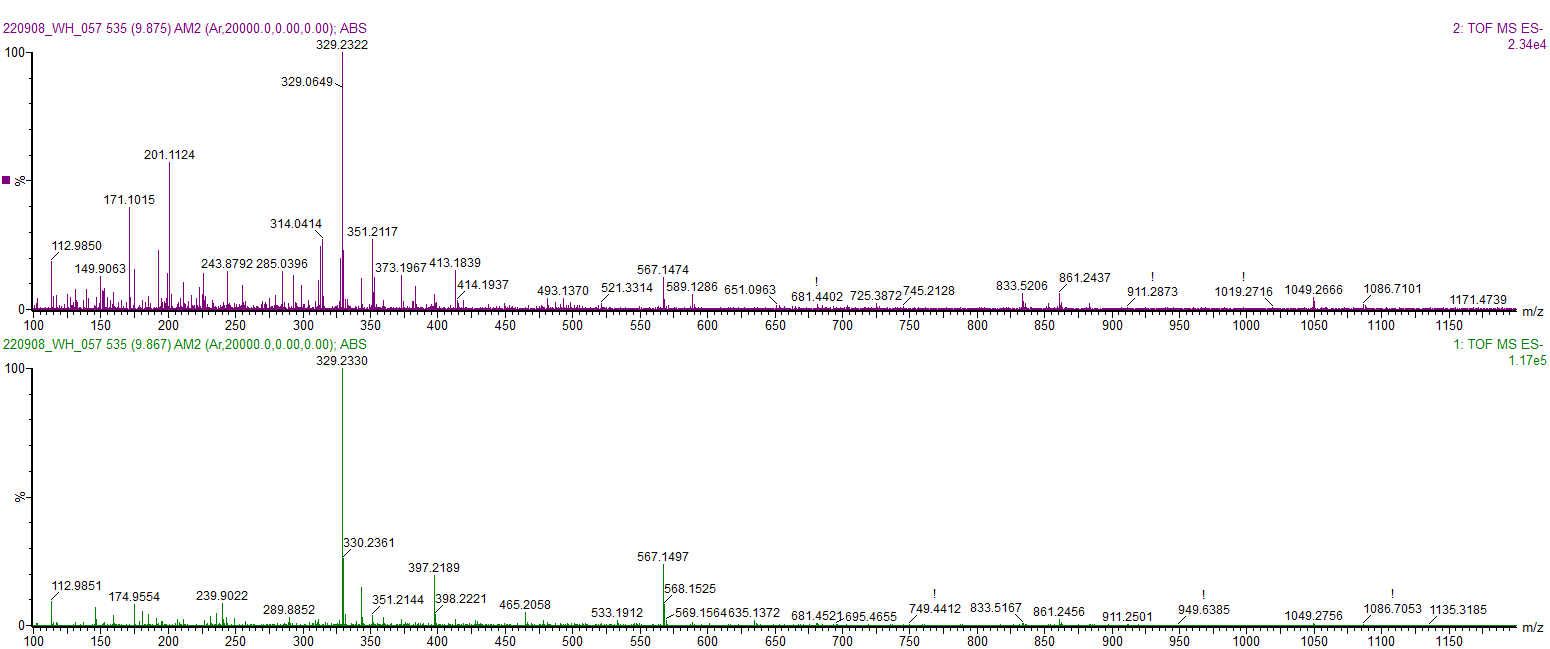
**

**Supplementary Figure 45.** ESI-QTof-MS spectrum of trihydroxy octadecenoic acid (isomer 1) (peak 45).

**
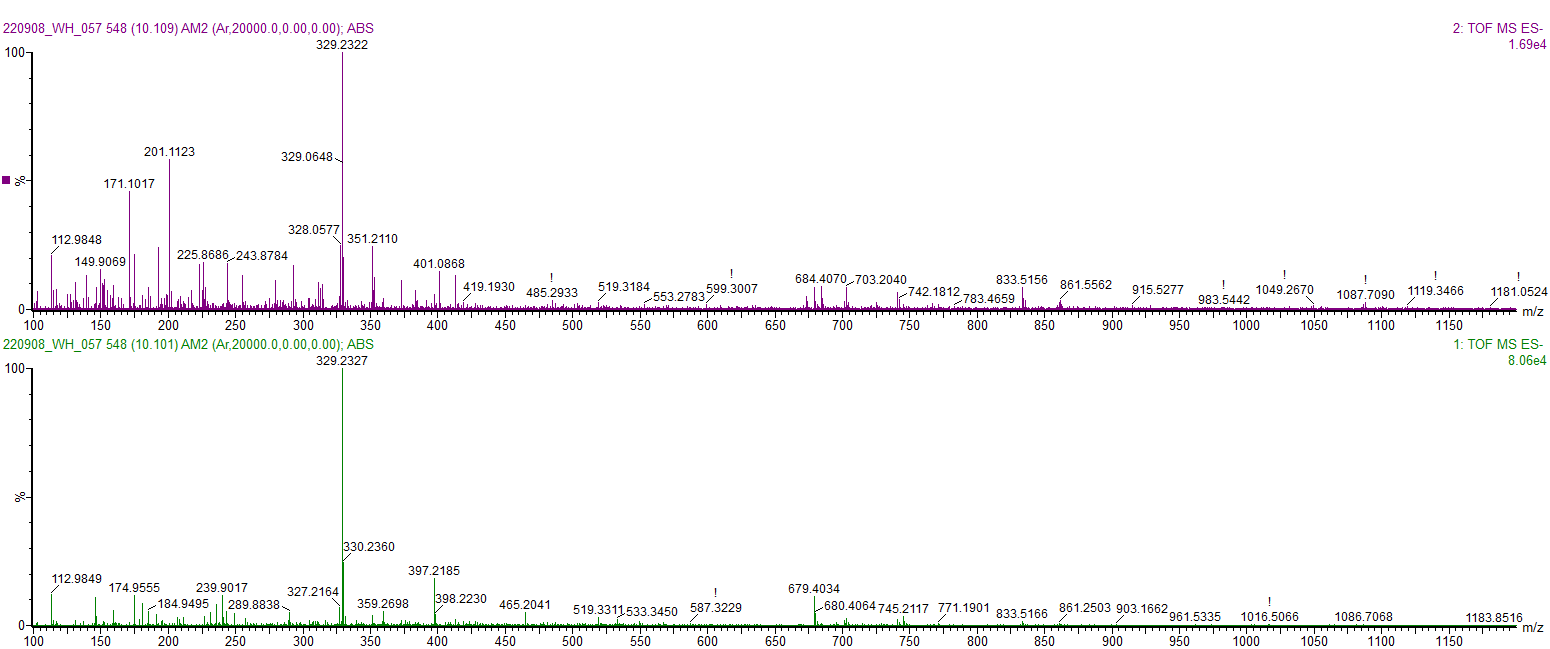
**

**Supplementary Figure 46.** ESI-QTof-MS spectrum of trihydroxy octadecenoic acid (isomer 2) (peak 46).

**
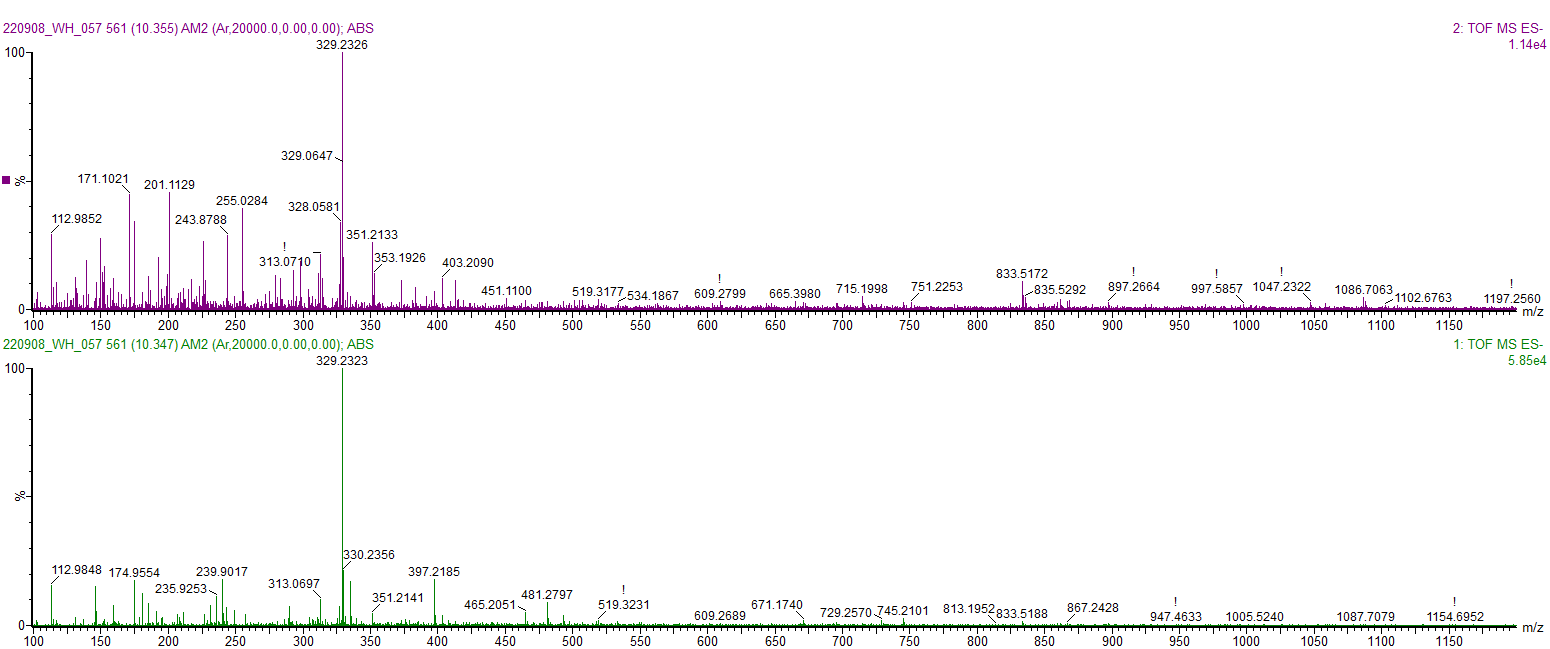
**

**Supplementary Figure 47.** ESI-QTof-MS spectrum of trihydroxy octadecenoic acid (isomer 3) (peak 47).

**
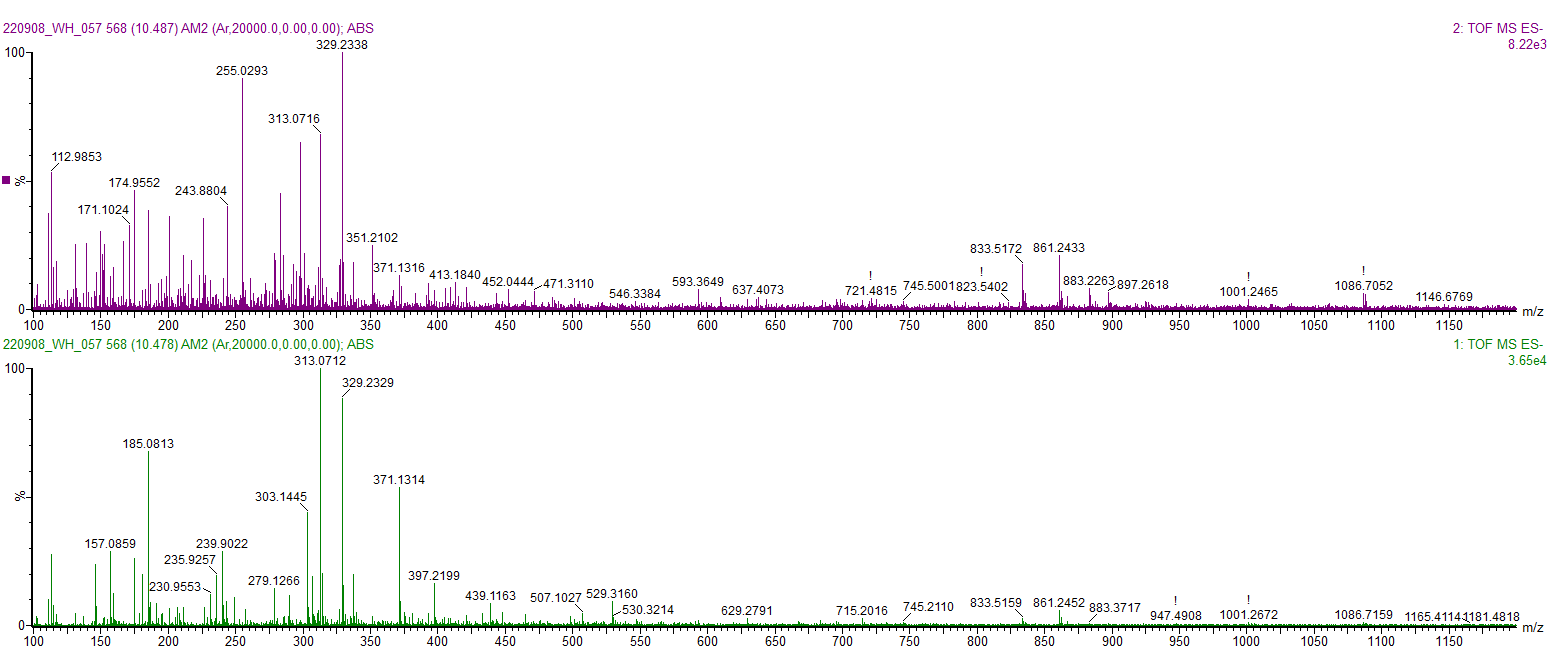
**

**Supplementary Figure 48.** ESI-QTof-MS spectrum of trihydroxy octadecenoic acid (isomer 4) (peak 48).

**
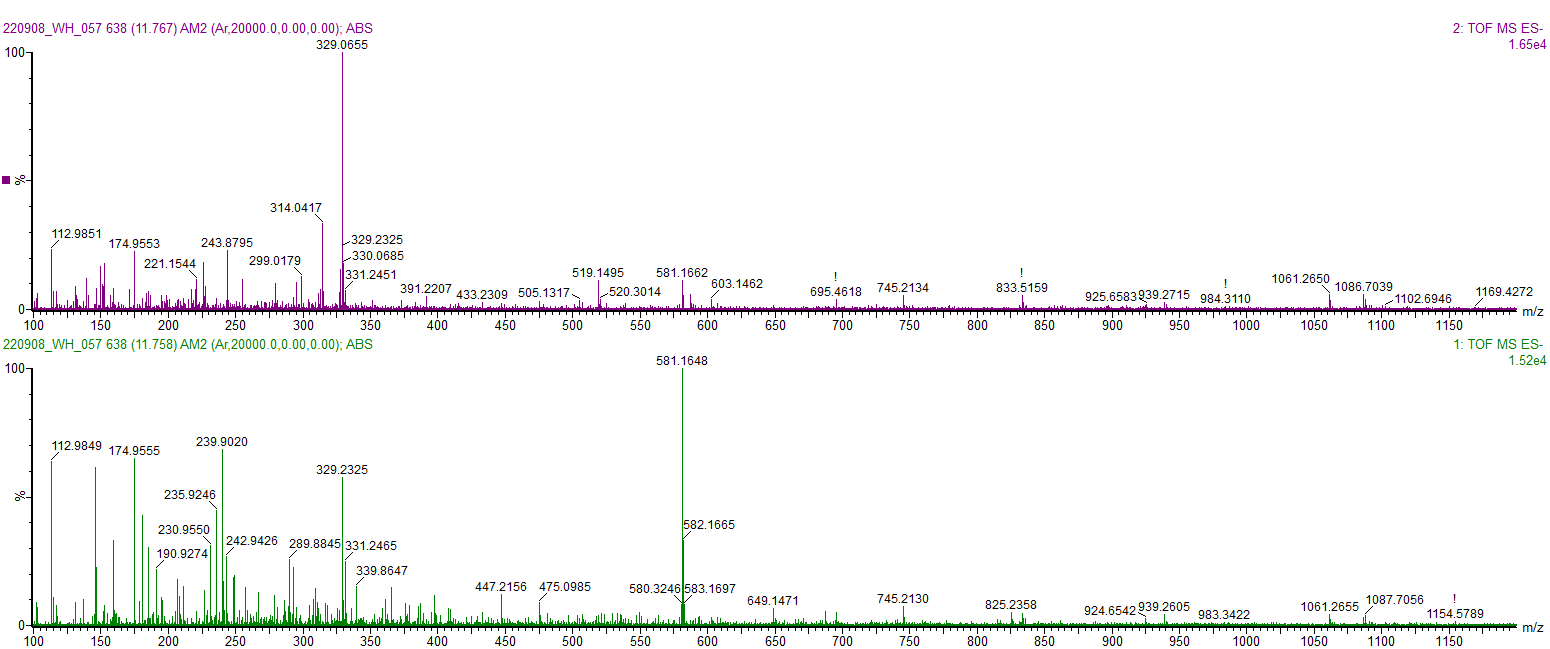
**

**Supplementary Figure 49.** ESI-QTof-MS spectrum of tricin-4'-*O*-[*threo*-*β*-guaiacyl-(7"-*O*-methyl-9"-*O*-acetyl)-glyceryl] ether (peak 49).

**
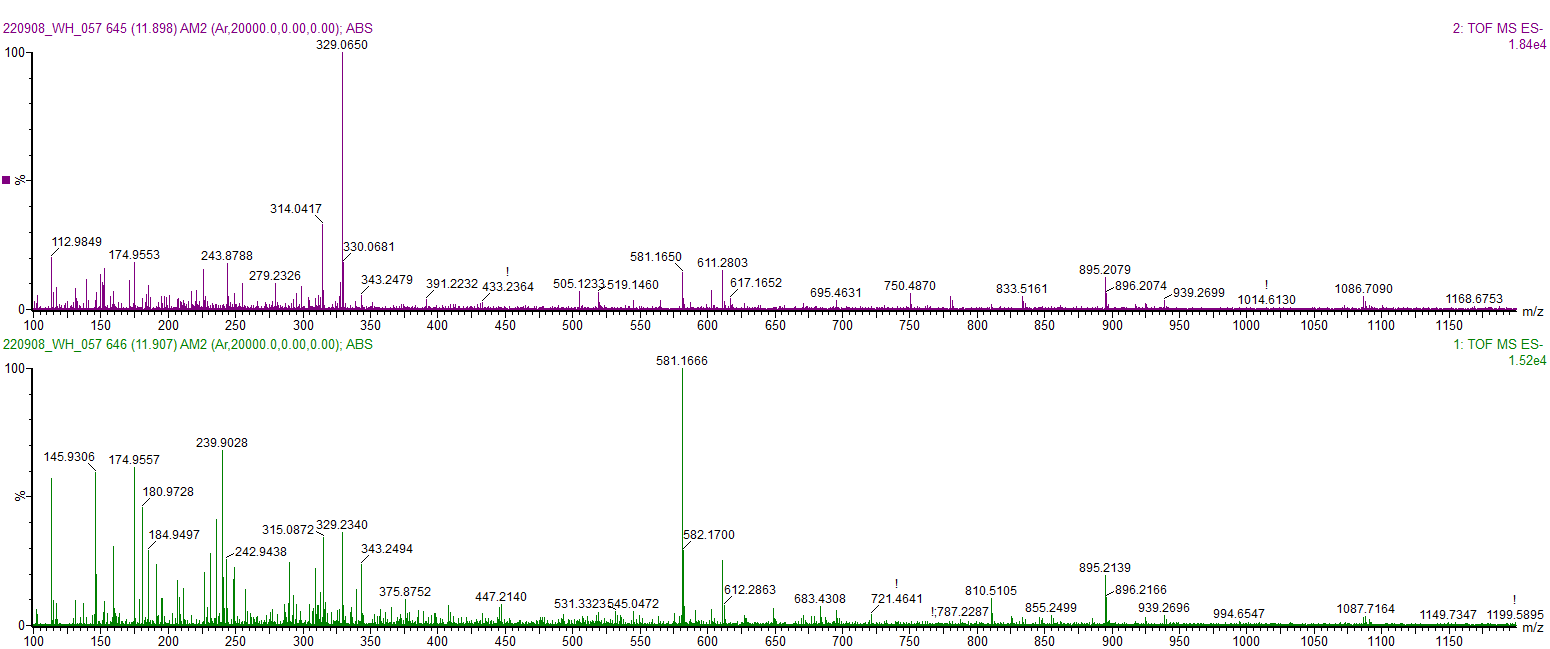
**

**Supplementary Figure 50.** ESI-QTof-MS spectrum of tricin-4'-*O*-[*erythro*-*β*-guaiacyl-(7"-*O*-methyl-9"-*O*-acetyl)-glyceryl] ether (peak 50).

**
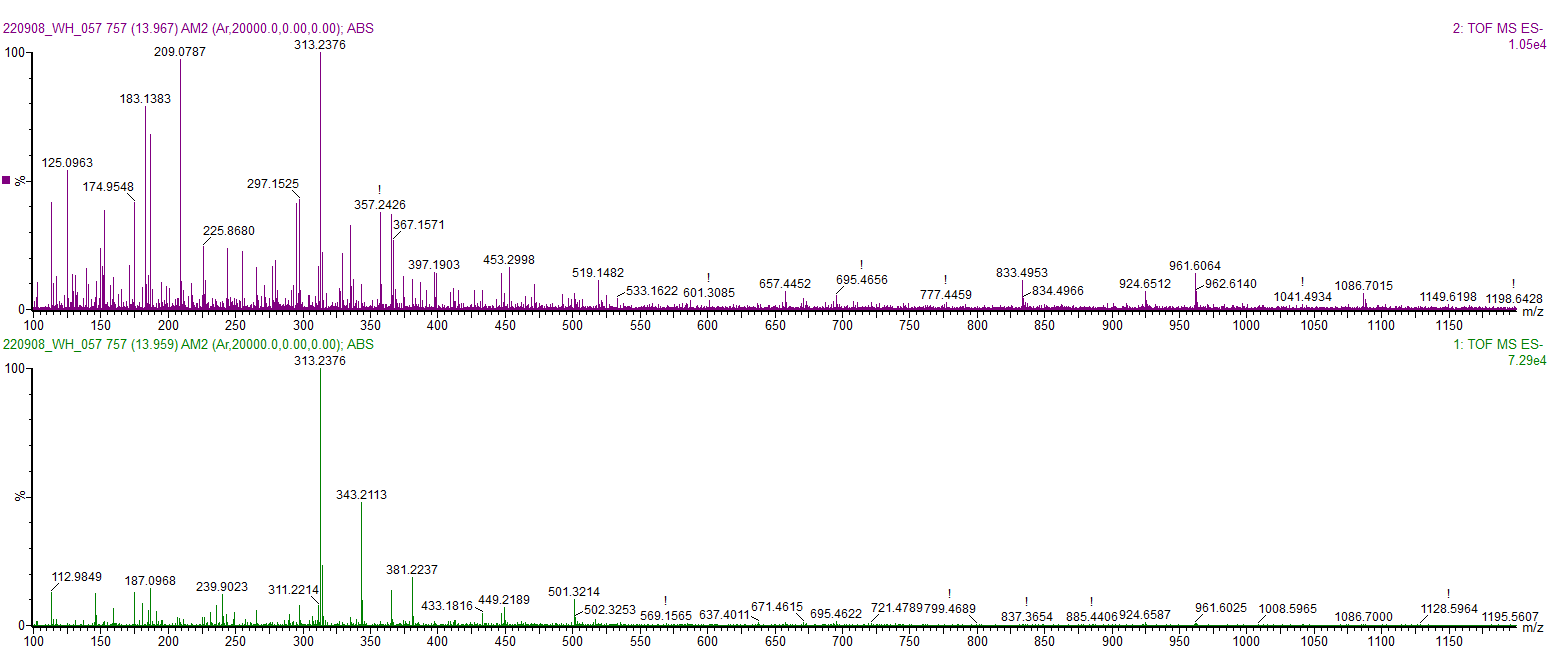
**

**Supplementary Figure 51.** ESI-QTof-MS spectrum of 12,13-dihydroxy-9*Z*-octadecenoic acid (peak 51).

**
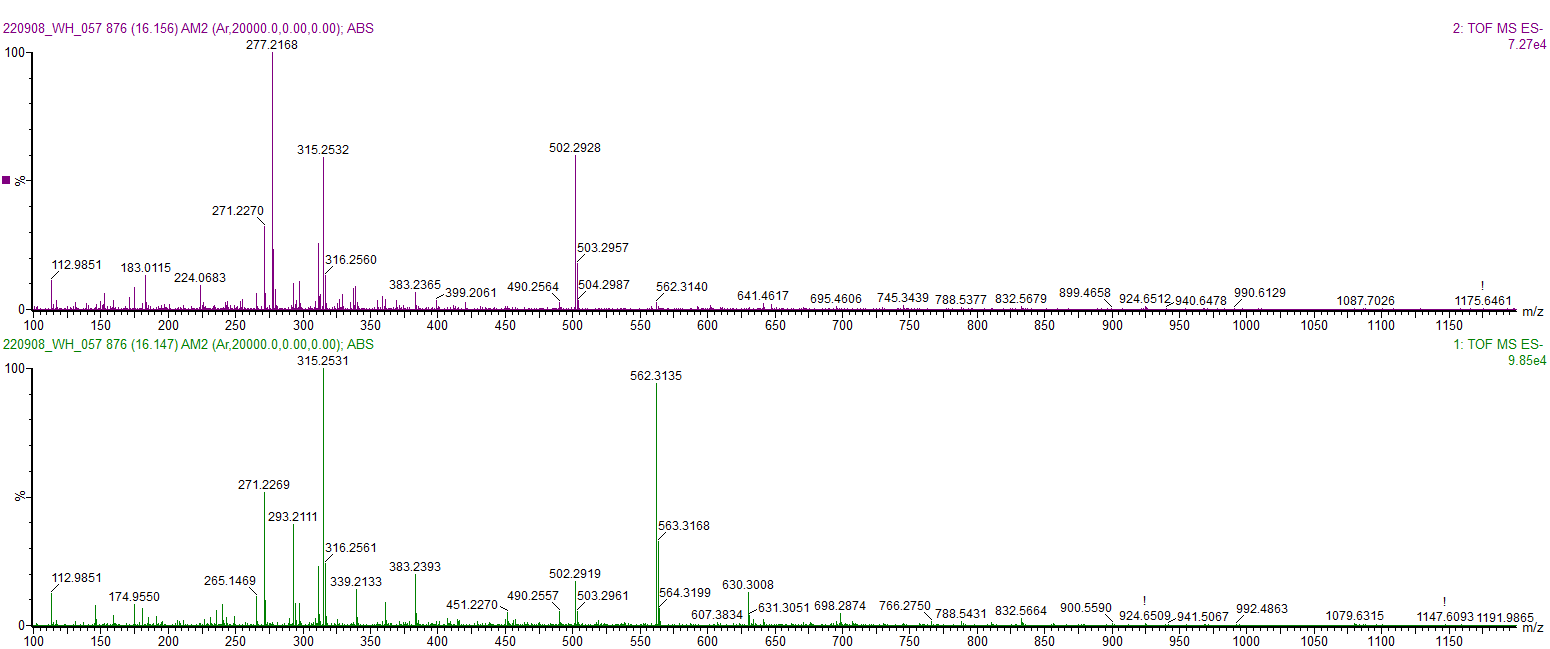
**

**Supplementary Figure 52.** ESI-QTof-MS spectrum of 1-(9*Z*,12*Z*,15*Z*-octadecadienoyl)-*sn*-glycero-3-phosphocholine (peak 52).

**
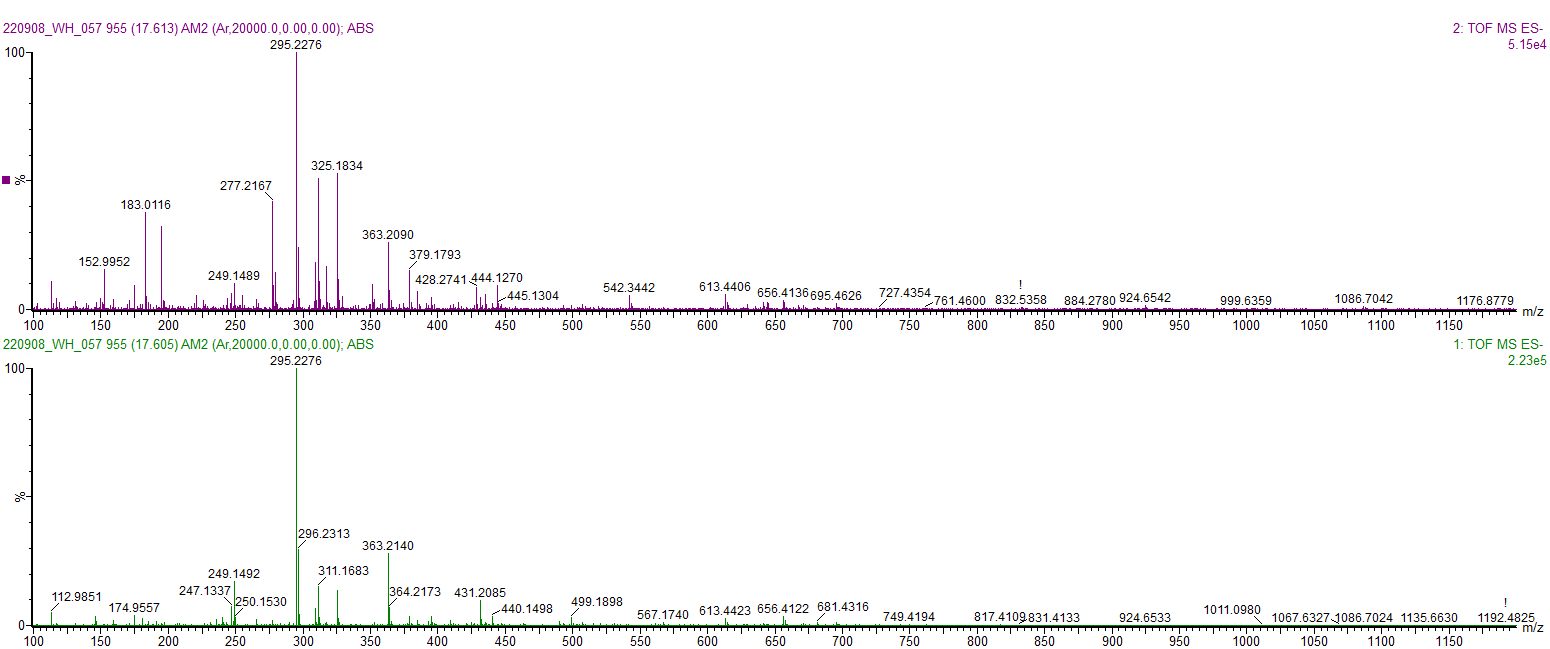
**

**Supplementary Figure 53.** ESI-QTof-MS spectrum of 9*E*,11*E*-13-hydroxyoctadecadienoic acid (peak 53).

**
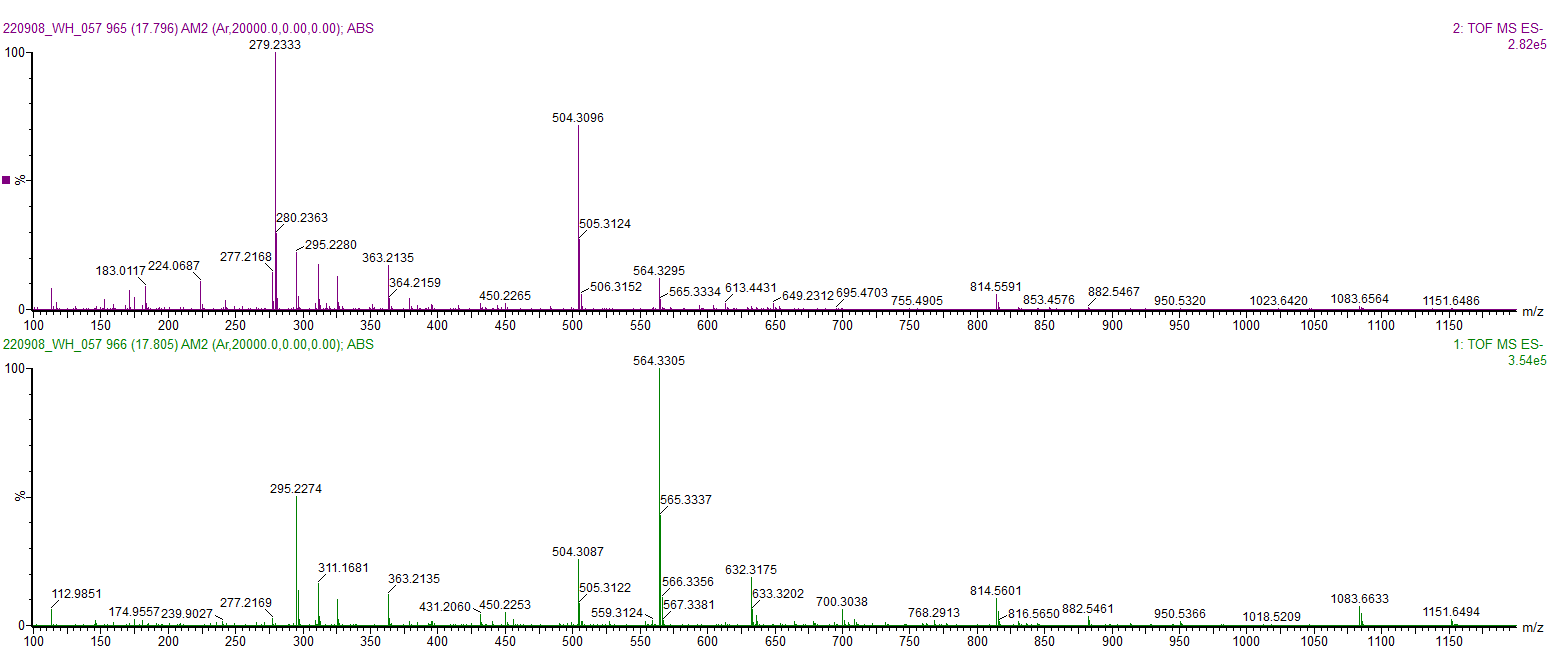
**

**Supplementary Figure 54.** ESI-QTof-MS spectrum of 1-(9*Z*,12*Z*-octadecadienoyl)-*sn*-glycero-3-phosphocholine (peak 54).

**
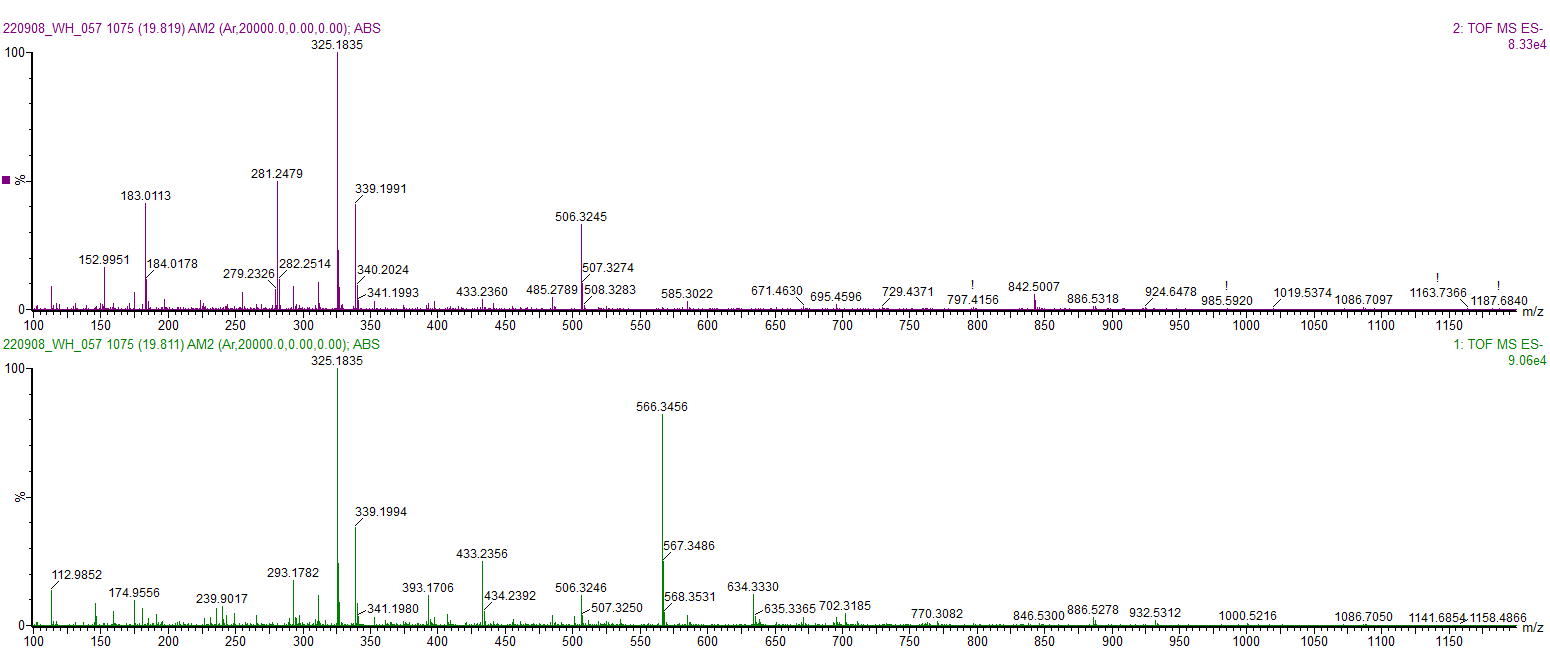
**

**Supplementary Figure 55.** ESI-QTof-MS spectrum of 1-(9*Z*-octadecenoyl)-*sn*-glycero-3-phosphocholine (peak 55).

**
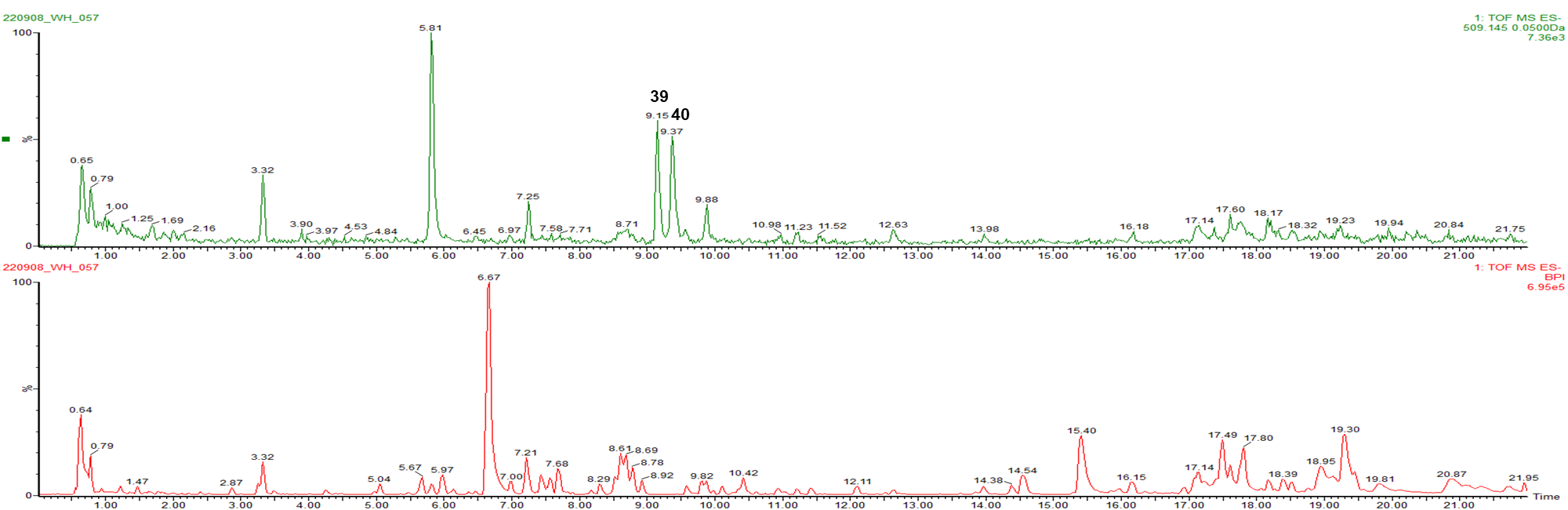
**

**
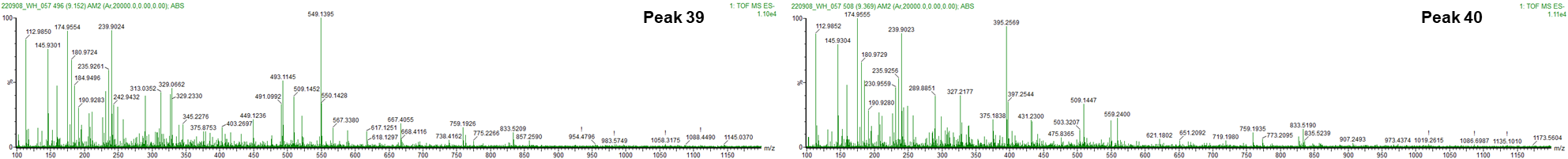
**

**Supplementary Figure 56.** The extracted ion chromatogram for *m/z* 509.145 and MS spectra of peaks 39 and 40.

**
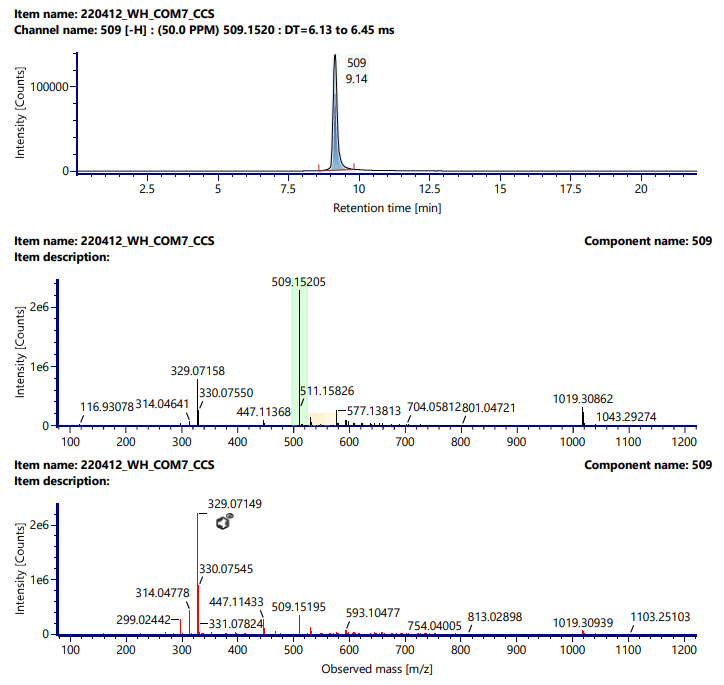
**

**
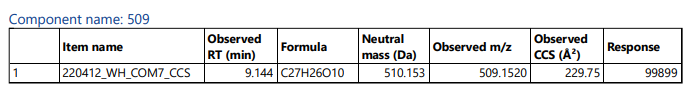
**

**Supplementary Figure 57.** High definition MS^E^ spectrum and collision cross section value (CCS, Å^2^) of 4'-*O*-[*threo*-*β*-*p*-hydroxyphenyl-(7''-*O*-methyl)-glyceryl] ether (peak 39).


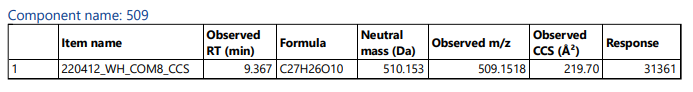

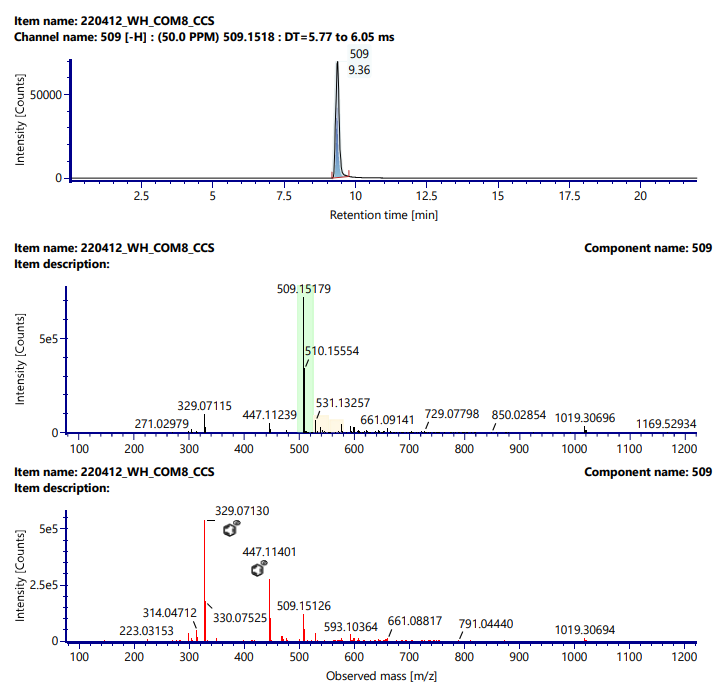


**Supplementary Figure 58.** High definition MS^E^ spectrum and collision cross section value (CCS, Å^2^) of 4'-*O*-[*erythro*-*β*-*p*-hydroxyphenyl-(7''-*O*-methyl)-glyceryl] ether (peak 40).


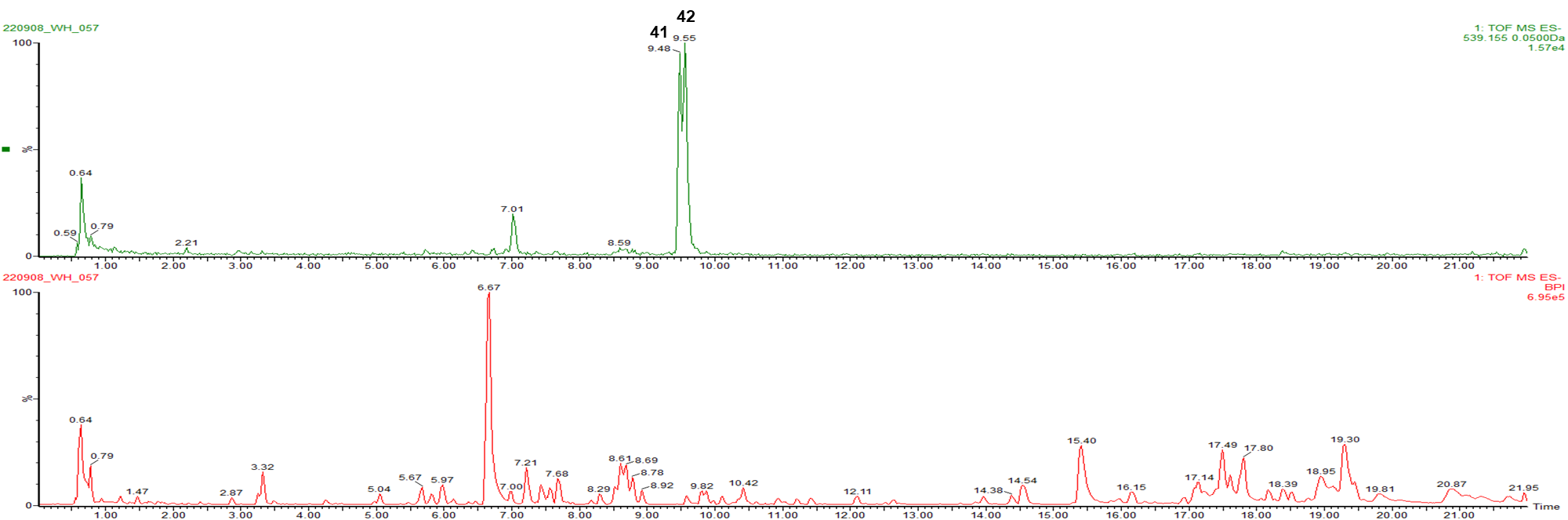


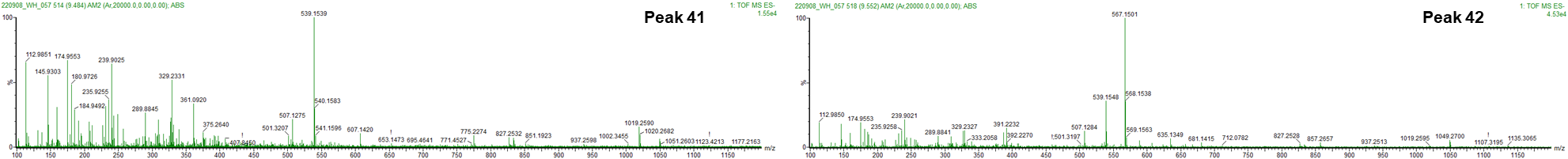


**Supplementary Figure 59.** The extracted ion chromatogram for *m/z* 539.155 and MS spectra of peaks 41 and 42.


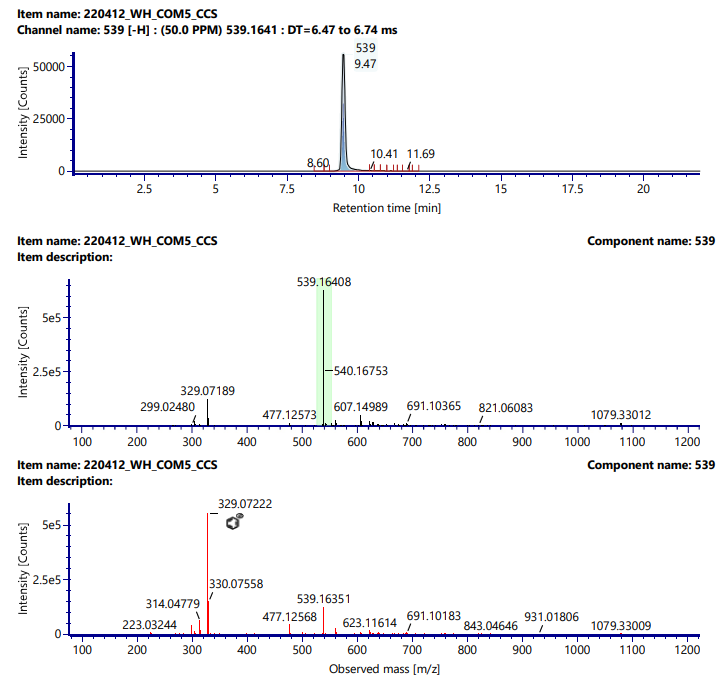


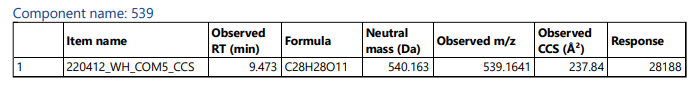


**Supplementary Figure 60.** High definition MS^E^ spectrum and collision cross section value (CCS, Å^2^) of tricin 4'-*O*-[*threo*-*β*-guaiacyl-(7"-*O*-methyl)-glyceryl] ether (peak 41).


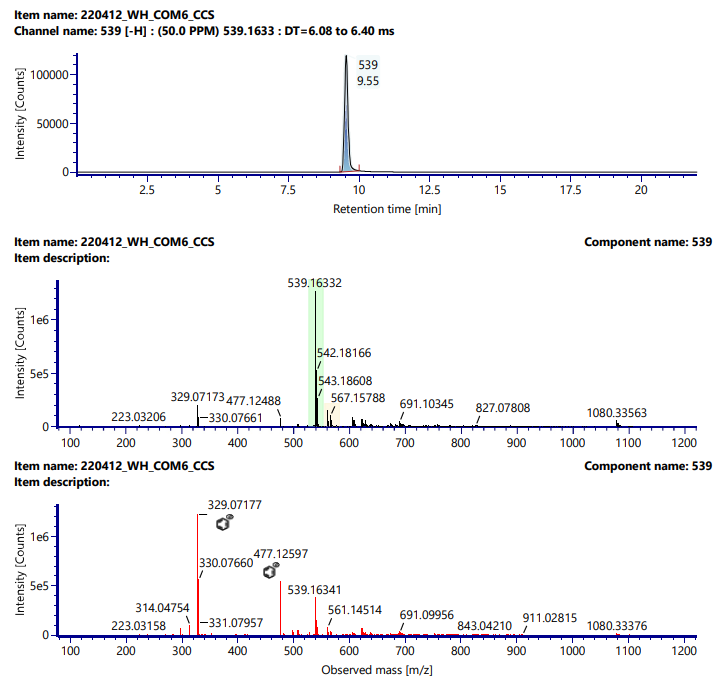


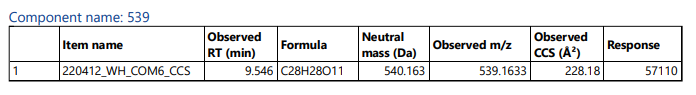


**Supplementary Figure 61.** High definition MS^E^ spectrum and collision cross section value (CCS, Å^2^) of tricin 4'-*O*-[*eythro*-*β*-guaiacyl-(7"-*O*-methyl)-glyceryl] ether (peak 42).


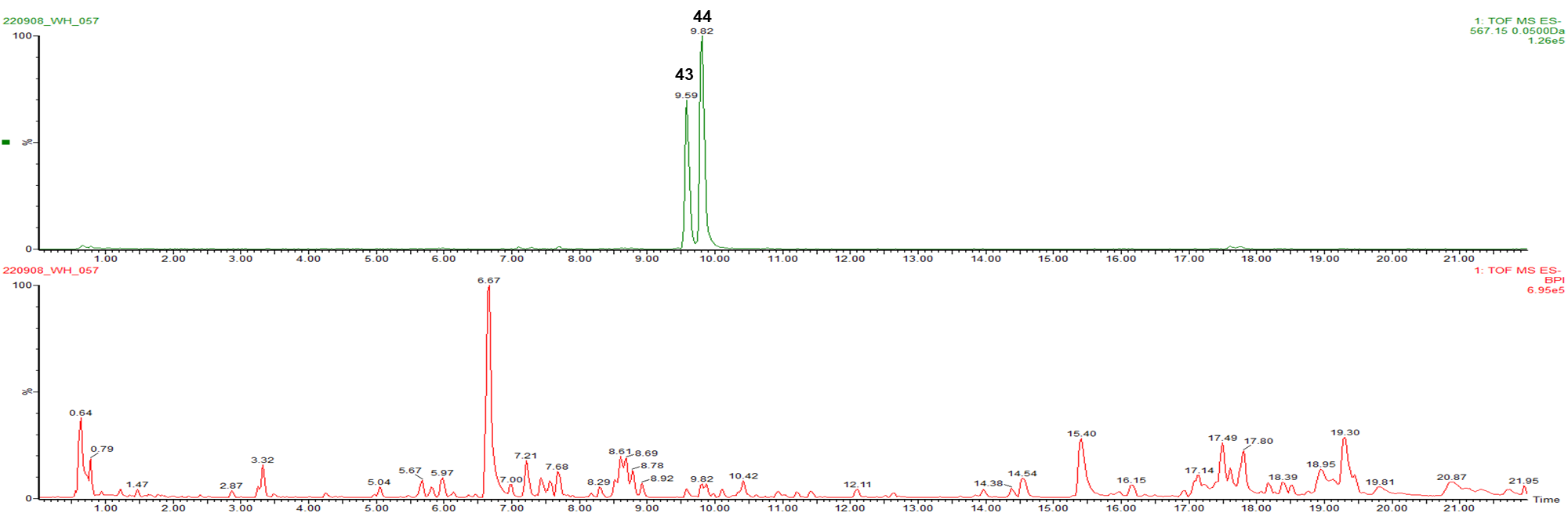


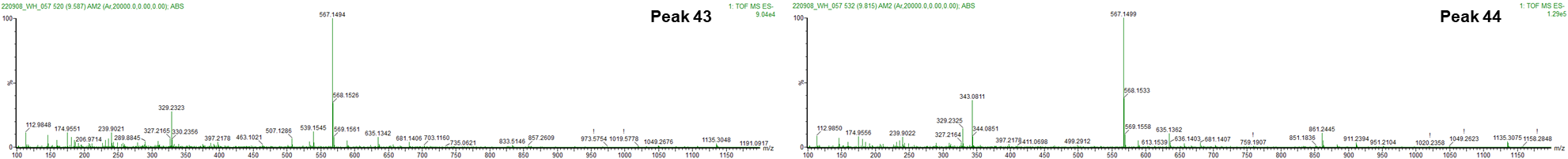


**Supplementary Figure 62.** The extracted ion chromatogram for *m/z* 567.150 and MS spectra of peaks 43 and 44.


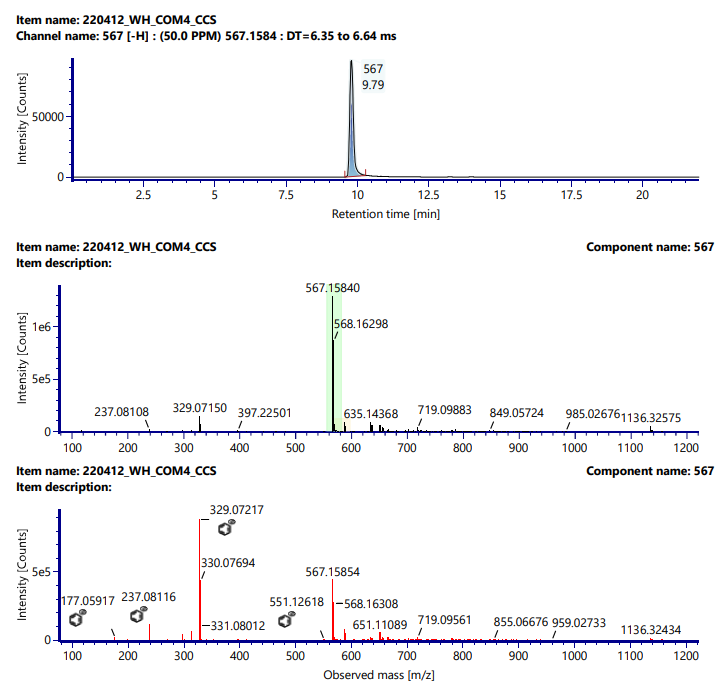


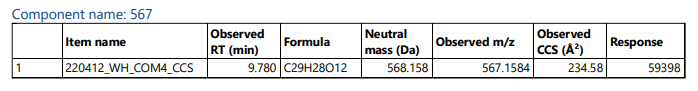


**Supplementary Figure 63.** High definition MS^E^ spectrum and collision cross section value (CCS, Å^2^) of tricin 4'-*O*-[*threo*-*β*-guaiacyl-(9"-*O*-acetyl)-glyceryl] ether (peak 44).


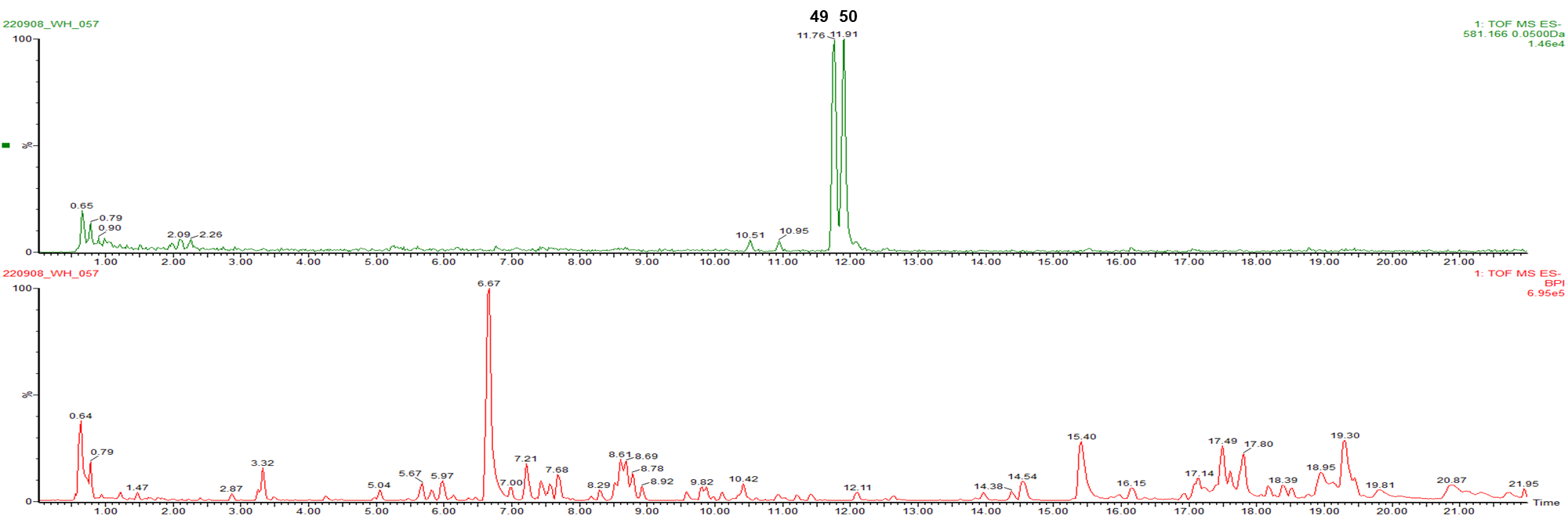


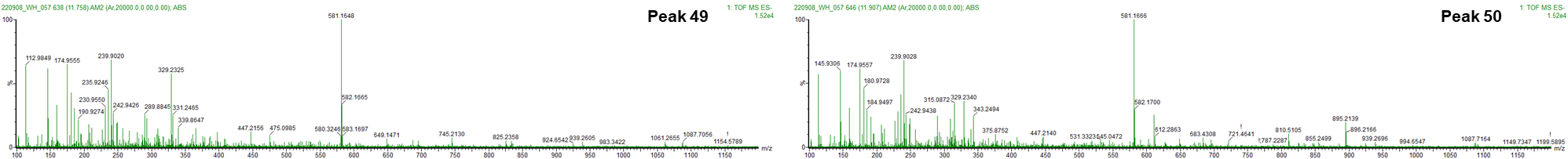


**Supplementary Figure 64.** The extracted ion chromatogram for *m/z* 581.166 and MS spectra of peaks 49 and 50.


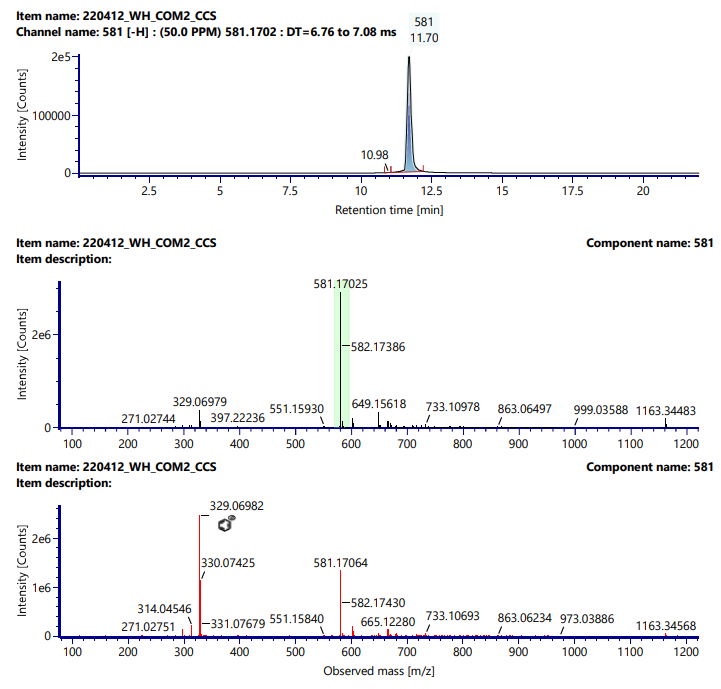

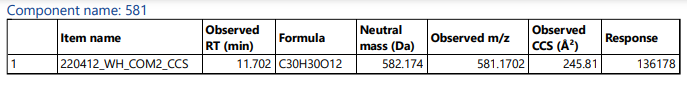


**Supplementary Figure 65.** High definition MS^E^ spectrum and collision cross section value (CCS, Å^2^) of tricin 4'-*O*-[*threo*-*β*-guaiacyl-(7"-*O*-methyl-9"-*O*-acetyl)-glyceryl] ether (peak 49).


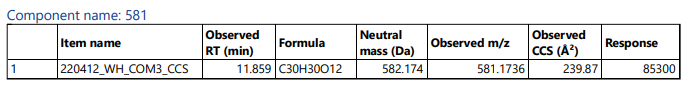

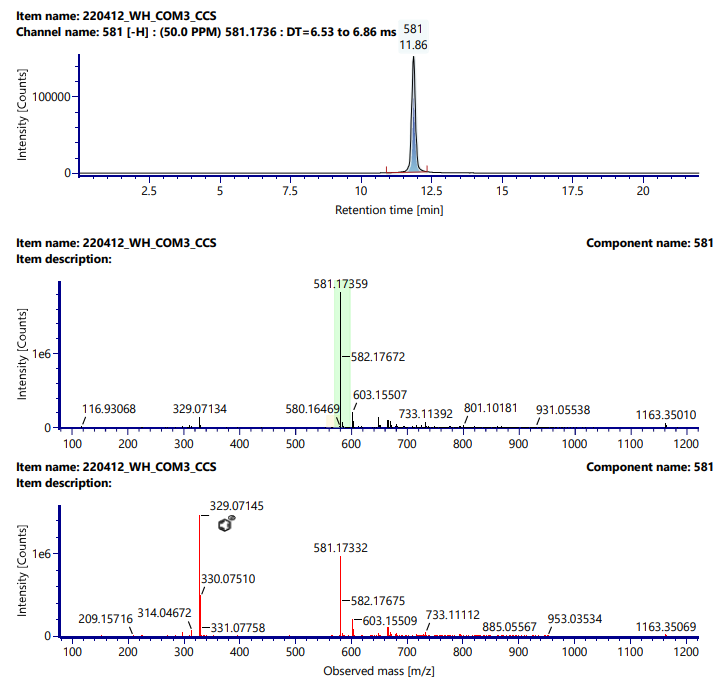


**Supplementary Figure 66.** High definition MS^E^ spectrum and collision cross section value (CCS, Å^2^) of tricin 4'-*O*-[*erythro*-*β*-guaiacyl-(7"-*O*-methyl-9"-*O*-acetyl)-glyceryl] ether (peak 50).


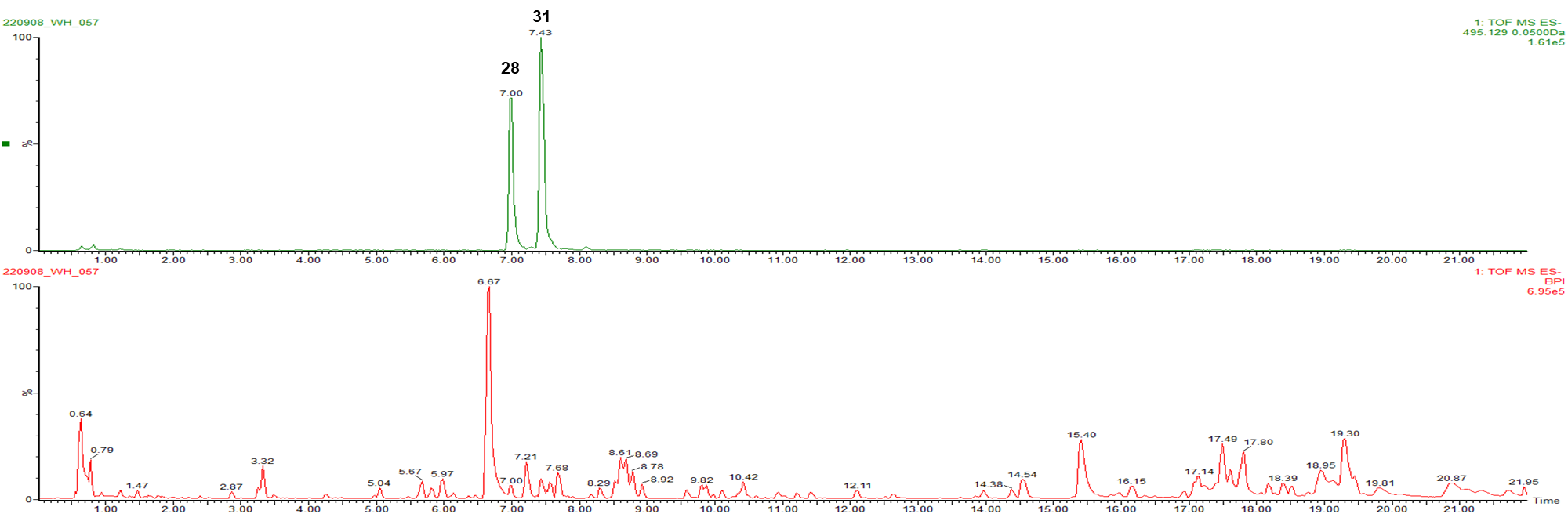


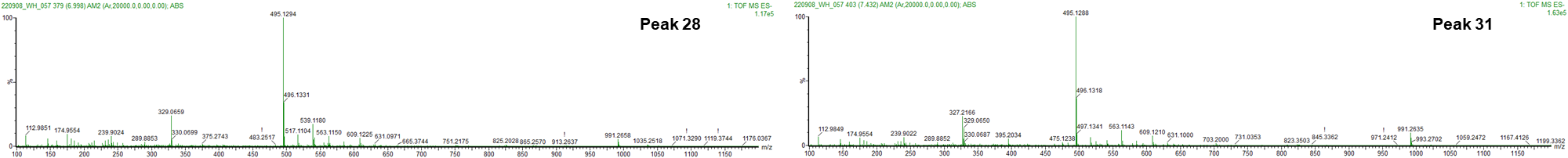


**Supplementary Figure 67.** The extracted ion chromatogram for *m/z* 495.129 and MS spectra of peaks 28 and 31.


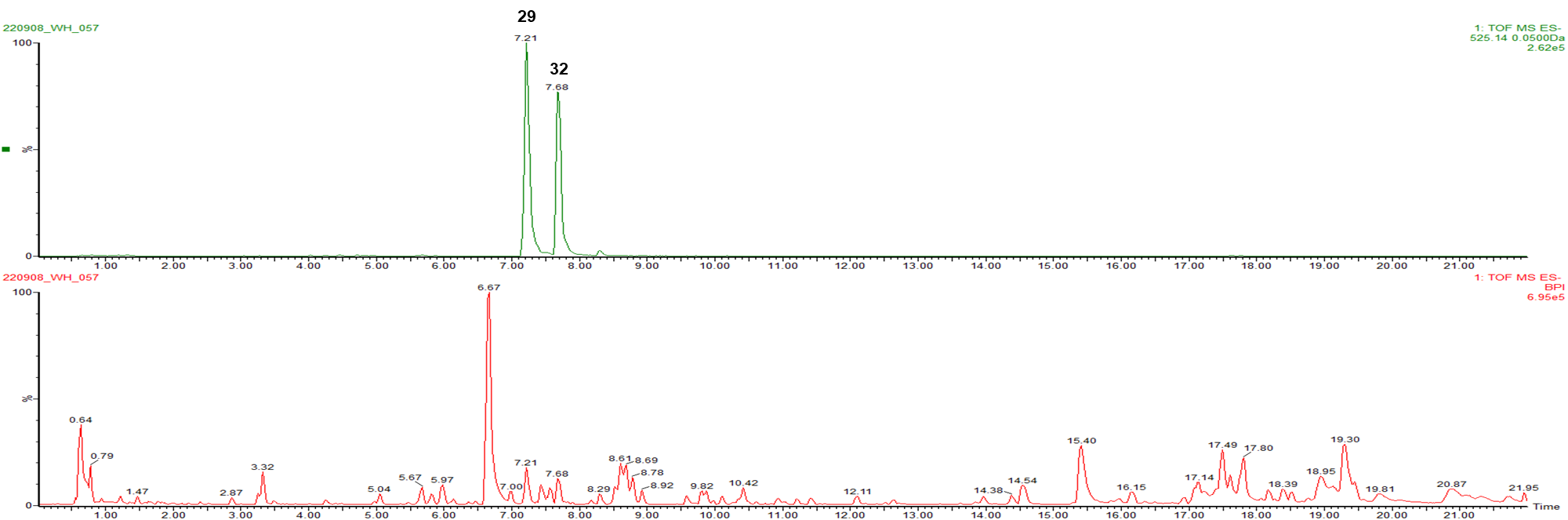


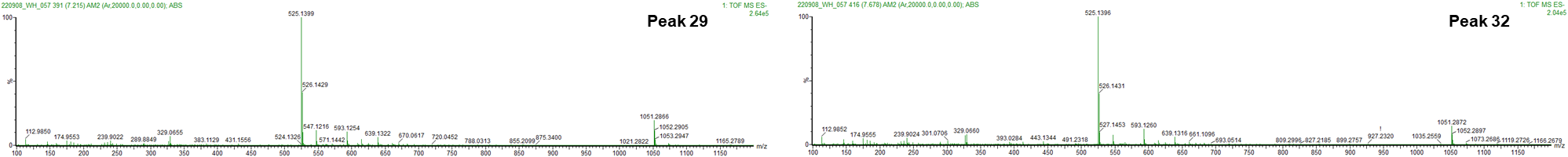


**Supplementary Figure 68.** The extracted ion chromatogram for *m/z* 525.140 and MS spectra of peaks 29 and 32.


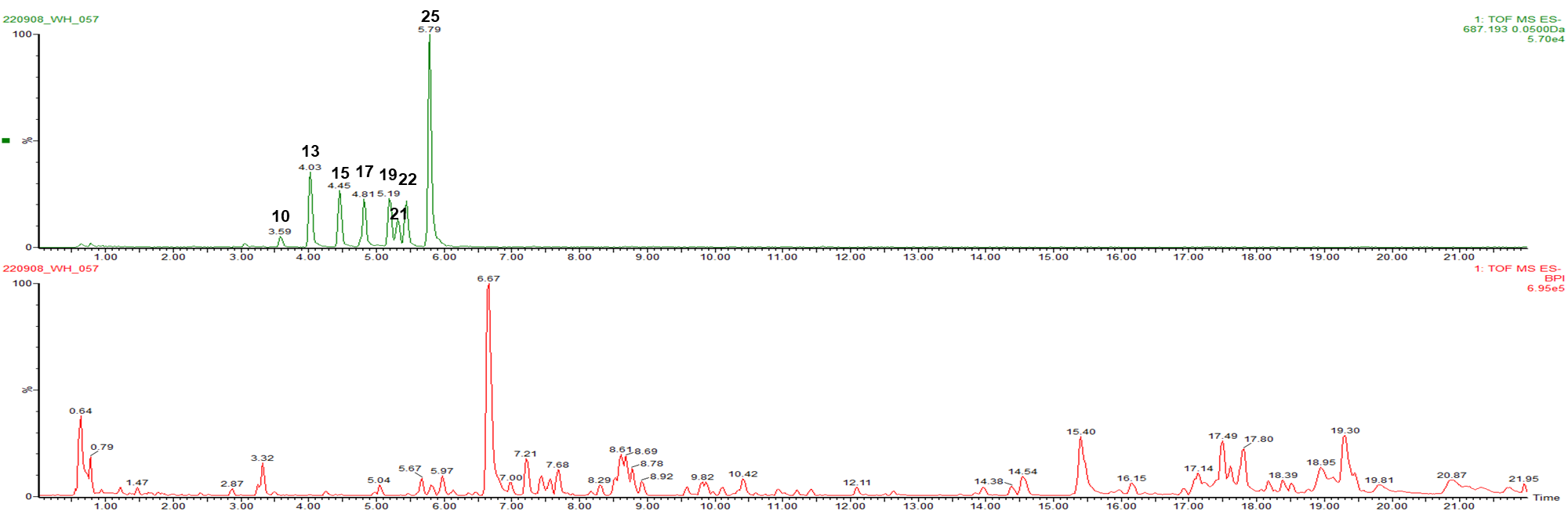


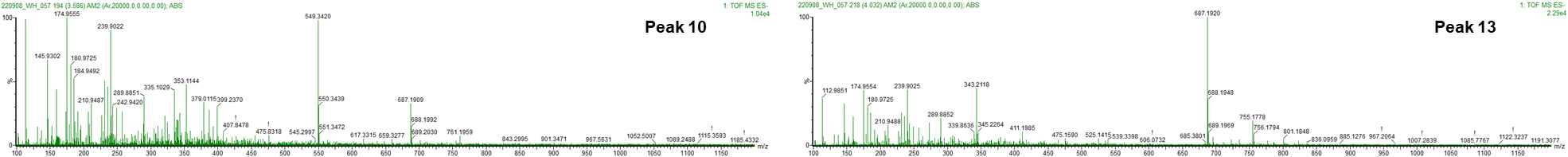


**Supplementary Figure 69.** The extracted ion chromatogram for *m/z* 687.193 and MS spectra of peaks 10, 13, 15, 17, 19, 21, 22, and 25.


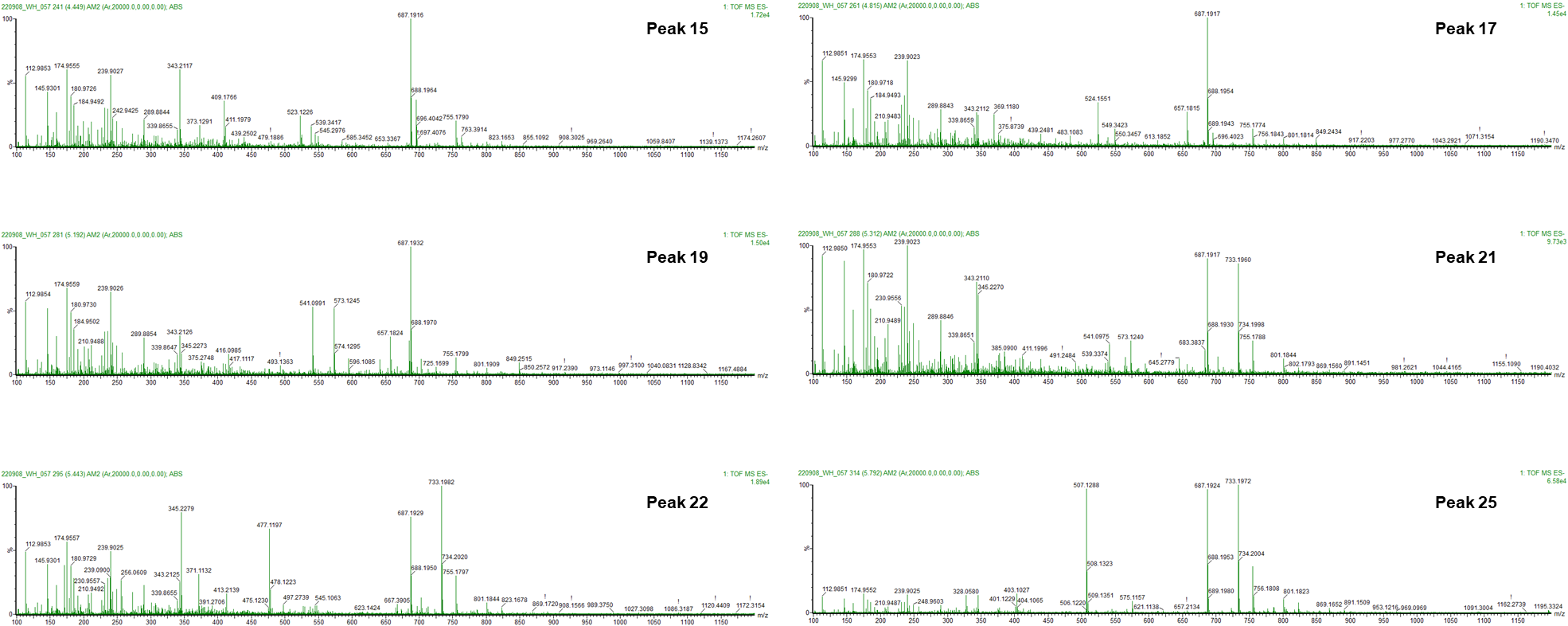


**Supplementary Figure 69.** The extracted ion chromatogram for *m/z* 687.193 and MS spectra of peaks 10, 13, 15, 17, 19, 21, 22, and 25. (*Cont.*).


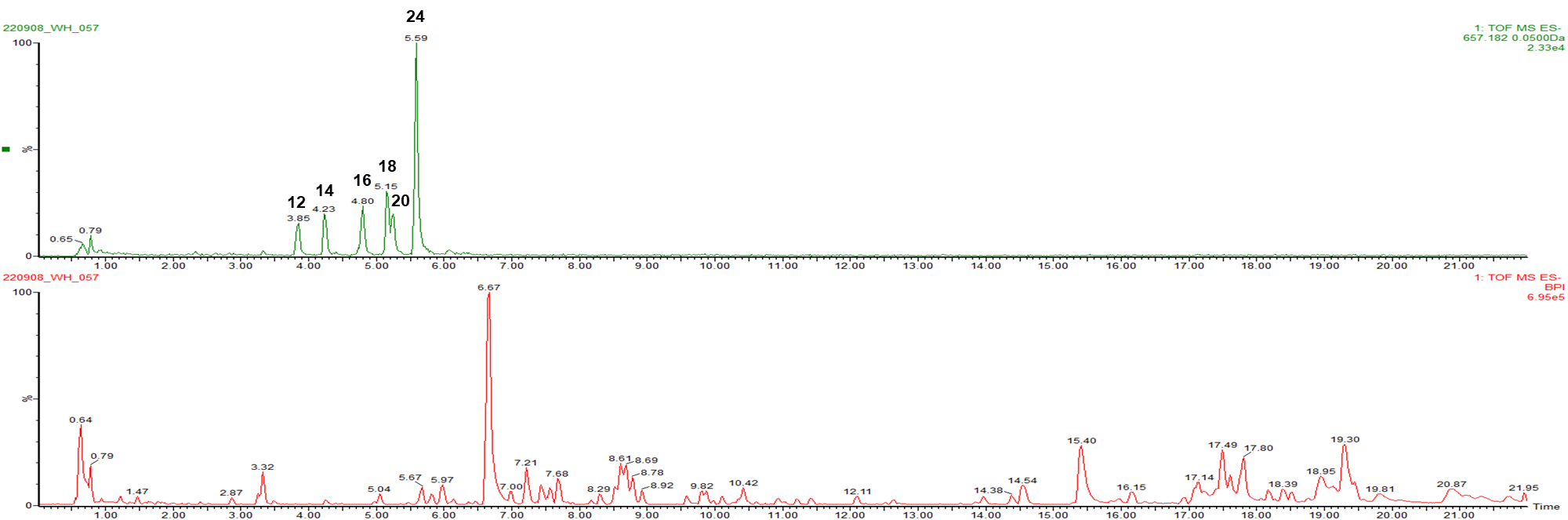


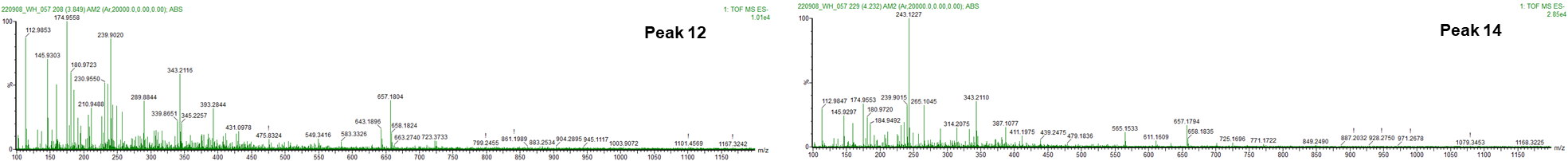


**Supplementary Figure 70.** The extracted ion chromatogram for *m/z* 657.182 and MS spectra of peaks 12, 14, 16, 18, 20, and 24.


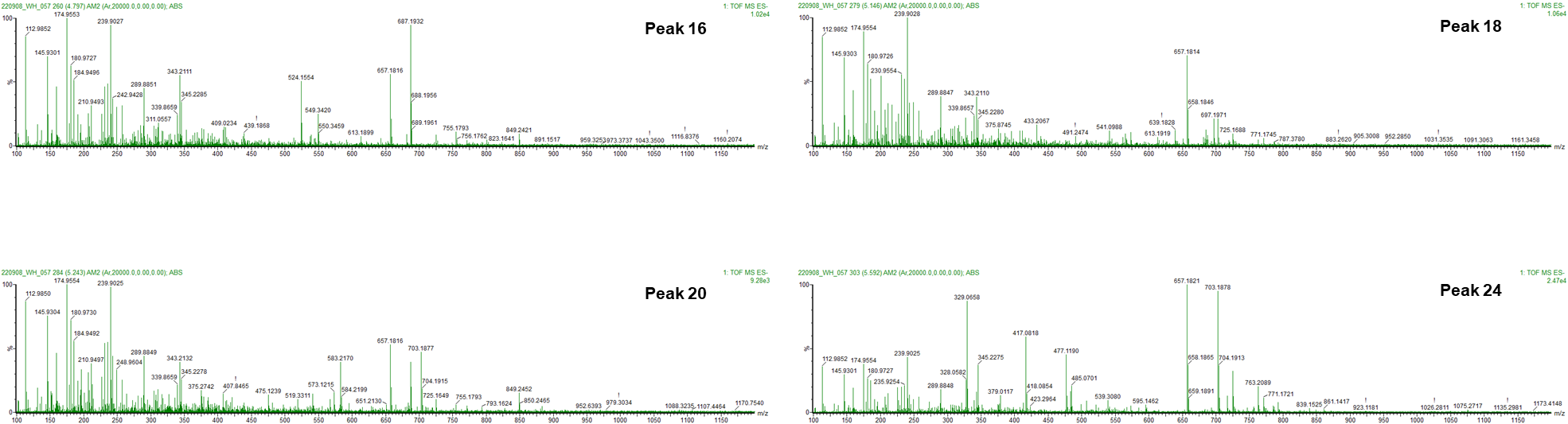


**Supplementary Figure 70.** The extracted ion chromatogram for *m/z* 657.182 and MS spectra of peaks 12, 14, 16, 18, 20, and 24 (*Cont.*).


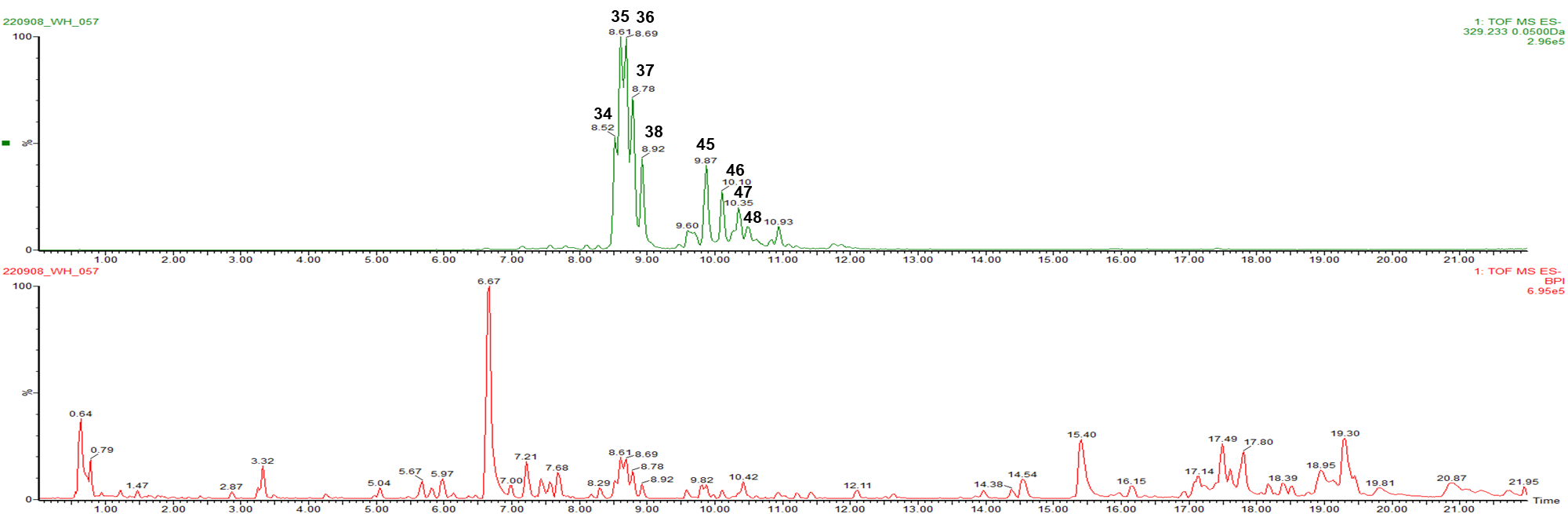


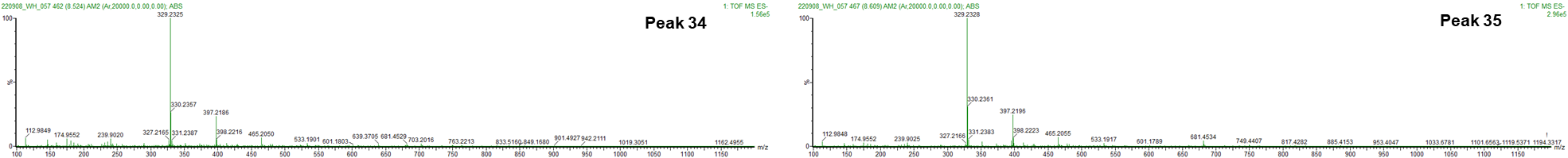


**Supplementary Figure 71.** The extracted ion chromatogram for *m/z* 329.233 and MS spectra of peaks 34‒38 and 45‒48.


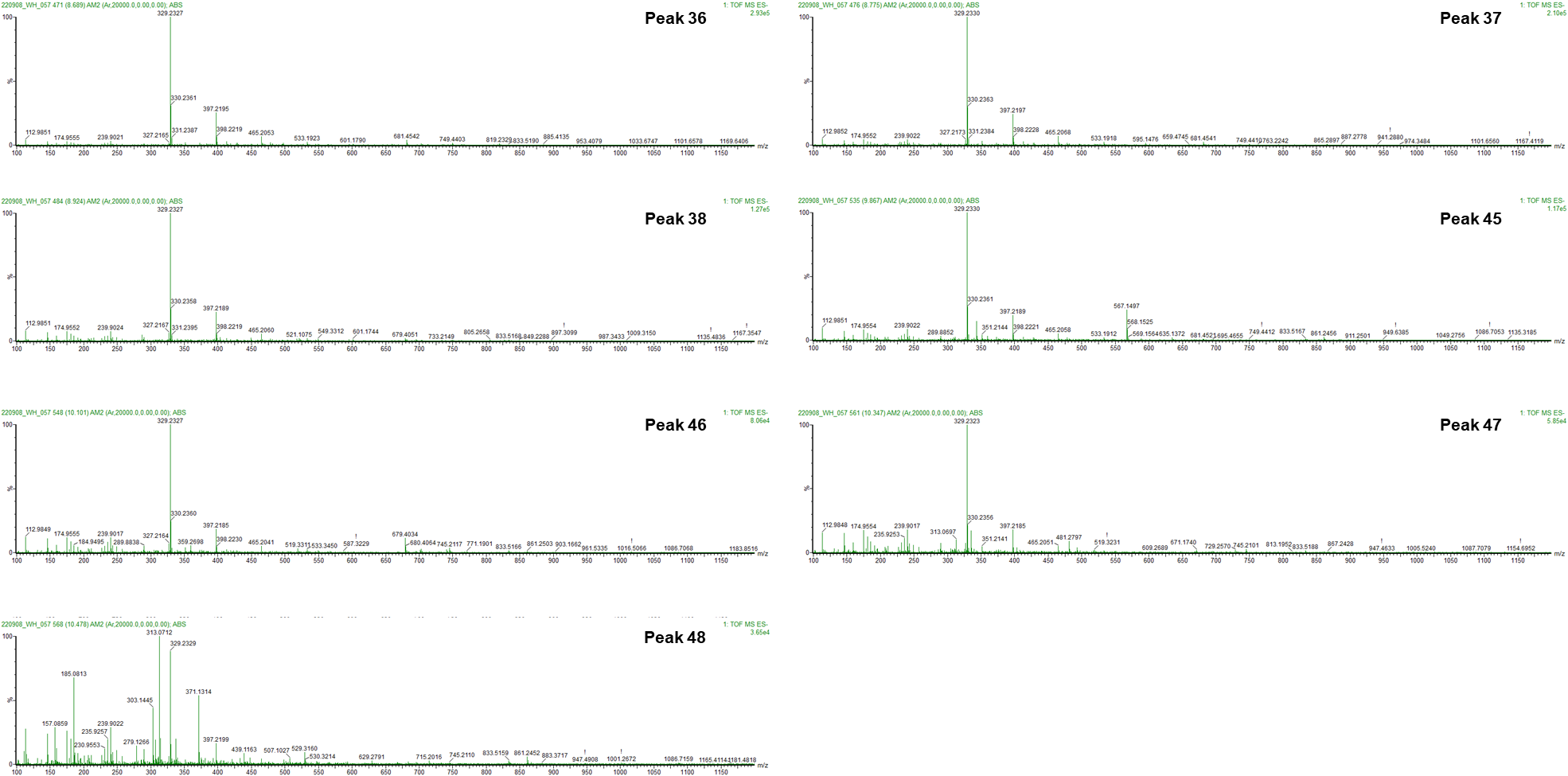


**Supplementary Figure 71.** The extracted ion chromatogram for *m/z* 329.233 and MS spectra of peaks 34‒38 and 45‒48 (*Cont.*).


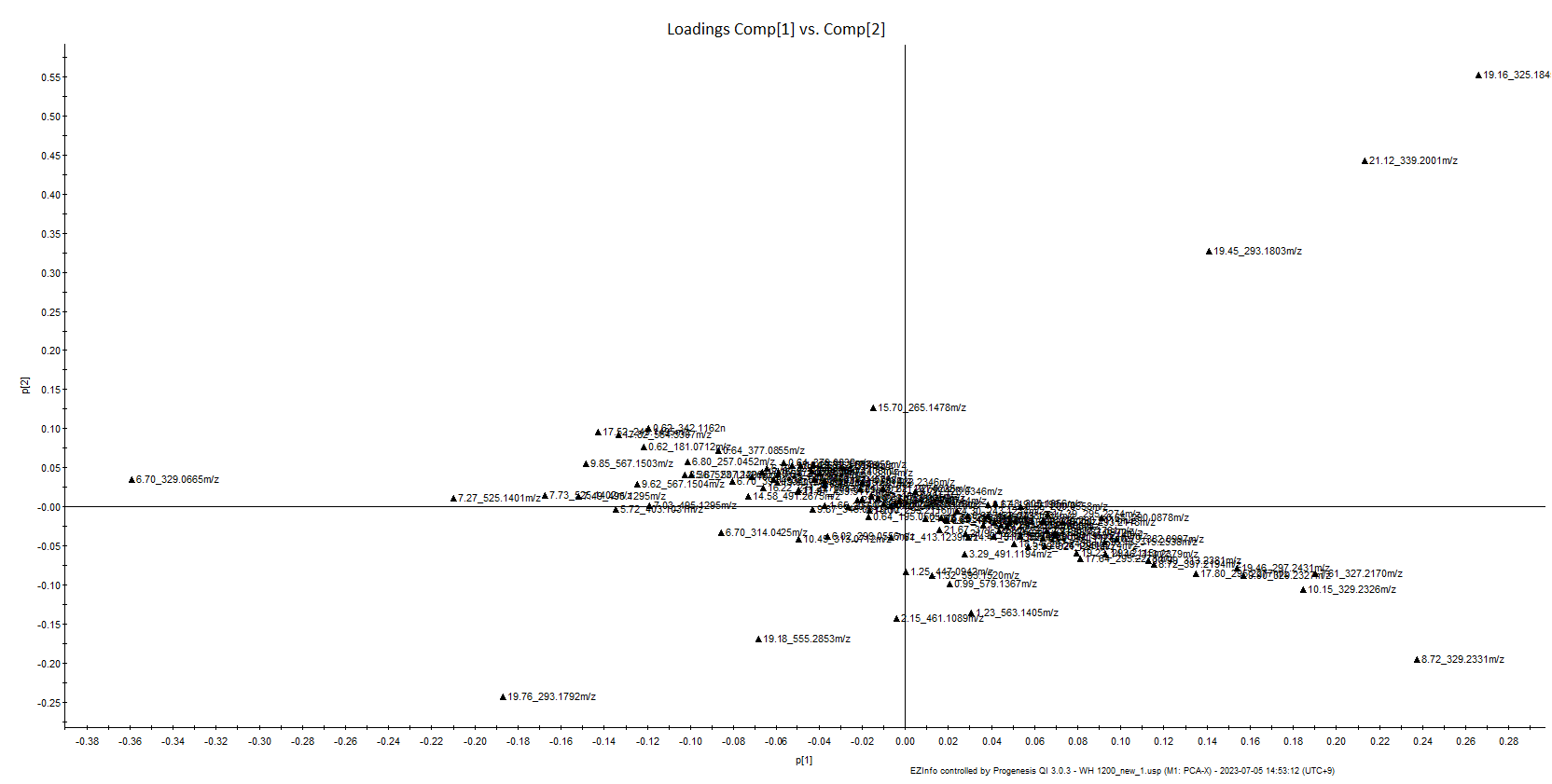


**Supplementary Figure 72.** Principal component analysis (PCA) loading plot of metabolome analysis of the 983 wheat hull samples.

**
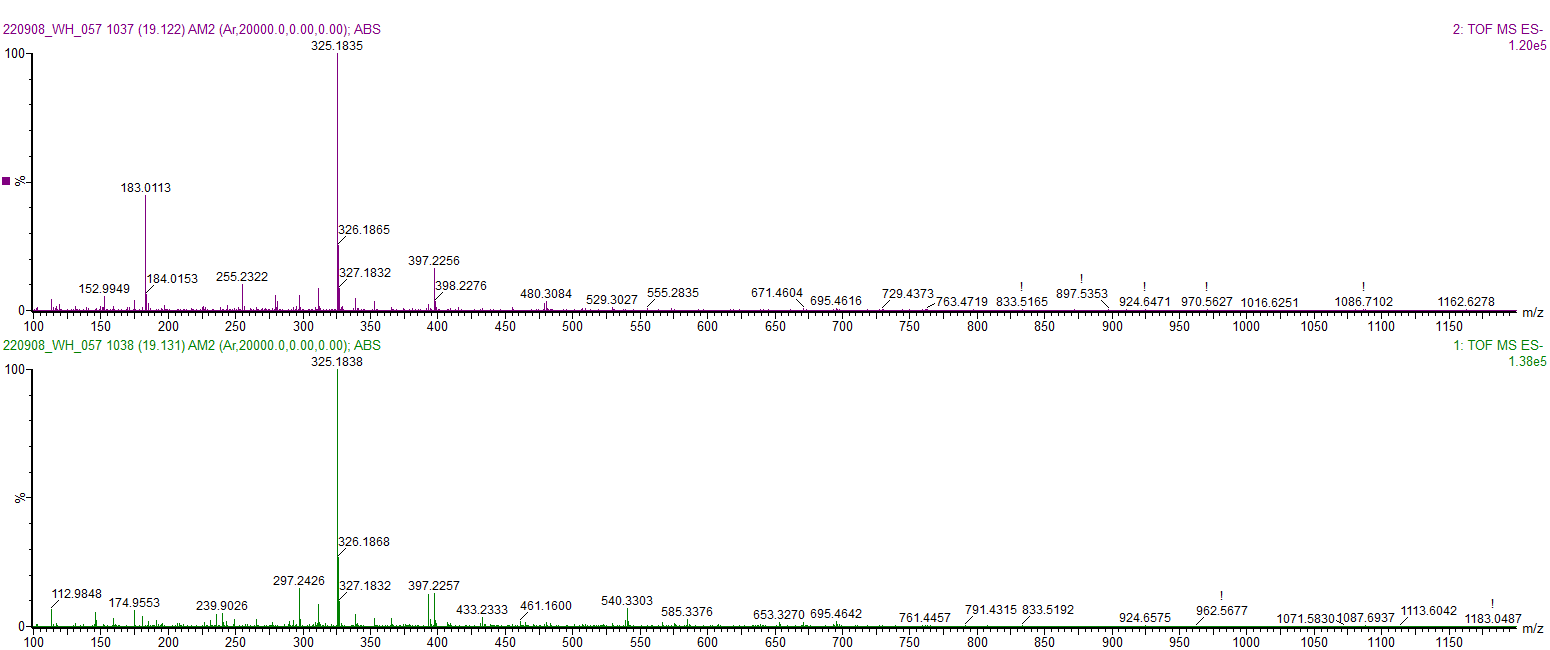
**

**Supplementary Figure 73.** ESI-QTof-MS spectrum of heptaethylene glycol (marker 1).


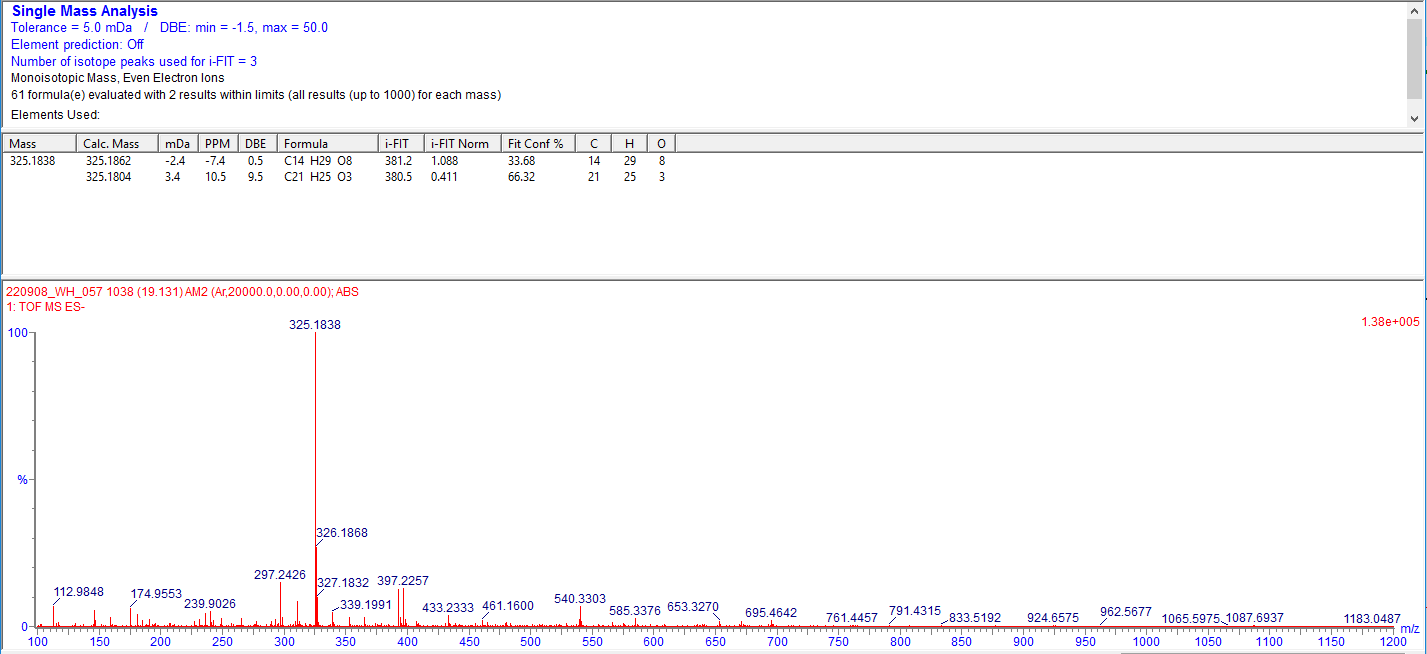


**Supplementary Figure 74.** Molecular formula for HRMS ion value of maker 1, which is analyzed in MassLynx (Waters Corporation, Milford, MA, USA).

**
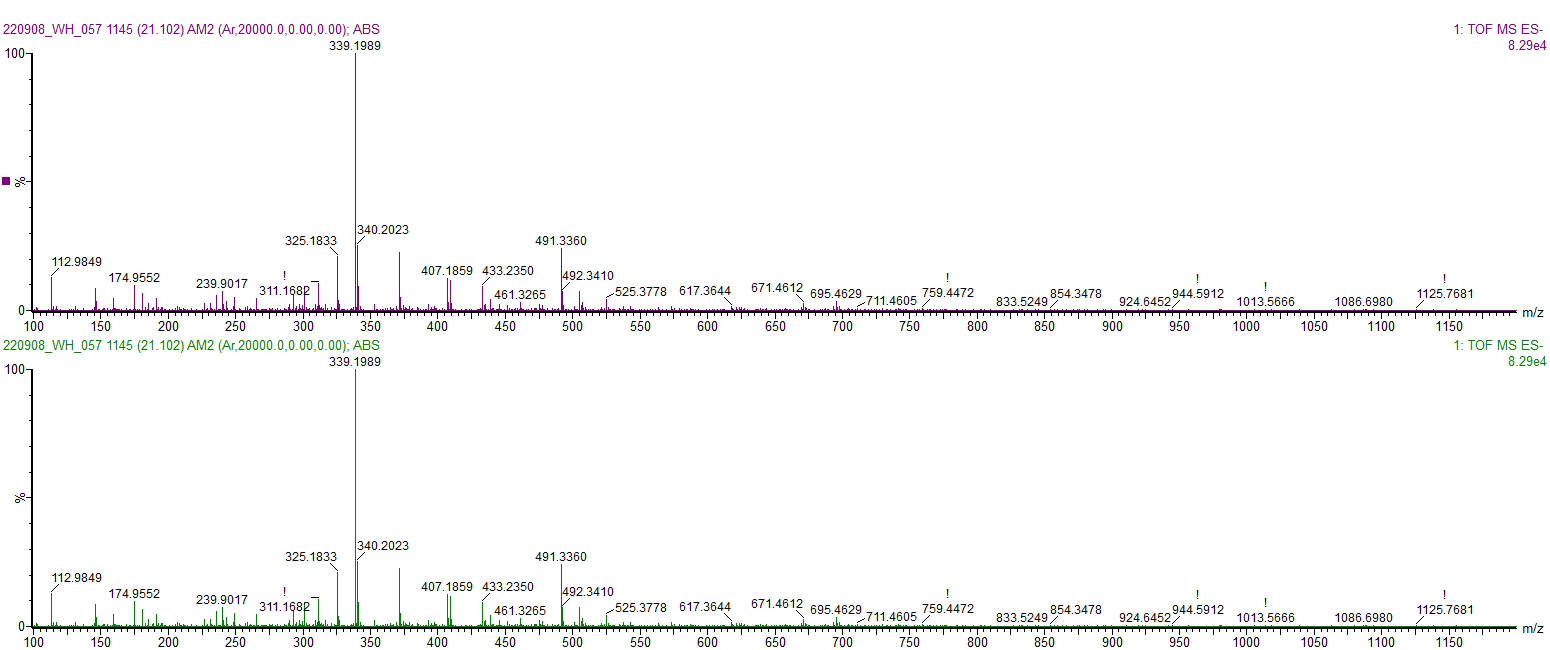
**

**Supplementary Figure 75.** ESI-QTof-MS spectrum of heptaethylene glycol monomethyl ether (marker 2).


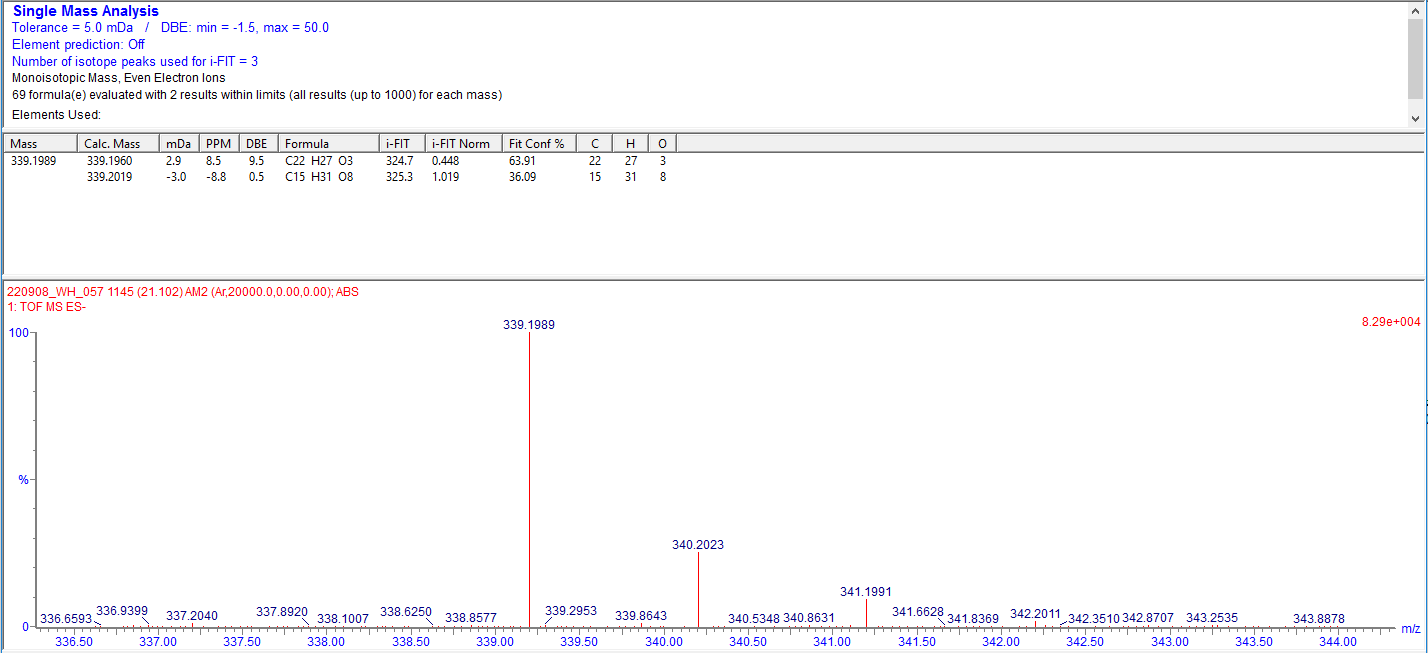


**Supplementary Figure 76.** Molecular formula for HRMS ion value of marker 2, which is analyzed in MassLynx (Waters Corporation, Milford, MA, USA).


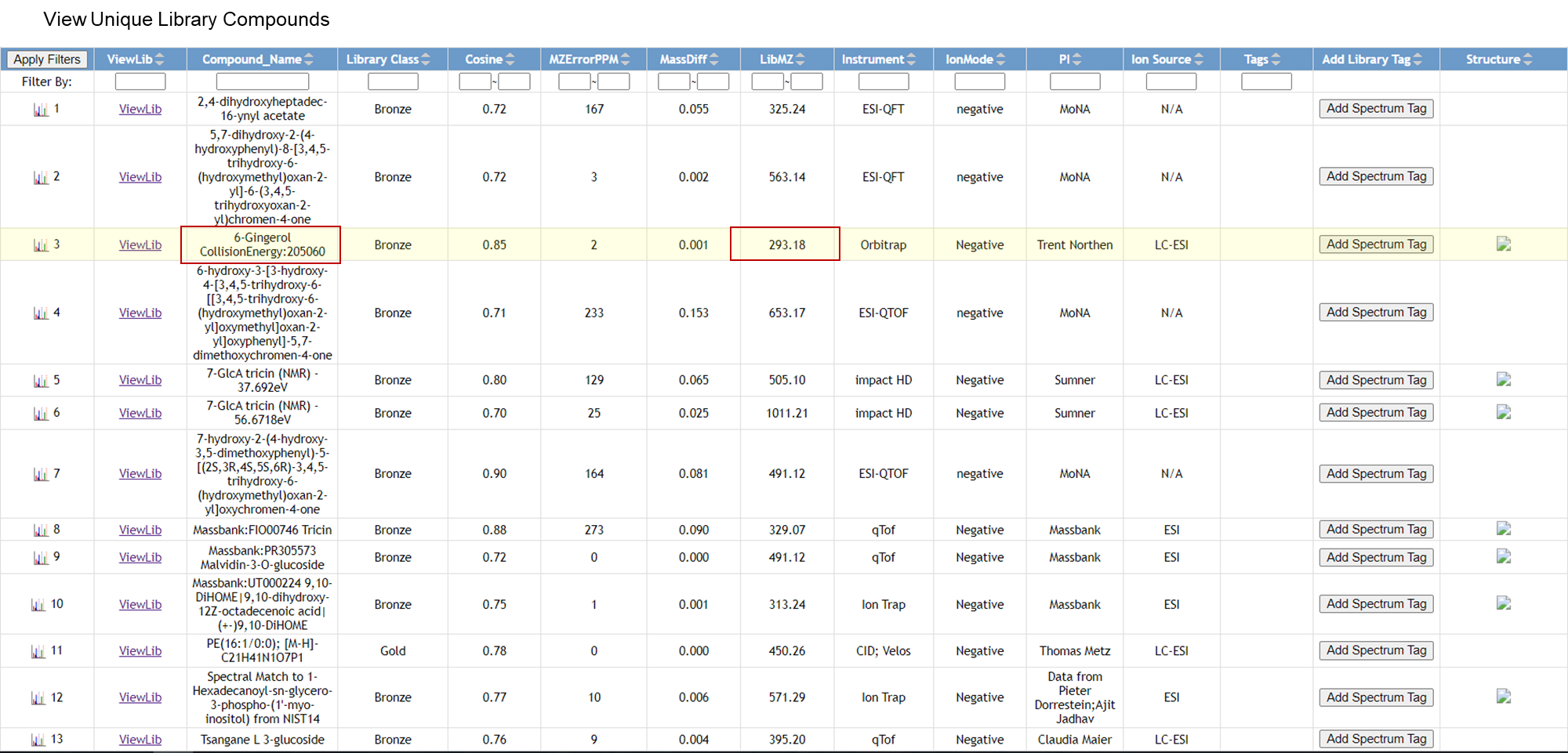


**Supplementary Figure 77.** Compounds list extracted from database of Global Natural Product Social Networking (GNPS).

**
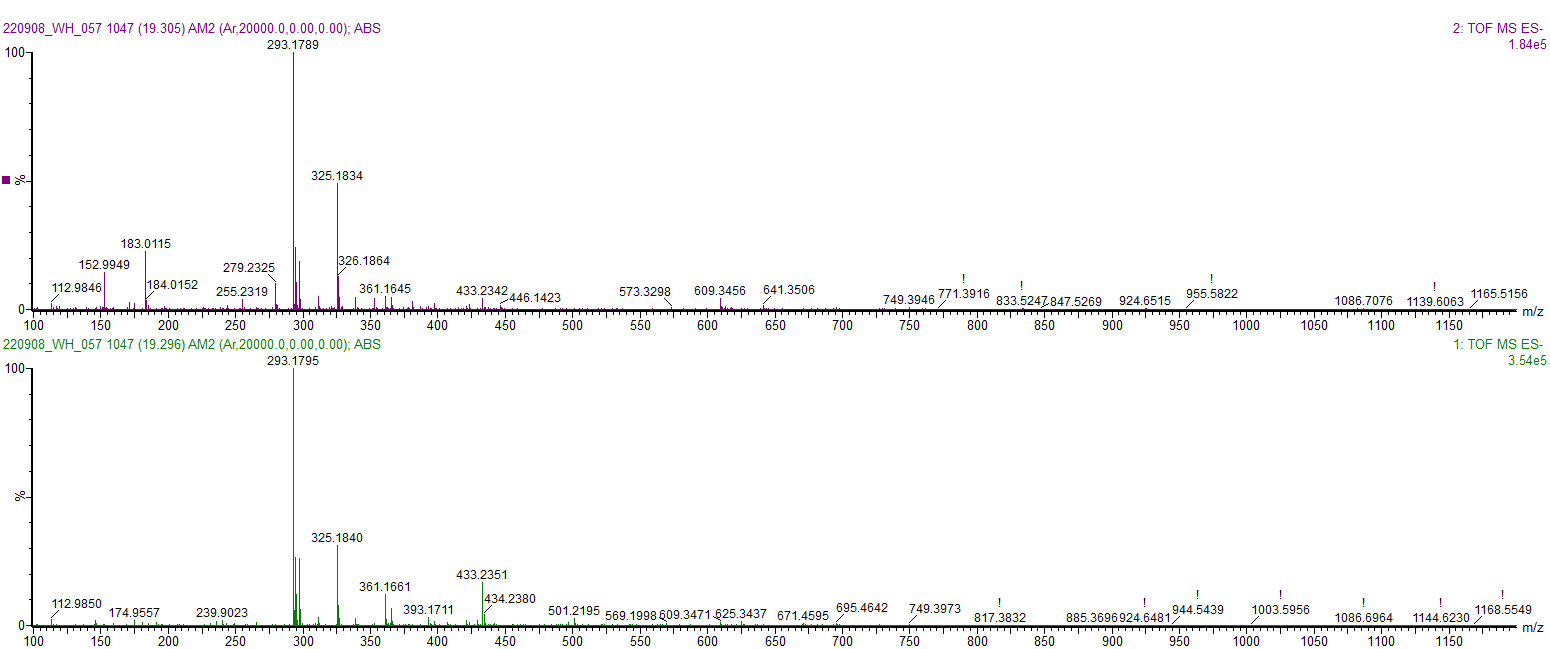
**

**Supplementary Figure 78.** ESI-QTof-MS spectrum of 6-gingerol (marker 3).

**
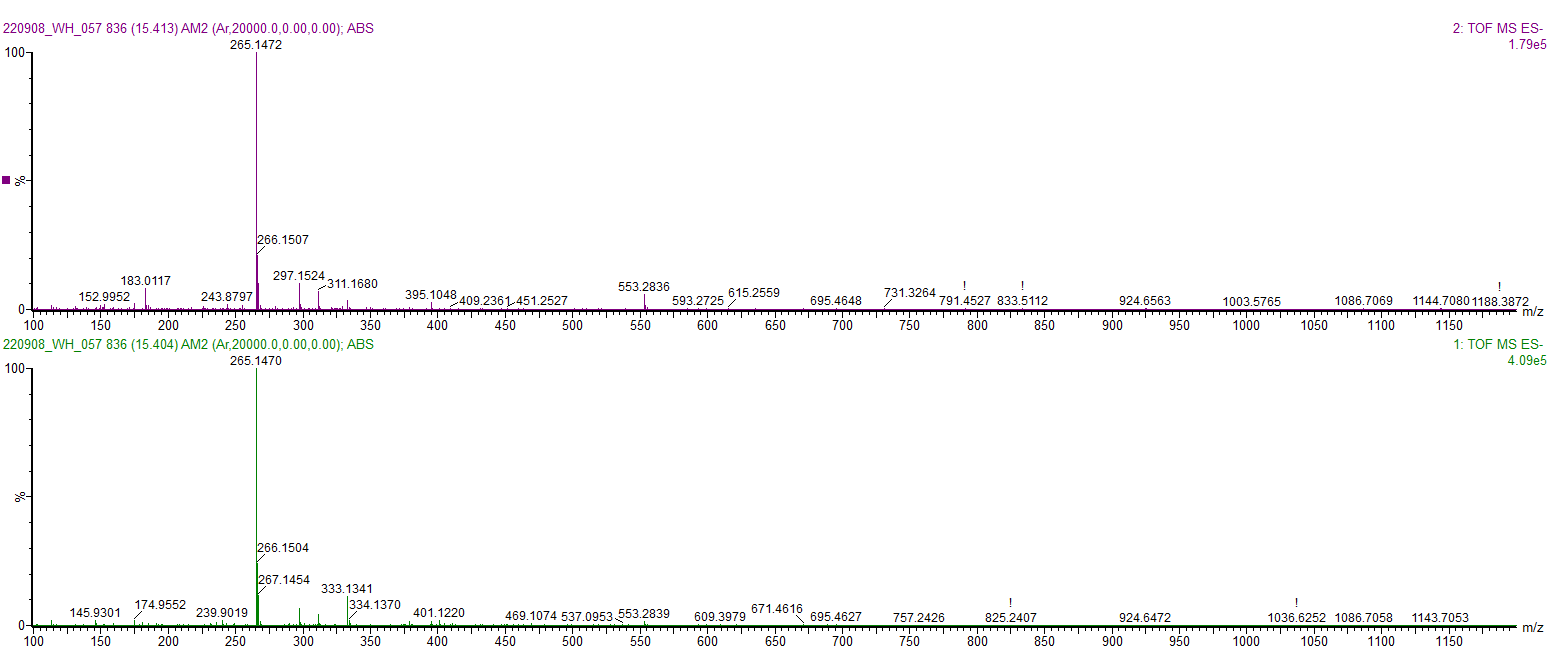
**

**Supplementary Figure 79.** ESI-QTof-MS spectrum of 4-gingerol (marker 4).

**
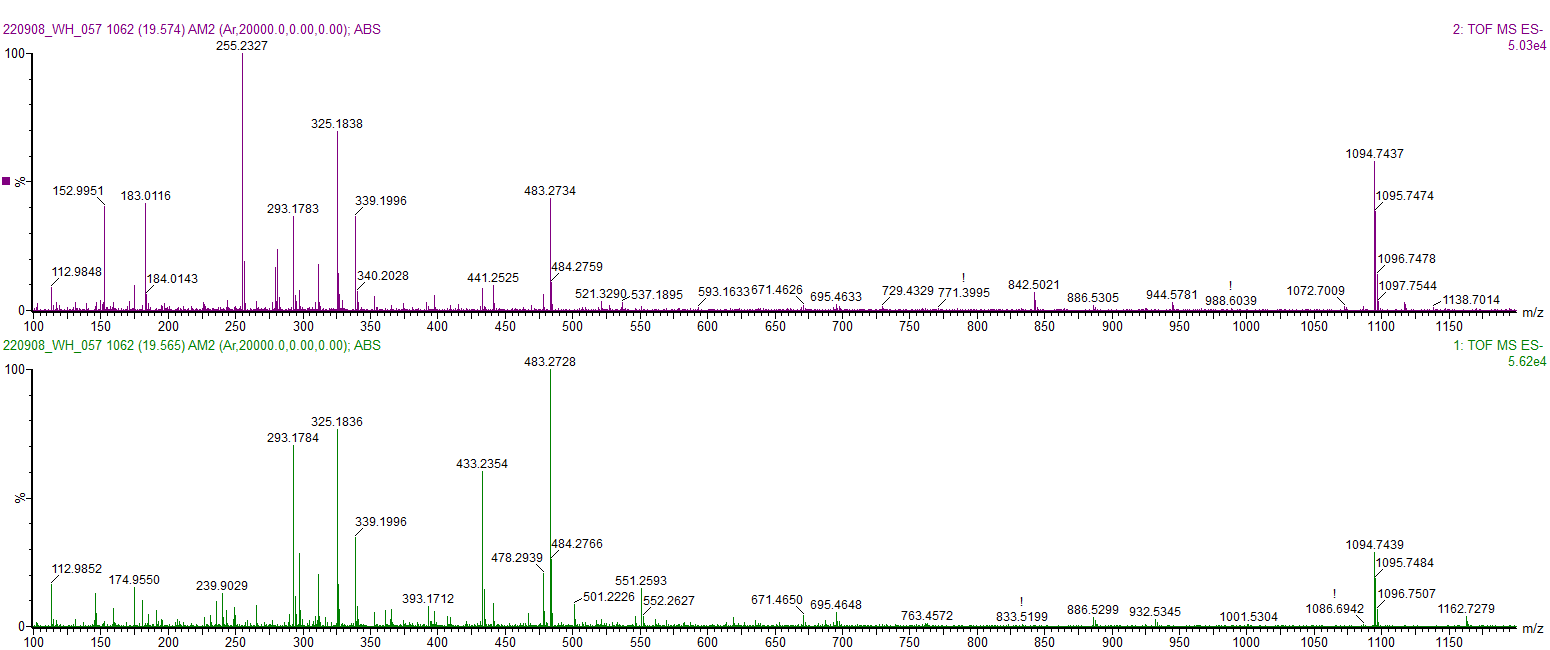
**

**Supplementary Figure 80.** ESI-QTof-MS spectrum of phytuberin (marker 5).


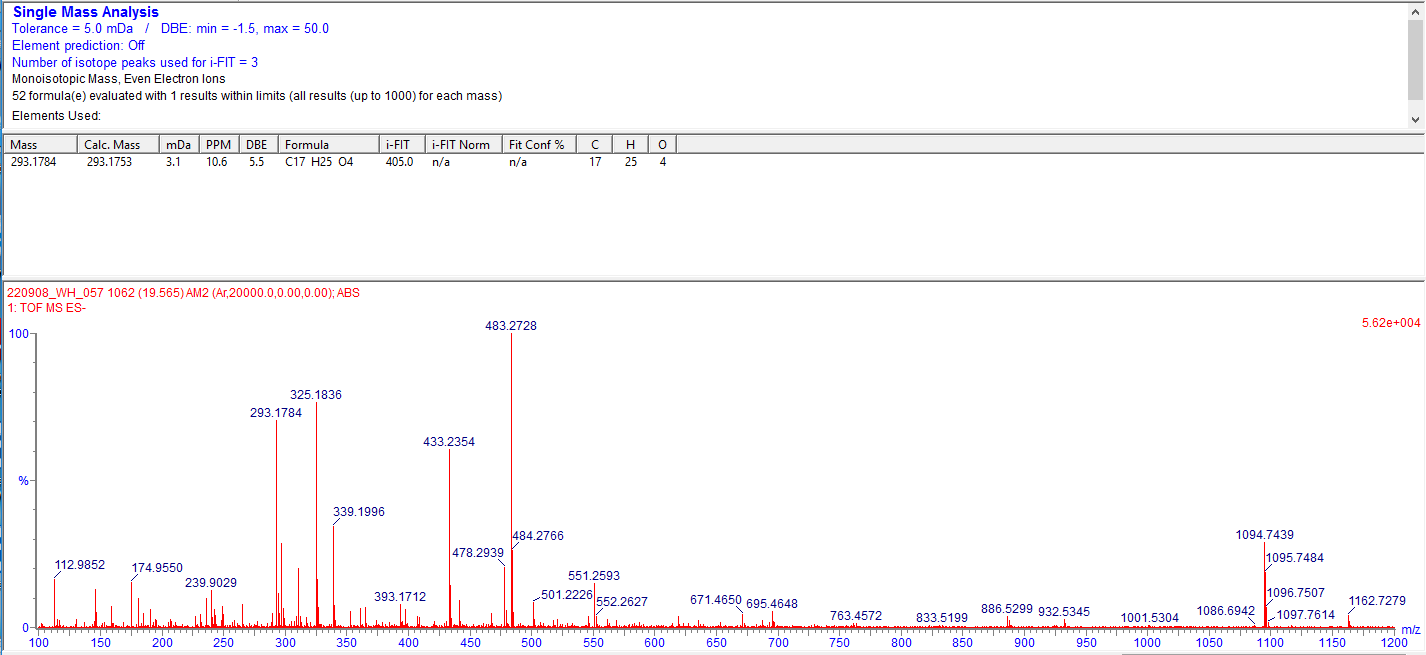


**Supplementary Figure 81.** Molecular formula for HRMS ion value of marker 5, which is analyzed in MassLynx (Waters Corporation, Milford, MA, USA).

**Supplementary Figure 82.** ESI-QTof-MS spectrum of unknown (marker 6).

**Supplementary Figure 83.** Molecular formula for HRMS ion value of marker 6, which is analyzed in MassLynx (Waters Corporation, Milford, MA, USA).

**Supplementary Figure 84.** Orthogonal partial least-squares discriminant analysis (OPLS-DA) S-plot between active and inactive groups.

**Supplementary Figure 85.** Orthogonal partial least-squares discriminant analysis (OPLS-DA) variable importance in projection (VIP) plot between active and inactive groups.

**Supplementary Table 1.** Nitric oxide (NO) production (% of cell viability) of the wheat hull of two original cultivars (WH01 and WH02) and its 983 mutant lines in lipopolysaccharide-stimulated RAW 264.7 macrophage cells. The values are expressed as the mean ± SD of three independent experiments.

**Supplementary Table 1.** Nitric oxide (NO) production (% of cell viability) of the wheat hull of two original cultivars (WH01 and WH02) and its 983 mutant lines in lipopolysaccharide-stimulated RAW 264.7 macrophage cells. The values are expressed as the mean ± SD of three independent experiments (*Cont.*).

**Supplementary Table 1.** Nitric oxide (NO) production (% of cell viability) of the wheat hull of two original cultivars (WH01 and WH02) and its 983 mutant lines in lipopolysaccharide-stimulated RAW 264.7 macrophage cells. The values are expressed as the mean ± SD of three independent experiments (*Cont.*).

**Supplementary Table 1.** Nitric oxide (NO) production (% of cell viability) of the wheat hull of two original cultivars (WH01 and WH02) and its 983 mutant lines in lipopolysaccharide-stimulated RAW 264.7 macrophage cells. The values are expressed as the mean ± SD of three independent experiments (*Cont.*).

**Supplementary Table 1.** Nitric oxide (NO) production (% of cell viability) of the wheat hull of two original cultivars (WH01 and WH02) and its 983 mutant lines in lipopolysaccharide-stimulated RAW 264.7 macrophage cells. The values are expressed as the mean ± SD of three independent experiments (*Cont.*).

**Supplementary Table 1.** Nitric oxide (NO) production (% of cell viability) of the wheat hull of two original cultivars (WH01 and WH02) and its 983 mutant lines in lipopolysaccharide-stimulated RAW 264.7 macrophage cells. The values are expressed as the mean ± SD of three independent experiments (*Cont.*).

**Supplementary Table 1.** Nitric oxide (NO) production (% of cell viability) of the wheat hull of two original cultivars (WH01 and WH02) and its 983 mutant lines in lipopolysaccharide-stimulated RAW 264.7 macrophage cells. The values are expressed as the mean ± SD of three independent experiments (*Cont.*).
